# Gene duplication and the origins of morphological complexity in pancrustacean eyes, a genomic approach

**DOI:** 10.1186/1471-2148-10-123

**Published:** 2010-04-30

**Authors:** Ajna S Rivera, M Sabrina Pankey, David C Plachetzki, Carlos Villacorta, Anna E Syme, Jeanne M Serb, Angela R Omilian, Todd H Oakley

**Affiliations:** 1 Ecology Evolution and Marine Biology, University of California Santa Barbara, Santa Barbara, CA 93106 USA; 2 Department of Biology, Jordan Hall 1001 E. Third Street, Indiana University, Bloomington, IN USA; 3 Department of Ecology, Evolution, and Organismal Biology, 245 Bessey Hall, Iowa State University, Ames, IA 50011 USA

## Abstract

**Background:**

Duplication and divergence of genes and genetic networks is hypothesized to be a major driver of the evolution of complexity and novel features. Here, we examine the history of genes and genetic networks in the context of eye evolution by using new approaches to understand patterns of gene duplication during the evolution of metazoan genomes. We hypothesize that 1) genes involved in eye development and phototransduction have duplicated and are retained at higher rates in animal clades that possess more distinct types of optical design; and 2) genes with functional relationships were duplicated and lost together, thereby preserving genetic networks. To test these hypotheses, we examine the rates and patterns of gene duplication and loss evident in 19 metazoan genomes, including that of *Daphnia pulex *- the first completely sequenced crustacean genome. This is of particular interest because the pancrustaceans (hexapods+crustaceans) have more optical designs than any other major clade of animals, allowing us to test specifically whether the high amount of disparity in pancrustacean eyes is correlated with a higher rate of duplication and retention of vision genes.

**Results:**

Using protein predictions from 19 metazoan whole-genome projects, we found all members of 23 gene families known to be involved in eye development or phototransduction and deduced their phylogenetic relationships. This allowed us to estimate the number and timing of gene duplication and loss events in these gene families during animal evolution. When comparing duplication/retention rates of these genes, we found that the rate was significantly higher in pancrustaceans than in either vertebrates or non-pancrustacean protostomes. Comparing patterns of co-duplication across Metazoa showed that while these eye-genes co-duplicate at a significantly higher rate than those within a randomly shuffled matrix, many genes with known functional relationships in model organisms did not co-duplicate more often than expected by chance.

**Conclusions:**

Overall, and when accounting for factors such as differential rates of whole-genome duplication in different groups, our results are broadly consistent with the hypothesis that genes involved in eye development and phototransduction duplicate at a higher rate in Pancrustacea, the group with the greatest variety of optical designs. The result that these genes have a significantly high number of co-duplications and co-losses could be influenced by shared functions or other unstudied factors such as synteny. Since we did not observe co-duplication/co-loss of genes for all known functional modules (e.g. specific regulatory networks), the interactions among suites of known co-functioning genes (modules) may be plastic at the temporal scale of analysis performed here. Other factors in addition to gene duplication - such as cis-regulation, heterotopy, and co-option - are also likely to be strong factors in the diversification of eye types.

## Background

Genomic complexity is driven, to a large extent, by gene duplication, retention, and divergence [1,2]. This is hypothesized to lead to both an increase in morphological complexity, via the evolution of novel features, and an increase in proteomic network complexity, through the establishment of new network interactions [3-5]. Here, we examine the genetic histories of 23 gene families involved in eye development and phototransduction to test: 1) whether gene duplication rates are higher in a taxon with greater eye disparity (we use the term disparity as it is used in paleontology to describe the diversity of morphology [6]) and 2) if genes with known functional relationships (genetic networks) tend to co-duplicate across taxa. We test these hypotheses by identifying gene-family members involved in eye development and phototransduction from metazoan full genome sequences. We use the term 'eye-genes' to describe the genes in our dataset with caution, because many of these genes have additional functions beyond vision or eye development and because it is not possible to analyze all genes that influence vision or eye development. Next, we map duplication and loss events of these eye-genes on an assumed metazoan phylogeny. We then test for an elevated rate of gene duplication/accumulation in the group with the greatest diversity of optical designs, the Pancrustacea. Finally, we search for correlation in duplication patterns among these gene families - a signature of 'co-duplication' [7].

We define Pancrustacea as disparate in eye morphology because the group has the highest number of distinct optical designs of any animal group. At the broadest level, there are eight recognized optical designs for eyes in all Metazoa [8]. Four of the broad optical types are single chambered eyes like those of vertebrates. The other four eye types are compound eyes with multiple focusing (dioptric) apparatuses, rather than the single one found in single chambered eyes. The disparity of optical designs in pancrustaceans (hexapods + crustaceans) is relatively high [8]. Other diverse and "visually advanced" animal groups like chordates and mollusks have three or four eye types, respectively, but pancrustaceans exhibit seven of the eight major optical designs found in animals [8]. In is important to clarify that our use of 'disparity' in pancrustacean eyes does not have a direct relationship to evolutionary history (homology). For example, although related species often share optical designs by homology, optical design can also change during evolution in homologous structures. Insect stemmata share homology with compound eyes, but have a simplified optical design compared to compound eyes [9]. We argue that because of the range of eye designs, pancrustaceans are a key group for examining molecular evolutionary history in the context of morphological disparity.

### Targeted gene families involved in eye development

Despite visual disparity within insects and crustaceans, morphological and molecular data suggest that many of the developmental events that pattern eyes are shared among the Pancrustacea. For example, several key morphological events in compound eye development are conserved, suggesting that this process is homologous among pancrustaceans [10-18]. While the genetics of eye development are unknown for many pancrustaceans, we rely on comparisons between *Drosophila *and other insects. For instance, there are several genes commonly expressed in the *Drosophila *compound eye, stemmata and Bolwig's organ patterning [rev. in [19]] that are similarly employed in eye development in other pancrustaceans [e.g. [9,11,20-24]].

In our analyses, we examine developmental gene families falling into three classes: 1) Gene families used early in visual system specification: Decapentaplegic (Dpp), Engrailed (En), Hedgehog (Hh), Kruppel (Kr), Wingless/Wnt1 (Wnt1), and Zerknullt (Zen). 2) Gene families used in retinal determination and patterning: Dachshund (Dac), Eyes absent (Eya), Eyegone/Twin of Eyegone (Eyg/Toe), Pax-6, and Sine Oculis/Six1/2 (Six1/2). 3) Gene families used in photoreceptor differentiation: Epidermal Growth Factor Receptor (Egfr), Glass (Gl), Munster (Mu), Notch, Spam, Spitz (Spi), and CVC homeobox (Vsx). While most of the gene families among these three classes have only been examined extensively in *Drosophila*, studies in other arthropods suggest at least some developmental conservation [e.g. [11,20-25]]. Interestingly, several of these genes are also involved in vertebrate eye specification, suggesting possible ancestral bilaterian eye-specification gene families [26]. However, most of these gene families are used in multiple developmental contexts, making ancestral assignments impossible without more data. For this reason, we focus on the evolutionary history of the genes themselves, rather than ancestral function. Although other genes are known to be involved in these processes, we focus on gene families with known functional interactions to maintain a narrow scope that allows us to test our hypotheses. Other more extensive datasets could be used in future similar analyses as new information on eye-gene function accumulates.

### Targeted gene families involved in phototransduction

Phototransduction is the pathway for photosensitivity and visual processes as it converts a light signal from the environment to an electrical signal in photoreceptor cells. The pathway is regulated by photosensitive proteins in the opsin family. In animals, two major opsin clades (R- and C-opsins), and their associated pathway proteins, tend to be segregated between rhabdomeric and ciliary photoreceptor cell types [27-30]. R-opsins are thought to have originated prior to the bilaterian ancestor [29]. Where the pathway is known, pancrustacean vision is dependent on the rhabdomeric phototransduction pathway [31,32]. This begins with R-opsin and proceeds through a G-protein signaling cascade wherein the Gq-alpha subunit interacts with Phospholipase C (PLC), causing Transient Receptor Potential (TRP) ion channels to open for membrane depoloarization [33]. Arrestin (Arr) attenuates the signal by inhibiting further G-protein activation [34,35].

### Evolution of complexity

Our first hypothesis, that genomic complexity facilitates morphological complexity, predicts a pattern of high gene duplication rate in an organismal clade with high optical disparity. Our second hypothesis, that functional relationships constrain the histories of individual genes, predicts that gene families with direct functional interactions or common regulation (e.g. retinal determination genes) will show similar patterns of gene loss and duplication. This second hypothesis addresses the question of the evolution of gene regulatory networks. In the context of gene duplication, there are two non-mutually exclusive ways that gene regulatory networks originate. The first is via co-option, where new regulatory interactions are formed between a duplicated gene and other genes. The second is via co-duplication, wherein all genes in a highly-conserved module (such as a regulatory network) duplicate in the same phylogenetic interval and continue to interact within diverging daughter clades. When genes are involved in a highly conserved module and used in various contexts, we might expect that changes to specific genes in the module via duplication and divergence would be mirrored in changes to the other components. That is, if two genes act in a conserved manner over evolutionary time, then the retention of a duplicate of one gene might result in a greater chance of retention for the duplicate of the other gene. One prominent example of co-duplication of network genes preceding the evolution of greater visual complexity is the origin of vertebrate rod and cone specific photoreceptor gene networks [7,36,37]. Similar situations can also be envisaged for co-loss. In the current study, we look at duplication and loss patterns across a large genetic dataset to ask if genes in our dataset tend to duplicate and be lost in tandem, showing patterns of co-duplication/loss.

## Results

**Figure 1:**
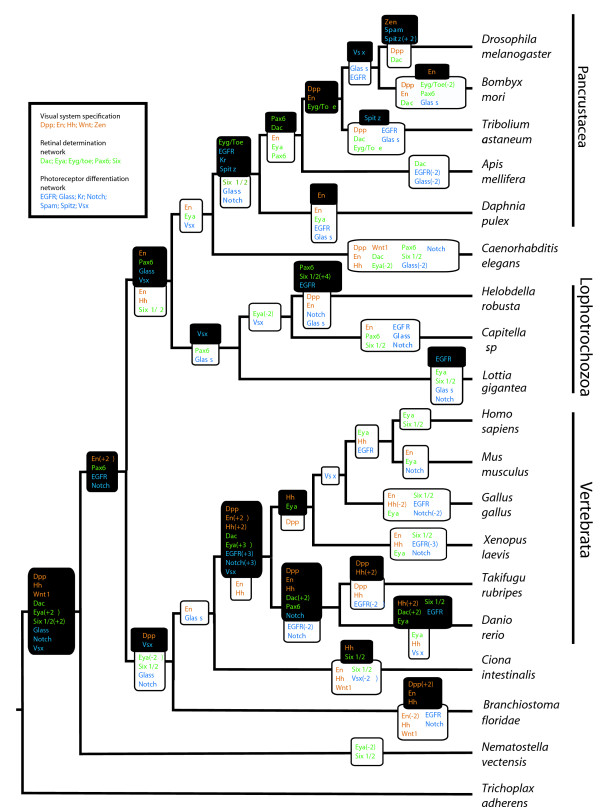
**Duplication and loss of developmental gene-family members in our dataset**. Duplications (bold, black background) and losses (italics, white background) were mapped onto a consensus species tree [79,104,109]. Multiple duplications or losses in a phylogenetic interval are indicated in parentheses. Gene names are color coded by their function in *Drosophila *eye development. Reconciliation of gene trees onto the species tree was performed with Notung using Maximum Likelihood gene family trees (see Methods).

**Figure 2:**
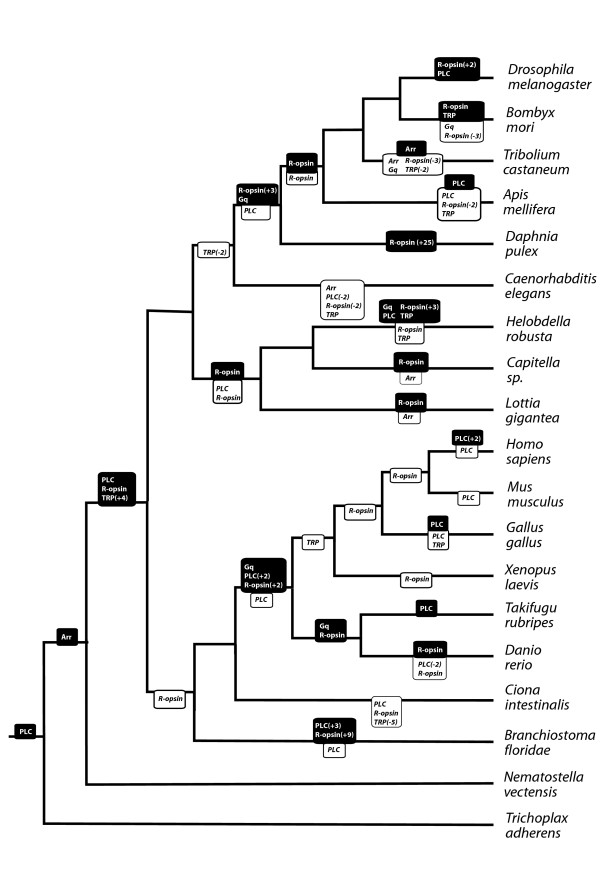
**Duplication and loss of phototransduction gene-family members in our dataset**. Duplications (bold, black background) and losses (italics, white background) were mapped onto a consensus species tree [79,104,109]. Reconciliation of gene trees onto the species tree was performed with Notung using Maximum Likelihood gene family trees (see Methods).

**Figure 3:**
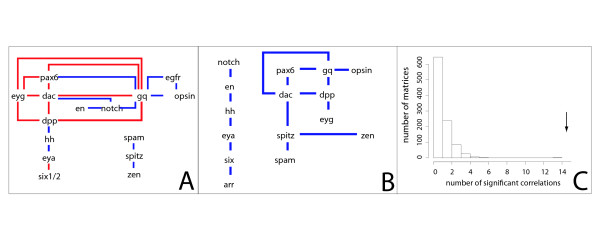
**Gene families with correlated patterns of duplication/loss**. Loss and gain patterns over 36 phylogenetic intervals (Figures 2 and 3) were compared between each pair of gene families. **A) **Gene families with significant correlation of their duplication patterns (see Methods) are connected with a blue line. Gene families with significant correlation of their loss patterns are connected with a red line. **B) **To partially account for the probable high amount of functional overlap between members of the same gene family, we calculated overall gene family duplication/loss activity over a phylogenetic interval (number of losses subtracted from number of duplications) and compared patterns between gene families over all phylogenetic intervals. Pairs with a significant amount of correlation (see Methods) are connected by blue lines. 15 genes had significant correlation with at least one other gene family. This is much higher than the number found in our randomly shuffled matrices (C, see Methods). **C) **Histogram representing the numbers of correlations found in 1000 randomly shuffled matrices using the gain minus loss data (see Methods). Most shuffled matrices had 0 or 1 significant correlations, representing 0 or 2 genes, a few had 5 or 6. Arrow represents the number of significant correlations (14) found in the unshuffled gain minus loss data matrix.

The sequencing of the *Daphnia pulex *genome allows us, for the first time, to infer genomic-level arthropod evolution beyond the insect clade. Within the *D. pulex *genome, we identified any homologs of 23 gene families involved in eye development and phototransduction (based on those in *Drosophila*). We then constructed gene-trees (Additional File 1) for each of these families based on protein sequence from 19 taxa with completed genomes (Tables 1 and 2, Additional File 1) and reconciled the gene trees to an assumed species tree to infer gene duplication and loss events [38]. These data allowed us to calculate the frequency of homolog loss and gain within each gene family across phylogenetic intervals on our assumed species tree (Figures 1 and 2). We found that for certain gene families there was a significantly higher rate of duplication in the pancrustacean clade compared to other clades of animals. With these inferred patterns of gene loss and gain, we performed correlative analyses to identify co-duplication of gene families. While we found that several gene families exhibit co-duplication/loss with at least one other gene, in many cases these correlations are between genes that are without a known functional relationship (Figure 3, Additional File 2).

**Table 1:** Genomes used in our analyses

Organism	Reference	Protein Database
*Apis mellifera *(bee)	[85]	protein (8/7/2006), NCBI
*Bombyx mori *(silkworm)	[86]	GLEAN merged consensus, silkworm genome consortium
*Branchiostoma floridae *(lancelet)	[87]	annotated proteins v1
*Capitella spI *(capitella)	[JGI, unpublished data]	filtered models v1, JGI
*Caenorhabditis elegans *(roundworm)	[88]	WS180.49 peptides, wormbase
*Ciona intestinalis *(tunicate)	[89]	filtered models v1, JGI
*Danio rerio *(zebrafish)	NCBI	protein (6/11/2008)
*Daphnia pulex *(waterflea)	[JGI, unpublished data]	filtered models v1.1, JGI
*Drosophila melanogaster *(fruit fly)	NCBI	BDGP5.4.49 peptides
*Gallus gallus *(chick)	NCBI	protein (11/28/2006)
*Helobdella robusta *(leech)	[JGI, unpublished data]	filtered models v3, JGI
*Lottia gigantea *(snail)	[JGI, unpublished data]	filtered models v1, JGI
*Monosiga brevicollis *(monosiga)	[90]	filtered models v1, JGI
*Mus musculus *(mouse)	NCBI	annotated proteins v3,
*Nematostella vectensis *(anemone)	[91]	filtered models v1, JGI
*Takifugu rubripes *(pufferfish)	[JGI, unpublished data [92]]	filtered models v4, JGI
*Tribolium castaneum *(beetle)	NCBI	protein (6/5/2008)
*Trichoplax adhaerens *(trichoplax)	JGI	filtered models v1, JGI
*Xenopus tropicalis *(frog)	JGI	filtered models v4, JGI

**Table 2:** Summary of gene-family phylogenies

Gene family	*D. pulex *genes	Protein model name(s)	Scaffold # Location	previous trees	expansion in pancrustaceans?
***visual system specification gene families***					
Decapentaplegic (Dpp)	1	*Dappu-347232*	2:2889112-2890686	[93,94]	No
Engrailed (En)	2	*Dappu-290630*	106:41493-47422	--	Yes
		*Dappu-290638*	106:25280-34404		
Hedgehog (Hh)	2	*Dappu-347555*	207:81902-105568	[95]	No
Wnt1	1	*Dappu-44743*	8:1160125-1175065	[96,97]	No
Zerknullt (Zen)	0			[98]	Yes, fly
***retinal determination network gene families***					
Dachshund (Dac)	1	*Dappu- 310049*	1:4072756-4116888	[99]	Yes, insects
Eyes-absent (Eya)	1	*Dappu- 204955*	1:62314-73955	[100]	No
Eyegone (Eyg/Toe)	1	*Dappu- 253988*	74:25897-37400	--	Yes
Pax-6	2	*Dappu- 249978*	51:368439-379409	[101,102]	Yes
		*Dappu-249991*	51:427984-441429		
Six 1/2	1	*Dappu-65962*	275:49584-50586	[102]	No
***photoreceptor differentiation gene families***					
Epidermal Growth Factor	1	*Dappu-324147*	60:112587-119018	[103]	Yes
Receptor (EGFR)		*Dappu- 321139*	39:412588-415432		
Kruppel (Kr)	1	*Dappu-290527*	2:2312128-2315494	--	No
Glass (Gl)	1	*Dappu-234903*	4:2598047-2600296	[39]	No
Munster (Mu)**	0			--	?
Notch	1	*Dappu-328760*	108:325324-337022	[90,104]	No
Spam***	0			---	--
Spitz (Spi)	1	*Dappu-271304*	1714:5914-7575	[103]	Yes
CVC Homeobox (Vsx)	1	*Dappu-323346*	53:603419-625383	[105]	Yes, silkworm+fly
***phototransduction gene families***					
Arrestin (Arr)	2	*Dappu-216585*	86:316599-318172	[106]	Yes, beetle
		*Dappu-207575*	5:2476074-2477692		
Gq-alpha	2	*Dappu-211929*	25:514047-515490	[107]	No
		*Dappu-188187*	25:531825-536252		
R-opsin	30	see Colbourne J et al: Genome Biology of the Model Crustacean *Daphnia pulex*, submitted		[29]	Yes
Phospholipase-C (PLC)	2	*Dappu- 226357*	53:369165-377304	[108]	Yes, fly, bee
		*Dappu- 304714*	3:1803843-1812297		
Transient Receptor	2	*Dappu-54362*	41:27419-33467	--	Yes
Potential Channel (TRPC)		*Dappu- 309057*	9:569391-574613		
		*Dappu- 309057*	56:282882-311121		

**No genome-scale tree.

***Only a single member with this domain architecture found.

### Comparison to previously hypothesized gene trees

After searching whole genome sequences (see Methods), we estimated gene trees for 22 different gene families (Additional File 1, Table 2). We were unable to estimate a tree for Munster due to ambiguous homology with other genes. For many of these gene families, this was the first phylogenetic analysis utilizing searches of whole-genome data. For several gene families this was also the first pan-Metazoa phylogenetic analysis (Table 2). By searching whole-genome data, we were able to find new members of several gene families. For example, we were able to generate a more complete phylogenetic hypothesis for the Glass (Gl) gene family with the discovery of a new chordate homolog. While a previous unpublished phylogeny identified an echinoderm Gl homolog [39], no homologs have been reported previously in chordates. Through the analysis of the chordate *Branchistoma floridae *genome, we discovered a putative homolog which groups with the other Gl genes with very high support (aLRT = 1) and has similar domain structure to other known Gl genes. This suggests a loss of the Gl gene family of zinc-finger members early in chordate evolution (Additional File 1, Figure S13). In addition, our analyses uncovered one gene from zebrafish and one from fugu that together form a sister group to all other Dpp/BMP2/4 genes. This relationship implies a paralog present in fugu and zebrafish but absent in all other eumetazoans. These fish homologs have not, to our knowledge, been studied, except in having been named BMP2 based on similarity searches associated with zebrafish and fugu genome projects, so confirming the presence of new functional Dpp/BMP2/4 genes in these fish would require experimental demonstration.

We examined Pax-6 in greater detail than other gene families by including sequences from additional arthropod species. This gene has a well-known and conserved role in eye development, and previous authors have indicated, counter to our conclusions, that the genes *eyeless (ey) *and *twin-of-eyeless *(*toy*) are insect- specific duplications [40]. We found strong phylogenetic evidence for a pre-arthropod duplication of *ey *and *toy*. We found support for a family of genes that includes *Drosophila toy*, a myriapod *toy*-like gene, plus a newly described *toy*-like gene from an ostracod crustacean and a chelicerate. The *toy*-clade excludes *Drosophila ey *and the *ey*-like genes of a crustacean and a myriapod. We conclude it is very unlikely that *toy *and *ey *represent an insect-specific duplication event, although the precise timing of this duplication is difficult to determine with currently available data.

### Pancrustaceans have high rates of gene-duplication within our dataset

While excluding arthropod-specific gene families (Spitz, Spam, and Zen), we analyzed and compared rates of gain of gene-family members (duplications) across pancrustaceans, across non-arthropod protostomes (Lophotrochozoa and *Caenorhabditis elegans*), and across vertebrates. We used three denominators to calculate rates of gene duplication (ie rate equals distance/time, and we used three different metrics of evolutionary 'time' to calculate gene duplications/time). Using total gene duplications in the denominator normalizes by overall rates of gene duplication in each clade, which includes any whole genome duplications that occurred in a particular group. A second denominator was genetic distance, utilizing average ortholog divergence between species in a clade [41]. Genetic distance normalizes by the overall molecular diversity in a clade. Our third denominator was a molecular clock estimate of divergence times [42,43]. Compared with other protostomes, we found that duplication rates of eye-genes were significantly higher in pancrustaceans in all three analyses (see Methods). Compared with vertebrates, eye-genes showed higher duplication rates in pancrustaceans when normalized by total gene duplications. However, comparing duplication over both molecular clock divergence times and genetic distance yielded similar rates of eye-gene gain in vertebrates and pancrustaceans.

In our first analytical measure of duplication rates, we normalized the number of duplications observed in our eye-gene dataset by the total number of gene duplications calculated from the genomes of the clade of interest. We inferred 50 duplications of eye-related genes in pancrustaceans compared to 33113 total duplications in the pancrustacean genomes, resulting in a ratio (δ) of 0.0015 (Table 3). This is significantly higher than the δ value for non-arthropod protostomes (δ = 0.00026; Fisher's exact test, *p *= 1.5e-11) or vertebrates, (δ = 0.00058; *p *= 4.9e-6) (Tables 3 and 4). To further scrutinize duplication rates, we examined developmental and phototransduction genes separately. The difference between the δ of non-arthropod invertebrates and pancrustaceans was still significant for both developmental (*p *= 0.0102) and phototransduction (*p *= 1.47e-10) genes. When compared to vertebrates, only the δ for phototransduction genes, and not developmental genes, was significantly higher in pancrustaceans (*p *= 2.52e-11) (Tables 3 and 4).

**Table 3:** Gene duplication rates

clade(s)	Dataset gene duplication rates								
	Eye duplications/ total duplications (δ)			Eye duplications/ genetic distance (ι)			Eye duplications/ molecular clock (μ)		
	**All**	Dev*	PT*	**All**	**Dev**	**PT**	**All**	**Dev**	**PT**
pancrustacean	.0015	3.9e-4	.0011	.0478	.0124	.0353	.1064	.0277	.0787
other protostomes	2.6e-4	1.2e-4	1.4e-4	.0193	.0091	.0102	.0215	.0101	.0114
vertebrate	5.8e-4	4.3e-4	1.5e-4	.0577	.0430	.0184	.1044	.0778	.0267

*Developmental genes (Dev) and Phototransduction genes (PT)

**Table 4:** Duplication rates in Pancrustacea compared to other clades

clade(s) compared to Pancrustacea	p-values for significant difference in dataset gene duplication rates compared to Pancrustacea								
	Eye duplications/total duplications (δ)			Eye duplications/ genetic distance (ι)			Eye duplications/ molecular clock (μ)		
	**All**	**Dev**	**PT**	**All**	**Dev**	**PT**	**All**	**Dev**	**PT**

Other protostomes	**1.5e-11**	.**0102**	**1.47e-10**	.**0010**	.5180	.**0004**	**1.9e-9**	.**0381**	**8.2e-9**
vertebrate	**4.9e-6**	.8741	**2.52e-11**	.4015	*8.79e-5*	.**0080**	1.00	.*0016*	**.0010**

Bold = significantly more duplications in pancrustaceans

Italics = significantly more duplications in non-arthropod clade

We also used genetic distance (average number of amino acid substitutions between orthologs in a clade) as a second measure of evolutionary rate [41]. This measure allows us to calculate gene duplications per amino acid substitution (ι) to examine gene duplication in the context of overall lineage diversity (Table 3). For pancrustaceans, we found that ι for eye genes was 0.0478, significantly higher than ι for non-arthropod protostomes (ι = 0.0193, *p *= 0.0010). However, ι was higher in vertebrates (ι = 0.0577) than pancrustaceans. We also calculated ι separately for developmental and phototransduction genes. Pancrustacean ι (0.0124) and non-arthropod protostomes ι (0.0091) did not differ significantly for developmental genes, although vertebrate ι was significantly greater (ι = 0.043, *p *= 8.79e-5). For phototransduction genes, pancrustacean ι (0.0353) was significantly higher than ι for non-arthropod protostomes ι = 0.0102; *p *= 0.0004), and significantly higher than ι for vertebrates ι = 0.0184, *p *= 0.0080) (Tables 3 and 4).

Finally, we used a calibrated molecular clock as a third measure of evolutionary time. One critique of ages inferred by molecular clock studies is that they often overestimate absolute clade ages [44-48]. Even so, the estimates could still be reliable estimators of *relative *clade age, which is what we require for comparing rates in different clades. Utilizing published molecular clock-based divergence time estimates [42,43], we found results very similar to our analysis using genetic distance. Overall, eye-gene duplication rates standardized using clock divergence time estimates (μ) were found to be significantly higher in pancrustaceans (0.1604) than other protostomes (0.0215, *p *= 1.9e-9) but were not significantly different than μ for vertebrates (0.1044). Although developmental genes analyzed alone were not significantly different between pancrustaceans and vertebrates, phototransduction genes showed a significantly higher μ in pancrustaceans compared to vertebrates (*p *= 0.0010). Both sets of eye-genes showed a significantly higher μ compared to other protostomes (Tables 3 and 4).

In all three analyses, eye genes showed a higher rate of duplication in pancrustaceans than in non-arthropod protostomes. In contrast, pancrustaceans only show higher rates of duplication than vertebrates when phototransduction genes are included in the analysis. That is, pancrustaceans do not show higher rates of developmental gene duplication compared to vertebrates under any analysis.

### Co-duplication is significant in our dataset

We compared gene losses and gene duplications separately across Metazoan genomes and found that 15 of 22 gene families had correlated patterns of loss or gain with at least one other gene family (Figure 3a). In a separate analysis, we compared patterns of gene loss and duplication simultaneously by taking the total number of duplications minus losses for each gene family over each branch of the species tree. Both analyses yielded similar results (Figure 3b).

To test the statistical significance of our observed number of correlations, we randomized our data matrix by shuffling to generate a null distribution of correlations. We found that the majority of shuffled matrices (1000 total) had only either 0 or 1 significant correlations between gene families and none had over 6 correlations, making our observed result of 15 significantly different from the null distribution (Figure 3c). This confirmed that the number of correlated gene families in the real dataset is greater than expected under by chance. However, the genes showing high levels of correlation in gain/loss patterns were not primarily genes with known functional relationships (Figure 3, Additional File 3).

## Discussion

Here we have examined the evolutionary history of genes involved in eye development and phototransduction by analysis of gene family trees, reconciled trees, and co-duplication data. Thus, we examined gene-family evolution in the context of both morphological disparity (eye disparity vs. gene duplication rates) and protein network evolution (co-duplication/loss). In most of our analyses, we found that increased rates of duplication within specific eye-gene families were correlated with the increased optical disparity seen in pancrustaceans. At the protein network level, we found significant co-duplication of eye-genes, though the patterns of duplication are more complex than we originally hypothesized with respect to previously known functional interactions among proteins.

### Gene trees

In our examination of gene duplication and loss, we generated phylogenetic hypotheses for 22 gene families. Many of these are the first detailed analyses of the evolution of the gene family, which will be of use in future research on these gene families in various contexts. We found that gene trees of orthologs often are incongruent with assumed species-level relationships. Assuming our inferred gene trees are accurate, our results imply that there is a more complex pattern of gain and loss than would have been expected by simply comparing number of gene orthologs in each species. Because errors in our trees could lead to overestimations of complexity, we required support values of 90% or higher in our reconciliation analysis. That is, we allowed branch swapping to minimize duplications and losses in cases where the node support was less than 0.9. However, this method can underestimate the numbers of gains and losses [49]. Future inquiries could focus on these poorly supported nodes, by including additional species in the analyses as they become available, or by including additional information (e.g. domain structure or intron presence/absence) in attempts to estimate phylogenies with higher support. In addition, presumably more accurate reconciled trees could be generated in the future using more computationally expensive methods, such as fully Bayesian estimation of gene and species reconciliation [50,51], which currently would be very difficult at the scale of analysis conducted here. The inclusion of additional genomes in future studies will also be of help in generating accurate hypotheses, as taxon (in this case gene) sampling is an important determinant of phylogenetic estimation [52].

### Rates of gene duplication

We found overall support for the hypothesis that gene duplication and/or retention rates are higher in pancrustaceans, the group with the highest disparity of optical-types. We examined the sensitivity of this overall conclusion in three different ways. First, we compared pancrustaceans to both non-arthropod protostomes and to vertebrates. Second, for each of these comparisons, we estimated gene duplication rates using three different denominators: total gene duplications, overall genetic distance, and divergence time estimates from molecular clock analyses. These different denominators are necessary to understand the influence of different modes of genome evolution on our conclusions, such as the multiple genome duplications known in vertebrates. Third, we examined (both separately and together) duplication rates of genes from different eye-gene categories (developmental versus phototransduction genes), allowing us to test whether one category was the primary driver of the overall rates. For example, developmental genes are probably involved in more non-visual phenotypes than phototransduction genes since phototransduction genes often have localized expression [e.g. [53]], and this difference in pleiotropy could influence final results.

Comparisons between eye-gene duplication rate in pancrustaceans and non-arthropod protostomes clearly supported our hypothesis, even when taking the conservative approach of not counting arthropod-specific genes. The observed difference in gene duplication rate between these two clades does not depend on the denominator used in rate calculations, and is significantly different for both developmental and phototransduction genes (Tables 3, 4). Despite the consistency of these results, it is important to consider that there are multiple possible causes for our observed correlation between higher optical disparity and higher eye-gene duplication rate. One possible explanation is that gene duplications, perhaps retained by natural selection, are a causal factor in increasing optical disparity in pancrustaceans. In fact, gene duplications are known to have increased retinal complexity in vertebrates, leading to separate rod and cone phototransduction pathways [7,36,37]. Whether these vertebrate duplications were fixed by natural selection or neutral processes is unknown. At present, however, too little is known about the relationship between pancrustacean genes and optical design phenotypes to claim that gene duplication was a causal factor leading to higher optical disparity. Another explanation is that the available full genome sequences do not allow for appropriate estimates of duplication rates in these clades. For example *C. elegans *does not possess conventional eyes, even though many other non-arthropod protostomes do. If, as a result of losing eyes during evolution, the lineage of *C. elegans *has a lower rate of eye-gene duplication, this could result in an underestimate of eye-gene duplication rate for the entire clade. Similarly, the pancrustaceans used here could have more eye-genes than other arthropods. In fact, *Daphnia pulex *does have a large number of genes compared to other arthropods, perhaps because of its asexual/sexual life history (Colbourne J et al: Genome Biology of the Model Crustacean *Daphnia pulex*, submitted). These hypotheses could be examined using the approaches developed here, once additional genome sequences become available.

Compared to rate differences between pancrustaceans and non-arthropod protostomes, rate differences between pancrustaceans and vertebrates were more variable. That is, using different denominators in our rate calculations led to different results (total gene duplications, genetic distance, or molecular clock). An important consideration in these comparisons is that vertebrates are known to have undergone multiple whole-genome duplications, which raised the overall estimated rate of gene duplication and accumulation for the group. This is evident in total gene duplications that we counted (80673 in vertebrates vs. 33113 in pancrustaceans) but is not reflected in our other distance measures (denominators): both clades show similar genetic distance (as measured by average ortholog distance - 1047 and 814 respectively) as well as similar clade ages (as estimated by a molecular clock - 470 and 450 mya). The high overall rate of gene duplication and accumulation in vertebrates may therefore explain why, counter to our hypothesis, vertebrates show a significantly higher rate of eye development gene duplication than pancrustaceans.

The high rate of duplication and/or retention of genes in vertebrates further suggest that the best rate comparison might be that using total number of gene duplications as the distance between species (denominator). It is this rate calculation that corrects for vertebrate whole-genome duplications. Even here, we see a difference between gene types, with only phototransduction genes, and not developmental genes, supporting our starting hypothesis that pancrustaceans have a higher eye-gene duplication rate. However, much of the significant difference in phototransduction genes is driven by extensive duplications of opsin in the *D. pulex *lineage (Colbourne J et al: Genome Biology of the Model Crustacean *Daphnia pulex*, submitted), a phenomenon also known in other crustaceans [54,55]. Given the observed difference between developmental and phototransduction genes when comparing vertebrates and pancrustaceans, it is tempting to speculate on possible biological causes for this result. For example, we expect developmental genes to be pleiotropic, and several of the genes studied here are known to function in many contexts besides eye development [e.g. [56]]. Phototransduction genes have a more specific functional role and may be less pleiotropic [e.g. [53]]. The more pleiotropic developmental genes could rely more heavily on modifications in the protein and cis-regulatory sequences, rather than on gene duplication for diversifying function [57]. If so, correlation between gene duplication rate and morphological disparity could be low or nonexistent.

The consideration of pleiotropy also highlights another avenue for future research. If pleiotropy does result in a weaker correlation between eye disparity and gene duplication rate, gene choice must influence the final results. Therefore, future research might focus on a broader sampling of genes, especially to the extent that analyses conducted here could be fully automated to allow the analysis of very large datasets. For example, a recent study focusing on the insects found higher numbers of gene duplications in dipterans than other insects by examining 91 fly eye-genes [58]. Integrating this type of "retinome" scale analysis with the methods we show here would give a more detailed and informed view of gene evolution in the context of morphological disparity and innovation.

The available genomic data allowed us to test the hypothesis that pancrustaceans, a group with many disparate eye types, have more duplications of eye-genes than less optically-diverse groups. This relies on an assumed species phylogeny, and our assumption that we are estimating rates of pancrustacean duplication for the entire clade. Complicating this assumption, the phylogenetic position of branchiopods (including *Daphnia pulex*) within Arthropoda remains somewhat uncertain [59-62]. We here consider the hexapod/*D. pulex *ancestor to be the common ancestor of all pancrustaceans for simplicity. This is justified by the wide variety of optical designs found in this hypothesized hexapod-branchiopod clade, regardless of whether it represents the ancestral pancrustacean or whether crustaceans are in fact paraphyletic [59-62]. Future research using genomes from more crustaceans and taxa with a wider range of eye-type disparity could allow testing for a broader correlation between eye disparity and eye-related gene number, a possibility supported by our results. Namely, if the ratio of eye-types to gene duplication rate is similar in different clades, then a broader correlation may exist.

### Co-duplication of genes

We found that duplication and/or loss patterns in 15 of 22 gene families correlated significantly with duplication and/or loss patterns in at least one other gene family, significantly more than expected by chance (Figure 3C). Interestingly, many of the genes we found to co-duplicate are not known to have any functional relationship with each other. This suggests the possibility of novel functional relationships between genes, at least in animals where the genetics are relatively unstudied (the majority of our samples). Co-duplications may also be the result of undiscovered constraints at the genomic level (e.g. synteny), or an unknown systematic artifact of our gene reconciliation analysis that infers that unrelated genes duplicate or are lost at particular nodes. While new gene pairings were suggested by our co-duplication analysis, some pairings predicted by functional modules were not found.

One functional module of particular interest is the suite of phototransduction genes [31]. We found that even though multiple ciliary phototransduction genes are known to have co-duplicated early in vertebrate history [29,36,63], rhabdomeric phototransduction genes have not co-duplicated as a unit when considering the entire history of Metazoa. A notable exception is that R-opsin and Gq-alpha (genes known to interact directly) exhibit a significant pattern of co-duplication. This suggests that R-opsin and Gq-alpha have been a tightly linked functional module throughout animal evolution, and if so, specific R-opsin paralogs may be expressed with specific Gq-alpha paralogs.

We also found that some phototransduction genes co-duplicate with developmental genes (Figure 3). Some of our data could represent novel genetic interactions, but they could also stem from other unknown aspects of these genes including the number of protein interactions, the number of functions a protein is involved in, or genomic location. Although we tested the general false-positive rate by generating randomized matrices of our data, future studies might also compare the numbers of co-duplicating eye-genes to that of a set of genes drawn at random that are not necessarily involved in the same organ system.

Similarly, we found extensive co-duplication/loss between only a few gene families known to be involved in the same developmental pathway [19]. The retinal determination pathway, for example, includes Pax6, Dac, Eya and Six1/2, gene families known to have functional interactions in disparate taxa [56]. From this pathway, Pax6 and Dac had correlated loss patterns as did Eya and Six1/2. Perhaps the functional relationship between these gene pairs is more constrained than that of other genes in the retinal determination network. Dac and Pax6, for instance, are known to have a complex inductive relationship in both vertebrates and invertebrates [56,64]. Other gene families with known interactions in *Drosophila *compound eye development also showed correlations in either their loss or gain patterns. These include Hh and Eya [65], Dac and Dpp [66] and Six 1/2 and Eya [67]. However, the majority of genes with known regulatory interactions in eye development did not tend to be duplicated/lost together more often than expected by chance. This finding - that the evolutionary history of genes belonging to complete genetic modules do not share similar patterns of gain and loss - is consistent with a functional study that found network degeneration after genome duplication in yeast [68]. In that study, genes that function together before genome duplication do not necessarily function together after genome duplication.

## Conclusion

Our research provides new methodology for examining genomic complexity in the context of morphological complexity. In particular, we examined the evolutionary histories of genes acting in arthropod eye development and phototransduction to evaluate hypotheses of gene and protein module duplication. The phylogenetic trees we created lay a foundation for research into the gene histories of several understudied, but developmentally important, gene families. Future research will likely lead to advances in understanding evolutionarily conserved protein domains in these genes as well as the significance of the expansion of some families in particular lineages (e.g. the Six1/2 family in the lineage leading to the *Helobdella robusta*). Our analyses of these gene histories revealed that, by one analysis, genes involved in eye development and phototransduction had higher rates of duplication in the taxon with the largest number of eye types (pancrustaceans) (Table 3). Our co-duplication analysis found higher than expected numbers of co-duplicating genes, yet genes in known genetic modules were not always found to be gained and lost together (Figure 3). Moreover, some genes that are not known to have extensive interactions did show high correlation in loss and gain pattern. Future research could clarify these findings, comparing the genomic locations of co-duplicating genes in order to identify synteny, identifying gene modules in the eyes of non-model organisms, confirming the function of the gene families in non-insect arthropods, and testing for patterns consistent with positive selection acting on the genes and modules of interest.

## Methods

### Overview

We first found all homologs of genes of interest in the *Daphnia pulex *v1.0 genome. We next found all homologs in 18 other metazoan genomes. We constructed phylogenies for each gene family using maximum likelihood. Assuming species-level relationships to be known, we next reconciled each gene family tree with the metazoan tree to estimate timing of gene duplication and loss events. We then estimated rates of gene duplication within major metazoan clades. Finally, we tested for significant correlation of gene duplication/loss patterns across gene families. Detailed methods for each of these general steps are detailed below.

### *Daphnia pulex *genome searches and gene family assignment

With a protein sequence for each gene of interest from FlyBase used as a "bait" sequence, *Blastall *searches were performed, using protein sequences for each gene of interest as a "bait" sequence, against all gene models of *Daphnia pulex *v1.0 obtained from JGI [http://genome.jgi-psf.org/Daphnia; http://wfleabase.org/]. Searches first retrieved the top 15 hits, this number was raised in subsequent searches until *D. pulex *models outside the group of interest were obtained. Redundant sequences were determined by examining the visual scaffold model on JGI and then removed by hand. The gene family for each *D. pulex *gene was assigned by inclusion in a maximum likelihood tree using UniRef50 and UniRef90 sequences. These trees were estimated using an in-house pipeline of shell and perl scripts that merge existing bioinformatic tools. The bait sequence from FlyBase was used to perform a similarity search using blastp [69] of non-redundant protein databases curated by uniprot http://www.uniprot.org/. In most cases, we used two blast search strategies for each bait gene: 25/10 (where the top 25 blast hits of the Uniref90 and the top 10 blast hits of the Uniref50 database were retained for further analysis). In cases when there was either not enough resolution or no outgroup hits obtained; more hits were taken from the Uniref90 or Uniref50 databases, respectively (See Additional file 1 for details). Identical sequences, such as those obtained from both Uniref90 and Uniref50 databases, were removed from further analysis. Second, all retained sequences and bait were aligned using MUSCLE [70]. Third, we estimated maximum likelihood phylogenetic trees using aLRT-PHYML [71,72] assuming a JTT [73] model of protein evolution. We visualized resulting phylogenetic trees with TreeView [74,75] or FigTree http://tree.bio.ed.ac.uk/software/figtree/. Where relevant, we tested whether gene trees were significantly different from previous trees using the Shimodaira-Hasegawa (SH) test [76] implemented in PhyML [71,72] by comparing constrained trees to the best trees.

### Pax-6 sequences

In phylogenetic analyses of Pax-6, we utilized previously unpublished sequence data from *Daphnia pulex *(confirming the automated genome assemblies with cDNA sequencing) and the ostracod crustacean *Euphilomedes carcharodonta*. *Euphilomedes carcharodonta *were collected at the University of Southern California's Wrigley Marine Lab on Catalina Island, California by free diving, collecting sediment with an aquarium net, and sorting with a dissecting microscope. *Daphnia pulex *were obtained from stock collections at Indiana University. We first isolated Pax-6 fragments using degenerate PCR primers to highly conserved regions in the paired and homeo domains of published Pax-6 sequences. After sequencing an initial Pax-6 fragment, we designed specific primers for 5' and 3' RACE, often using nested primers and the Gene Racer kit (Invitrogen). Primers and cycling conditions are given in Additional File 3. Additional arthropod Pax-6 sequences were obtained from GenBank.

### Genome comparisons

With protein sequence for each gene of interest from FlyBase, initial *blastall *searches were executed against 19 genomes obtained from JGI and NCBI (Table 1) with parameters set to return five best hits with e values less than 0.5. To ensure no paralogs were omitted, *blastall *searches were modified and repeated to allow more returns in cases where all five best hits fell within the in-group for the gene of interest in initial phyogenetic analyses. Sequence alignment and maximum likelihood estimates of gene trees were performed as described above. Genes were identified phylogenetically as monophyletic ingroups sharing conserved domain architecture using Pfam databases and NCBI's Conserved Domain Database [77,78] (See Additional File 1 for details). With outgroups identified and pruned from the alignment, each gene tree was reconstructed and then reconciled to the metazoan species tree [79] using NOTUNG [80]. Rearrangements were allowed for node supports below 0.9 in order to minimize the inferred number of gene duplication and loss events implied by poorly supported nodes.

### Duplication pattern analysis

We estimated rates of gene duplication while normalizing for overall evolutionary time using three different denominators. Total numbers of duplications of genes along each branch of a species tree were estimated using data from the reconciled tree analysis (Figures 1, 2). For each phylogenetic group, the numbers of duplications along each branch falling within the group were added together, to get a total number of duplications within a clade. *Caenorhabditis elegans*-specific duplications were added to Lophotrochozoa-specific duplications to get a total duplications value for "non-arthropod protostomes." *Ciona intestinalis, Branchiostoma floridae*, and *Nematostella vectensis *were not included in this analysis. To estimate total numbers of gene duplications and losses during animal evolution, we used EvolMAP software [41], which estimates the gene content of hypothetical ancestral species relative to evolutionary transitions marked by speciation events. EvolMAP uses an assumed species tree, performs pair-wise similarity comparisons of all genes, and assumes that gene families cannot be gained independently in separate lineages. Second, we used EvolMAP to calculate average ortholog divergence, here termed "genetic distance", see [41] for details on the calculation. Third, we normalized gene duplication rates using clade age estimates from molecular clock studies [42,43].

**Figure 4:**
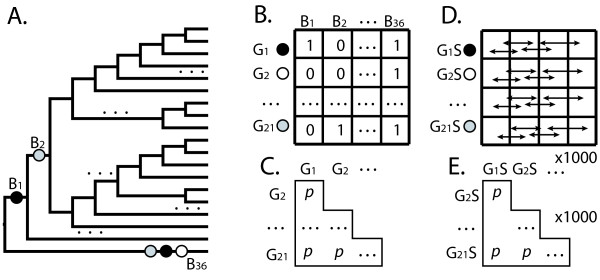
**Test for significant co-duplication of genes**. **A) **Gene Duplications and losses are mapped to the branches (B1 - B36) of the assumed species phylogeny (see Methods for details). Circles represent gene duplication events; different shades represent different gene families. **B) **Gene duplication data illustrated in matrix form. Rows are gene families (G1-G21) columns are branches on the phylogeny (B1-B36). Gene duplication events are represented in the matrix as a 1, absence of gene duplications on branches are represented as 0. **C) **All pair-wise comparisons were made between gene families (rows). P-values were calculated using Spearman's rho [82]. The number of significantly similar gene family pairs is represented in this panel (see Methods). **D) **To test whether the observed number of significantly co-duplicating gene family pairs could be due to chance, we next shuffled each row of the data matrix (G1S-G21S), thus randomizing the gene duplication events on the tree. In this way, we created 1000 shuffled matrices. **E) **Using the shuffled matrices (D), we calculated p-values for the similarity of each pairwise comparison of shuffled matrix rows. The number of significantly similar rows was counted for each of the 1000 shuffled matrices to form a null distribution (Fig 4C), to which the observed value was compared.

To examine co-duplication, two methods were used. First, data matrices were created representing the number of losses or duplications for each gene along each branch of a species tree using the data from reconciled tree analysis (Figures 1, 2). Gene patterns were compared in the loss and gain cases separately by examining correlation using Spearman's rho implemented in R with p-values calculated using Algorithm AS 89, a test of upper tail probability [81,82]. The second method used the same methodology comparing duplication and loss patterns together by looking at the number of duplications minus the number of losses for each gene along each branch of the species tree. Significance of correlation was assessed using sequential Bonferroni to accommodate multiple comparisons [83,84]. A null distribution for the expected number of co-duplicating gene family pairs was created by randomizing the data matrix 1000 times and analyzing each pseudoreplicate in R (Figure 4).

## Authors' contributions

Author contributions: ASR and THO designed research; ASR, MSP, DCP, CV, AES, JMS, ARO, and THO performed research; ARO contributed new reagents/analytic tools; ASR, MSP, DCP, CV, AES, JMS, ARO, and THO analyzed data; and ASR, MSP, DCP, JMS, and THO wrote the paper. All authors read and approved the final manuscript.

## Supplementary Material

Additional file 1 Results of phylogenetic analyses on the 22 individual gene families used in this study. Click here for file

Additional file 2 Pairwise correlation values of between duplication and loss of the 22 gene families examined. Click here for file

Additional file 3 PCR conditions for the amplification of Pax6 homologs from *Euphilomedes carcharodonta *and *Daphnia pulex*. Click here for file
