# Supplementary material for: Gene duplication and the origins of morphological complexity in pancrustacean eyes, a genomic approach
Source: BMC Evol Biol. 2010 Apr 30;10:123. doi: 10.1186/1471-2148-10-123 (PMC2888819; doi:10.1186/1471-2148-10-123)
Supplement: Additional file 1 — Results of phylogenetic analyses on the 22 individual gene families used in this study. [file 1471-2148-10-123-S1.PDF]

Supplementary Figures S1-S22 Legends:
Maximum likelihood trees estimated using **A)** sequences from *D. pulex* genome (red) and UniRef databases using *Drosophila* sequences as search baits in blast similarity searches (see Methods for details of similarity searches and phylogenetic analyses) or **B)** similar sequences (as determined by blast) from 19 full-genome sequences (see Table 1 in text for details). The smallest monophyletic clades containing all known members of each gene-family are shown with yellow backgrounds; duplicate gene-entries (caused by a bait gene retrieving itself from a similarity search of a database, or by databases containing duplicate entries such as splice variants) are colored grey. Outgroup genes (those considered a different gene family) are shown with blue or purple backgrounds.

Early visual system specification
S1: Decapentaplegic (Dpp, called BMP2/4 in vertebrates) is a group within the larger TGFβ-domain containing family and has been found to interact with Hh and Wg in several developmental contexts including eye development [1]. We found a single Dpp in *D. pulex*. Our search of metazoan genomes and phylogenetic analysis concurs with previous reports [2, 3] in showing that Dpp (named BMP2/4 in vertebrates) originated prior to metazoans , as it is found in both *Nematostella* and *T. adherens* but is absent from the choanoflagellate *M. brevicolis*. Based on an SH test [4] our tree is not significantly different in topology from previously published trees [2, 3], when constraining genes found in our searches to relationships indicated by previous studies. Unlike previous studies, our analyses uncovered a gene in zebrafish and fugu that forms a sister group to all other Dpp/BMP2/4 genes. This relationship implies a gene present in fugu and zebrafish was lost in all other eumetazoans. These fish genes have not, to our knowledge, been studied, except in having been named BMP2 based on similarity searches associated with zebrafish and fugu genome projects. According to our analyses, Dpp was duplicated within the insect linage, but one copy was lost in fly, silkworm and beetle. All pancrustaceans have a single Dpp gene, though some of these are orthologues rather than homologues.

S2: Engrailed (En) is a homeodomain protein expressed in a number of developmental contexts including *Drosophila* early eye-imaginal discs [5]. Its function in eye development is currently unknown. Its function in eye development is currently unknown, but likely includes some role in ocellus development [1]. However it should be noted that En is not essential for eye development, and may be playing an at least partially redundant role in an eye-determination network [6]. Our analyses recovered two *D. pulex* homologues of engrailed. Reconciliation analysis suggests that these are the product of a crustacean/branchiopod-specific duplication. Reconciliation analysis also suggests a more complex duplication and loss pattern in the deuterostomes than had been previously hypothesized [e.g. 7].

S3: Hedgehog (Hh) proteins consist of a "hedge" and a "hog" (or intein) domain. They interact with Wnt1 and Dpp proteins in Drosophila eye development to specify the eye primordium [1]. We found a single Hh gene in *D. pulex*. Our genomic analysis recovered two Hh clades – a protostome clade and a deuterostome clade, with *Nematostella* Hh as the sister to the protostome Hh genes. This is slightly different from

a previous analysis, though both trees suffer from weakly supported nodes and have different sampling strategies [8]. Some support for these nodes comes from our reconciliation analysis, which retains this topology. This RTA finds that the only pancrustacean specific expansion of Hh occurred as a duplication in the lineage leading to silkworm, all other pancrustaceans have a single Hh.

S4: Wnt1 proteins (including Drosophila Wg) are members of the Wingless/Int family of genes, which contain one or more Wnt domins. Wnt1 acts in several developmental contexts across animals including eye field specification in *Drosophila* and other pancrustaceans, likely as a repressor of eye differentiation [1, 9-12]. We found a single Wnt1 (Wg) protein in *D. pulex*. Our genomic analysis shows that Wnt1 forms a monophyletic group to the exclusion of other Wnt proteins and includes *Nematostella* Wnt, though this node has very low support. However, this agrees with previous analyses where Wg/Wnt1 proteins have been found to form a monophyletic group, one of these includes cnidarian Wnt genes [13-15]. Our reconciled tree suggests a single origin of Wnt1-class genes at the base of Metazoa with no gains and only two losses – in *Ciona* and *C. elegans*. We find a single Wnt1/Wg gene in all other species.

S5: Zerkneullt (Zen) is a homeobox-containing protein and is a Hox3 family member found only in Diptera. During eye development it acts in conjugation with Dpp and Kr to control the retinal determination genes [16, 17]. Other genes named Zen exist in various arthropods [18, 19], but these do not form a monophyletic group to the exclusion of other Hox3 genes and are not known to act in eye development. We did not recover any Zen genes in *D. pulex*, though we did recover a Hox3 gene. Our reconciliation analysis suggests that Zen evolved in the lineage leading to fly where it is found in two copies and is a good candidate for a gene that might be increasing eye complexity in dipterans.

Retinal determination network

S6: Dachshund (Dac) proteins have a DD1 (or Ski/Sno) and a DD2 domain. In flies, DD1 performs most of the Dac function [20, 21]. Dac genes act in a cassette with other members of the retinal determination network in various contexts across metazoa [reviewed in 22, 23], though its role in eye development in vertebrates is not well understood. In *Drosophila* it is involved in the specification and development of photoreceptors [24] and it may be involved in eye development in other insects [25, 26]. Our phylogenetic analysis using whole translated sequence did not recover a monphyletic Dac clade. This is likely due to high levels of Dac sequence dissimilarity in non-conserved domains. For this reason, we performed a separate analysis using only the DD1 and 2 domain-containing regions, as defined by Tavsanli [20]. In this analysis, Dachshund was still not monophyletic, although every gene falling into our *Drosophila* Dac containing clade at least had the important DD1 in our reconciled tree analysis, including a *Nematostella* gene that lacks DD2 but has sequence similar to DD1. We consider members of this clade to be Dac-class genes. Based on this analysis, our reconciliation analysis suggests that Dac arose in the Eumetazoa with a single copy in all invertebrates surveyed and multiple copies in vertebrates. In the Pancrustacea, Dac duplicated in the insect lineage, but one copy was lost in several lineages such that all insects analyzed have one copy, as does *D. pulex*. While the different paralogs of Dac in

different pancrustacean lineages may have contributed to some of the disparity exhibited in their eyes, it is likely that complexity in the action of Dac, especially with regards to its dual function in eye development, is attributable to an increase in number and/or interactions of its cis-regulatory elements, as suggested by the complex context-specific regulatory interactions of Dac in *D. melanogaster* [17]. Tree in panel B made using only sequence from the D1 and D2 domains. Genes in red have only D1 domain, genes in orange lack both D1 and D2.

S7: Eyes absent (Eya) is a family of tyrosine phophotases that are involved in eye development in many metazoan species [reviewed in 22, 23], including expression in all three *Drosophila* eye types (compound eye, ocelli, Bolwig's organ) [27]. We recovered a single *D. pulex* Eya gene in our analyses, and metazoan Eya proteins formed a monophyletic group. We found Protostome Eya proteins to be paraphyletic with the Deuterostome Eya genes monophyletic within this larger clade, consistent with the Neighbor Joining tree of Mazet et al [28]. Our reconciled tree analysis places the origin of Eya in the Eumetazoa lineage and implies three duplicates present early in eumetazoan history. Within the Pancrustacea, Eya is lost several times and never duplicated such that all pancrustaceans have a single copy of Eya with the *Daphnia* copy paralagous to the insect copy. Although several nodes are well-supported by aLRT analysis, and our analysis is similar to a previous result, the numerous losses implied by reconciled tree analysis illustrate substantial discordance between gene tree and species tree, especially due to the finding of paraphyletic protostome Eya genes, which could be subjected to more detailed scrutiny.

S8: Eyegone/Twin of Eyegone (Eyg/Toe) class genes have previously only been described in insects. They are similar to members of the Pax6 gene family, but have a reduced paired domain containing a motif similar to that seen in the 5a isoform of vertebrate Pax6 [29, 30], though these two genes are apparently not orthologous. We recovered a single *D. pulex* homologue of Eyg/Toe. Our analysis places Eyg/Toe as the sister group to PaxC genes. Our larger genomic analysis agrees with this, but includes a *C. elegans* gene in the Eyg/Toe clade. Upon closer inspection, this gene has a divergent "paired" domain that does not correspond with other genes in the Eyg/Toe clade. Because of this and its rather long-branch, we excluded it from the reconciled tree analysis, this indicates all presently known and clearly defined Eyg/Toe genes are from Pancrustacea. Including the worm gene does not significantly change our analysis, except that the origin of Eyg/Toe is pushed back to the Ecdysozoa node. Our reconciled tree shows a single duplication of Eyg/Toe within the insects, after the common ancestor of fly/bee and before the ancestor of fly/beetle. *Drosophila melanogaster* retains both copies, but *Tribolium* does not.

S9: Pax6 is an paired/homeodomain gene used in eye development throughout Metazoa [31]. Pax6 is a difficult gene to obtain consistent estimates of phylogentic history for, because the paired and homeodomains are conserved, but other regions evolve quickly. Therefore, while we used full-length protein sequence as bait, recovering two Pax6 genes from *D. pulex,* we built metazoan-wide gene phylogenies using only the paired and homeodomains (S10 B-1). We also added sequences from other arthropods (S10 B-2 and

B-3, Pax-6 clade from B-2 tree drawn with proportional branch lengths, see Methods) to better clarify when the Pax6 gene, in important gene in eye development [32], duplicated. Similar to previous analyses [33, 34], we recover a low-support monophyletic Pax-6 clade emerging at the base of Bilateria. Our additional arthropod sequences fell into two distinct clades named *toy* and *ey*, which split at or before the emergence of the pancrustacea. To further resolve this, we added in Genbank sequences from two other arthropods, the myriopod *Glomeris marginata* [35] and the chelicerate *Limulus polyphemus* [36]. Our phylogenetic analysis indicates that multiple arthropods including *G. marginata* have both *toy* and *ey* class genes, suggesting that Pax6 split early in arthropod evolution.

S10: Six 1/2 proteins, including Sine Oculis, are homeobox genes that fall within the larger Six-class family of genes and contain a ETSY tetrapeptide sequence [34]. They are part of the retinal determination network and are utilized in all three *Drosophila* eye types as part of photoreceptor determination [27] and are necessary for eye development in lophotrochozoan planarians [37]. We recovered a single Six 1/2 from *Daphnia.* Our genomic analysis suggests that Six 1/2 near the origin of metazoans: they are found in *N. victensis* but not in *Trichoplax* or *Monosiga*. Most of the Six 1/2 genes we recovered have the conserved tetrapeptide, although one *Nematostella* gene has the sequence ETTY and one silkworm gene has the tetrapeptide characteristic of Six 4/5 genes, ETVY. As this study is focused on gene history evolution, not gene function, we regard these two genes as Six 1/2-family members due to their placement on the phylogenetic tree. Our reconciled tree analysis suggests that Six 1/2 duplicated twice soon after its origination before the split between Cnidaria and Bilateria. All pancrustaceans have a single, monophyletic, Six 1/2.

Photoreceptor differentiation genes

S11: The Epidermal Growth Factor Receptor (EGFR) signaling pathway plays a role in many aspects of eye morphogenesis including determination of the photoreceptor field via Eya and Otd [27, 38, 39]. EGFR genes belong to a large class of Type 1 receptor tyrosine kinases with a PKc-EGFR domain preceeded by two recepL domains and a furin-like domain, and includes the ErbB family radiating in vertebrates [40]. Searches of the *Daphnia* genome recover a single EGFR homolog. Our genomic analysis found that most protostomes have only a single copy of EGFR. However, our reconciliation analysis suggests that a duplication of EGFR occurred in the lineage leading to Pancrustacea, but several losses occurred such that each pancrustacean has only one (or zero) EGFR-class genes.

S12: Glass is a C2H2 zinc finger domain protein with several conserved residues in the C2H2 linker regions. All known glass genes have 5 C2H2 domains except che-1, the *C. elegans* glass which has only 4 repeats [41]. Glass function has not been characterized outside of eye development, and has mostly been studied in insects where it acts in photoreceptor differentiation [41]. Our analysis finds the first Glass in a crustacean, *Daphnia*. Our analysis indicates a single glass gene orgin for all protostomes, with one loss in bee. Unlike previous studies, our genomic searches find two glass genes in *Nematostella*, one of which resembles the *C. elegans* glass in that it has only 4 repeats.

Our search also uncovered one previously undescribed lancelet glass gene. All of the Glass genes recovered by our analysis have conserved residues in the linker regions, suggesting that our search strategy can recover gene families with specific conserved sites, in addition to genes that share overall similarity. Our reconciled tree analysis suggests that Glass was duplicated in the protostome lineage, with different paralogues being lost in various species. If so, pancrustacean genomes possess different paralogs of Glass. For example, *T. castaneum* and *Drosophila* have different Glass paralogues, even though both species express Glass almost exclusively in the developing eyes [42-44]. Based on an SH test [4], our phylogenetic result is not significantly different than a previous study [41], when considering the relationships of genes used in both studies. Our new finding of different paralogs in fly and beetle results from analysis of different genes compared to a previous study [41].

S13: Kruppel (Kr) is a family of zinc-finger proteins found only in invertebrates. Our analysis recovered a single *D. pulex* gene within a monophyletic Kruppel group that excludes other zinc-finger domain proteins. Our alignments did not show any evidence of an “A-box” or “B-box” conserved in insect Kruppels, excepting a single conserved insect lysine in the A-box [42]. However, the zinc-fingers and linker regions were very well conserved in all pancrustacean Kr homologues. Our reconciled tree analysis indicates a single origin of Kr in the lineage leading to pancrustaceans with no losses.

S14: A gene implicated in the development of the Bolwig organ, Munster (Mu) is so far only characterized in *Drosophila* [45]. This gene cointains a homeobox domain with high similiarity to domains in Aristaless and Goosecoid, yet regions outside this domain offer no further clues as to possible gene homology. Searches against the *D. pulex* genome using full-length Mu from *Drosophila* only retrieved genes resembling Artistaless and Goosecoid. However, searches perfomed using only the conserved homeodomain revealed a Mu-type domain within *D. pulex* (S14A). We remain cautious in interpreting this result as evidence for Mu in *D. pulex*. As our genomic analysis did not recover genes outside of *D. pulex* and *Drosophila* that had any significant similarity to Mu, using either full length or homeobox sequence, we did not perform reconciliation analysis nor construct a metazoan-wide tree on this gene.

S15: Notch is a multi-domain protein with conserved NOD, EGF-repeat, and ankyrin domains and is found in all eumetazoans, with some domains found in *T. adhaerens* and *Monosiga* though these animals lack a true Notch [46, 47]. While Notch is involved in multitudinous developmental processes, it has a conserved role in neurogenesis and eye development in disparate species [48]. We find a single *D. pulex* Notch representative. The *Daphnia* gene groups with the other protostome Notch genes in our genomic analysis, which indicates that protostome Notch genes form a monophyletic clade. *H. robusta* (leech) Notch, groups apart from the rest of the Notch clade. This gene does contain Notch-specific domains so it is included in the Notch family. Reconciled tree analysis agrees with previous studies (ibid) in finding that Notch originated in the eumetazoa. We do not find any duplications of Notch in the pancrustaceans; each pancrustacean species studied has a single Notch homologue.

S16: Spitz is an Egf-repeat gene in the Erbb-ligand family that has only been characterized in invertebrates [40]. We recover a single Spitz from *D. pulex* in our analysis. This and our genomic tree support the previous findings that Spitz is an ecdysozoan specific gene. Our reconciled tree analysis suggests that Spitz underwent two duplications in the lineage leading to *Drosophila*, which has three Spitz genes, the other two being Gurken and Keren. Other analyses have found representatives of Gurken and Keren in other dipteran species, but not in other non-dipteran insects [40]. Our analysis supports this, as we find no other pancrustacean to have more than one Spitz.

S17: Visual System Homeobox (Vsx, called Ceh-10 in *C.elegans* and Chx in mouse) is a homeobox gene involved in the proliferation of retinal progenitor cells and the determination of neuronal projecting cells in vertebrates with similar roles hypothesized for *Drosophila* [49]. Our analysis found a single Vsx homologue in *D. pulex*. Genomic analysis found that beetle, bee and worm each also contain one Vsx homolog. Fly and silkworm each contain two vsx genes. Our phylogenetic analysis, coupled with tree reconciliation, indicate that each fly vsx gene has an ortholog in silkworm. Therefore, an insect-specific vsx gene duplication most likely predated fly+silk worm, but postdated the divergence of beetles and flies. In *Drosophila,* vsx genes are found in tandem on the X chromosome, suggesting an origin by tandem duplication, which also appears to be true for silkworm vsx1 and vsx2. In Lophotrochozoa, we found two vsx genes in *Capitella* and leech, along with three genes from *L. brevicolis*. Our phylogenetic analysis found strong support for separate clades of vertebrate Vsx1 and Vsx2 genes, as was found in a previous study [50]. However, our reconciliation analysis shows that there was not a strong correspondence between our vsx gene tree and established species relationships. Therefore, our vsx gene tree and our inference of gene duplication and loss, especially at deeper nodes, could benefit from additional more detailed phylogenetic analyses, perhaps including additional species when they become available.

Phototransduction genes

S18: Arrestin proteins act to quench the phototransduction cascade by de-activating opsins that have interacted with light [51]. The role of arrestin proteins in attenuating GPCR signaling is widespread, however arrestins thought to play a role specifically in phototransduction include the pancrustacean protein phosrestin [52] and the vertebrate visual arrestins [53]. Some evidence also suggests that the β arrestin from the tunicate ciona may also play a role in phototransduction [54]. Our arrestin phylogeny is similar to a previous study [55] and suggests a less dynamic history of duplication and loss compared to the other the phototransductive loci examined here. A single duplication of arrestin occurs prior to the bilateria while single duplication and loss events occur in the lineage leading to the beetle *Tribolium*.

S19: G_q-α is a subunit of the G protein: An early step in the rhabdomeric phototransduction cascade involves activation of $G_q\alpha$ subunits by opsin [51]. $G_q\alpha$ together with the full diversity of $G\alpha$ subunit classes was present prior to the origin of sponges, the earliest branching animal lineage [56]. Our analysis, which suggests a topology similar to previously reported studies [56], recovered two $G_q\alpha$ loci from *D. pulex* (Figures 3 and S19 A). These genes result from a duplication that took place prior

to the ecdysozoan lineage that in our analysis is represented by genome data from the nematode *C. elegans* and pancrustaceans (Fig 3). Our analysis also reveals duplications of $G_q\alpha$ loci prior to the origin of vertebrates and again in the lineage leading to teleosts. An additional duplication of $G_q\alpha$ occured in the annelid lineage leading to the leech *Helobdella robusta*. By our analyses, losses of $G_q\alpha$ occured only in the lineages leading to the silkworm *Bombyx mori* and the beetle *Tribolium castaneum*.

S20: Opsin, a member of the GPCR-class family of proteins, mediates phototransduction cascades in animals [51]. We included only rhabdomeric opsins in our counts of opsin gene duplication and loss. Detailed phylogenetic analyses for this gene family are presented elsewhere (Colbourne J et al: Genome Biology of the Model Crustacean *Daphnia pulex*, submitted). Consistent with previous analyses [57], our genomic analysis recovers rhabdomeric opsins from the bilaterian animals only. Our RTA analysis identified 43 well-supported gene duplication events in the evolutionary history of rhabdomeric opsins across all taxa examined, far more than any other phototransduction locus considered here. Twenty-five of these duplications occurred within the *D. pulex* lineage alone. This analysis also recorded 13 loss events for rhabdomeric opsins.

S21: PLC is an intermediary enzyme in the rhabdomeric phototransduction cascade [51]. Our analysis suggests that PLC loci were present at least as early as the split of the parazoan *T. adhaerens* with other animals, thus predating the origin of the rhabdomeric- and arthops-mediated phtotrasduction cascades (Fig 3). Our phylogeny of PLC contains two clades (S2 B) both derived from an ancient gene duplication event that occurred prior to the origin of the lineage leading to *T. adhaerens*. We do not recover duplication or loss events for PLC loci in *D. pulex* whose genome preserves both of the ancestral copies (S21 A). We recover a single PLC duplication that occurred prior to bilaterian animals, and in the separate lineages leading to *T. rubripes*, *G.gallus*, *H. robusta*, *C. elegans*, and *A. mellifera*. Two PLC duplications took place in the lineage leading to the vertebrates and in that leading to humans while three PLC duplications took place in the lineage leading to the lancelet *B.floridae*. We also recover single PLC loss events in the lineages leading to tetrapods, lophotrochozoan animals which include annelids and mollusks and in the separate lineages leading to *C. intestinallis*, *M.musculus*, humans, *G. gallus*, and *A. mellifera*. Two PLC loss events were recovered in the separate lineages leading to *D. rerio* and *C. elegans*.

S22: Transient Receptor Potential ion channel C (TRPC) proteins are a large class of calcium ion channels present in both deuterostomes and protostomes [58]. Phototransductive TRPCs are however found only in rhabdomeric photoreceptors, such as those that mediate vision in pancrustacea [51] and the intrinsically photosensitive retinal ganglion cells (ipRGCs) of the vertebrate retina [59]. Here, TRPCs provide the functional cell-physiological response to rhabdomeric phototransduction, which ultimately leads to the opening of TRPCs and the depolarization of rhabdomeric photoreceptor cells. Two closely related TRP ion channels TRP-like and TRP Gamma are also highly expressed in rhabdomeric photoreceptors and can facilitate phototransduction-mediated hyperpolarization [60, 61]. Like other pancrustaceans, the genome of *D. pulex* contains one locus for each of these phototransduction TRP loci.

Our phylogeny suggests that the phototransductive TRPCs are the sister to a clade of TRP4 and TRP5 ion channel classes known from deuterostomes. Our phylogeny for TRPCs suggests a complicated history of duplication and loss during animal evolution. Three duplications of TRPC loci predate the bilaterians, chordates and the lineage leading to the lancelet respectively. Two TRPC duplications occur prior to separate pancustacean and deuterostome lineages. Finally, single duplications of TRPC loci occur in the lineages leading to the mollusks, tetrapods and teleosts and along the branches leading to the silkworm, leech and limpet. According to our reconciled tree analysis, losses of TRP loci were also common in animal evolution with most occurring within the deuterostomes.

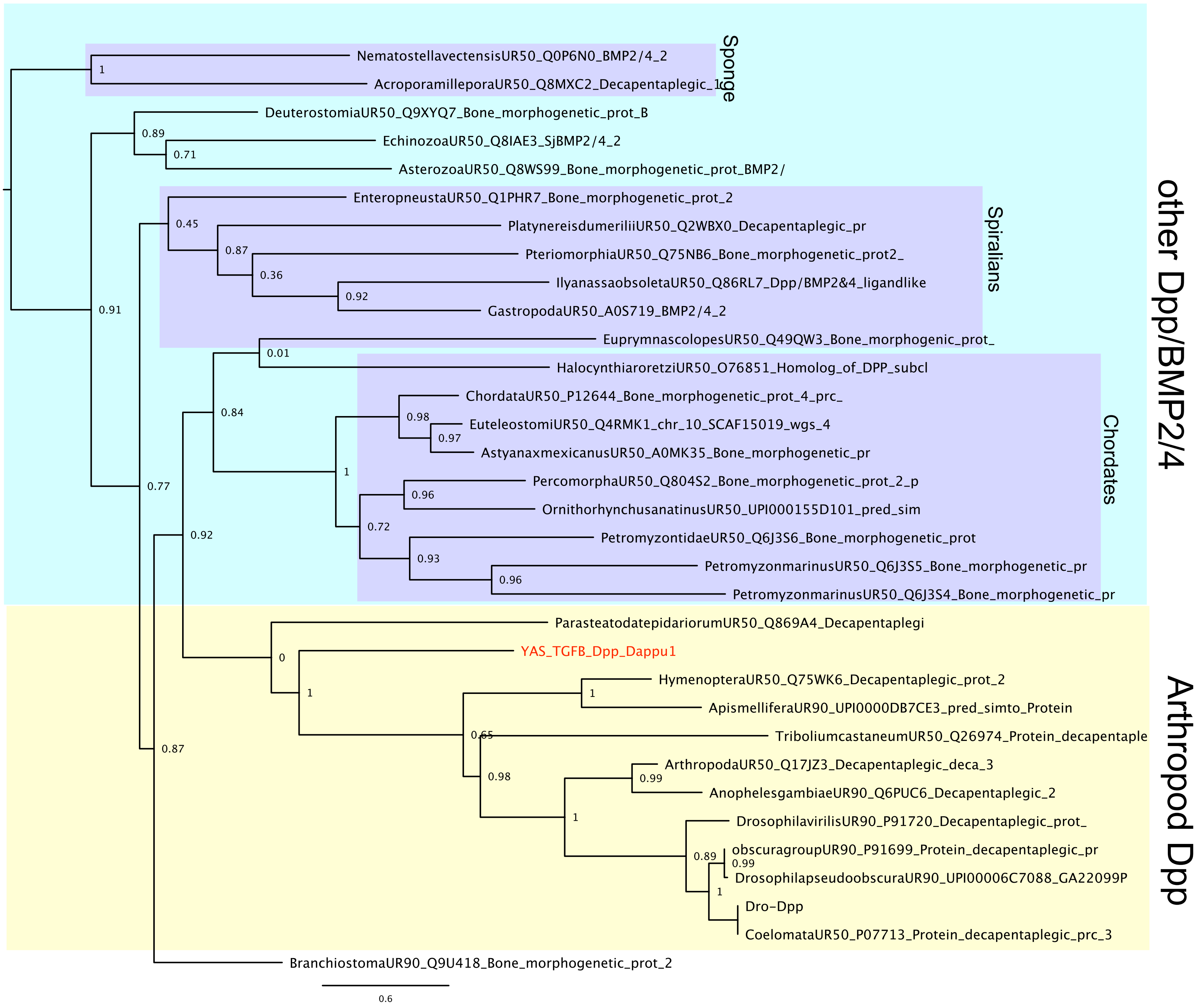
NematostellavectensisUR50_Q0P6N0_BMP2/4_2
AcroporamilleporaUR50_Q8MXC2_Decapentaplegic_1
Sponge
DeuterostomiaUR50_Q9XYQ7_Bone_morphogenetic_prot_B
EchinozoaUR50_Q8IAE3_SjBMP2/4_2
AsterozoaUR50_Q8WS99_Bone_morphogenetic_prot_BMP2/
EnteropneustaUR50_Q1PHR7_Bone_morphogenetic_prot_2
PlatynereisdumeriliiUR50_Q2WBX0_Decapentaplegic_pr
PteriomorphiaUR50_Q75NB6_Bone_morphogenetic_prot2_
IlyanassaobsoletaUR50_Q86RL7_Dpp/BMP2&4_ligandlike
GastropodaUR50_A0S719_BMP2/4_2
Spiralians
EuprymnascolopesUR50_Q49QW3_Bone_morphogenic_prot_
HalocynthiaroretziUR50_O76851_Homolog_of_DPP_subcl
ChordataUR50_P12644_Bone_morphogenetic_prot_4_prc_
EuteleostomiUR50_Q4RMK1_chr_10_SCAF15019_wgs_4
AstyanaxmexicanusUR50_A0MK35_Bone_morphogenetic_pr
PercomorphaUR50_Q804S2_Bone_morphogenetic_prot_2_p
OrnithorhynchusanatinusUR50_UPI000155D101_pred_sim
PetromyzontidaeUR50_Q6J3S6_Bone_morphogenetic_prot
PetromyzonmarinusUR50_Q6J3S5_Bone_morphogenetic_pr
PetromyzonmarinusUR50_Q6J3S4_Bone_morphogenetic_pr
Chordates
other Dpp/BMP2/4
ParasteatodatepidariorumUR50_Q869A4_Decapentaplegi
YAS_TGFB_Dpp_Dappu1
HymenopteraUR50_Q75WK6_Decapentaplegic_prot_2
ApismelliferaUR90_UPI0000DB7CE3_pred_simto_Protein
TriboliumcastaneumUR50_Q26974_Protein_decapentaple
ArthropodaUR50_Q17JZ3_Decapentaplegic_deca_3
AnophelesgambiaeUR90_Q6PUC6_Decapentaplegic_2
DrosophilavirilisUR90_P91720_Decapentaplegic_prot_
obscuragroupUR90_P91699_Protein_decapentaplegic_pr
DrosophilapseudoobscuraUR90_UPI00006C7088_GA22099P
Dro-Dpp
CoelomataUR50_P07713_Protein_decapentaplegic_prc_3
Arthropod Dpp
BranchiostomaUR90_Q9U418_Bone_morphogenetic_prot_2
1
0.89
0.71
0.91
0.45
0.87
0.36
0.92
0.01
0.84
0.98
0.97
1
0.96
0.72
0.93
0.96
0.77
0.92
0
1
1
0.65
0.98
0.99
1
0.89
0.99
1
0.87
0.6

S1 A

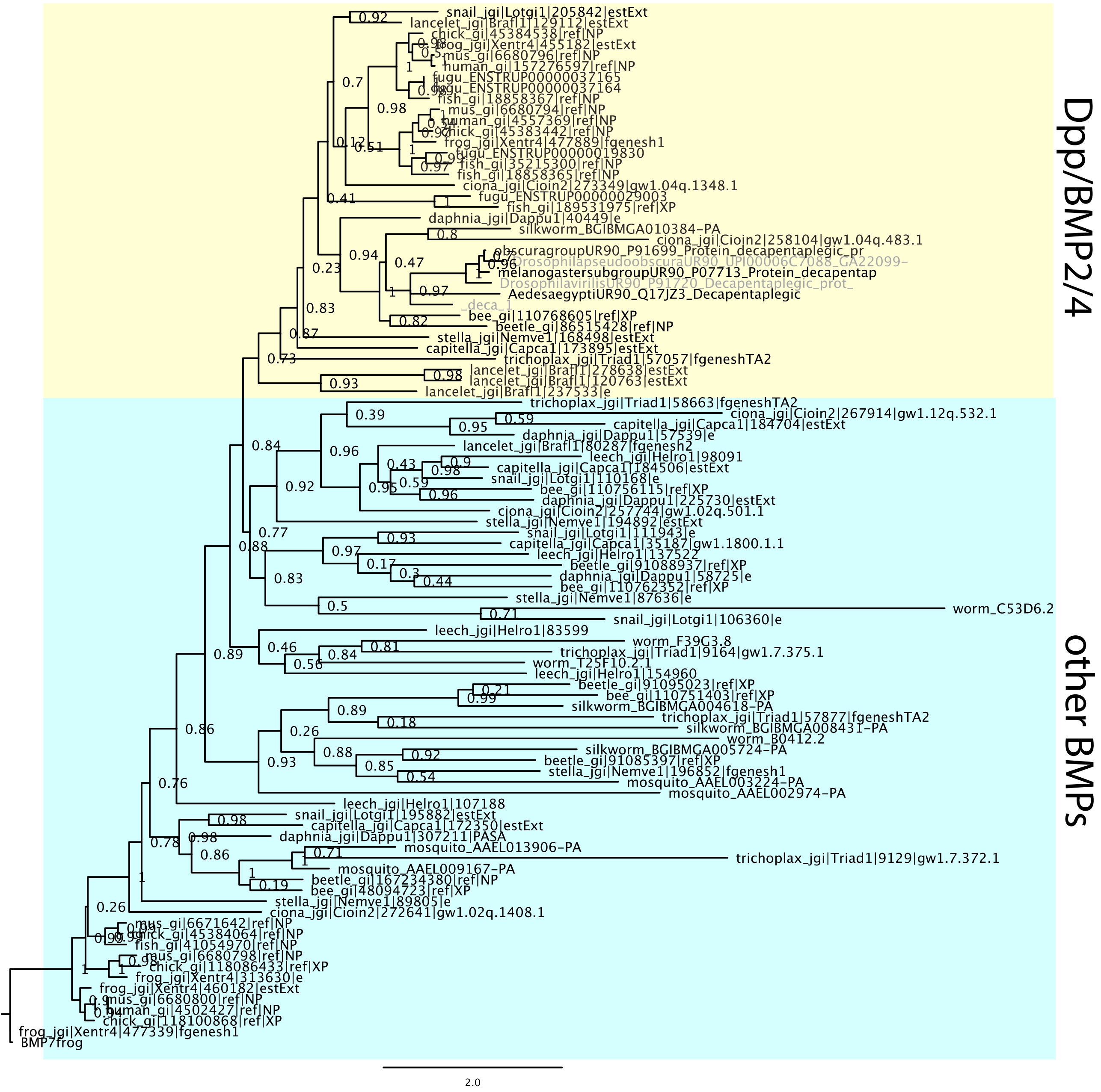
Dpp/BMP2/4
other BMPs
snail_jgi|Lotgi1|205842|estExt
lancelet_jgi|Brafl1|129112|estExt
chick_gi|45384538|ref|NP
frog_jgi|Xentr4|455182|estExt
mus_gi|6680796|ref|NP
human_gi|157276597|ref|NP
fugu_ENSTRUP00000037165
fugu_ENSTRUP00000037164
fish_gi|18858367|ref|NP
mus_gi|6680794|ref|NP
human_gi|4557369|ref|NP
chick_gi|45383442|ref|NP
frog_jgi|Xentr4|477889|fgenesh1
fugu_ENSTRUP00000019830
fish_gi|35215300|ref|NP
fish_gi|18858365|ref|NP
ciona_jgi|Cioin2|273349|gw1.04q.1348.1
fugu_ENSTRUP00000029003
fish_gi|189531975|ref|XP
daphnia_jgi|Dappu1|40449|e
silkworm_BGIBMGA010384-PA
ciona_jgi|Cioin2|258104|gw1.04q.483.1
obscuragroupUR90_P91699_Protein_decapentaplegic_pr
DrosophilapseudoobscuraUR90_UPI00006C7088_GA22099-
melanogastersubgroupUR90_P07713_Protein_decapentap
DrosophilavirilisUR90_P91720_Decapentaplegic_prot_
AedesaegyptiUR90_Q17JZ3_Decapentaplegic
_deca_1
bee_gi|110768605|ref|XP
beetle_gi|86515428|ref|NP
stella_jgi|Nemve1|168498|estExt
capitella_jgi|Capca1|173895|estExt
trichoplax_jgi|Triad1|57057|fgeneshTA2
lancelet_jgi|Brafl1|278638|estExt
lancelet_jgi|Brafl1|120763|estExt
lancelet_jgi|Brafl1|237533|e
trichoplax_jgi|Triad1|58663|fgeneshTA2
ciona_jgi|Cioin2|267914|gw1.12q.532.1
capitella_jgi|Capca1|184704|estExt
daphnia_jgi|Dappu1|57539|e
lancelet_jgi|Brafl1|80287|fgenesh2
leech_jgi|Helro1|98091
capitella_jgi|Capca1|184506|estExt
snail_jgi|Lotgi1|110168|e
bee_gi|110756115|ref|XP
daphnia_jgi|Dappu1|225730|estExt
ciona_jgi|Cioin2|257744|gw1.02q.501.1
stella_jgi|Nemve1|194892|estExt
snail_jgi|Lotgi1|111943|e
capitella_jgi|Capca1|35187|gw1.1800.1.1
leech_jgi|Helro1|137522
beetle_gi|91088937|ref|XP
daphnia_jgi|Dappu1|58725|e
bee_gi|110762352|ref|XP
stella_jgi|Nemve1|87636|e
worm_C53D6.2
snail_jgi|Lotgi1|106360|e
leech_jgi|Helro1|83599
worm_F39G3.8
trichoplax_jgi|Triad1|9164|gw1.7.375.1
worm_T25F10.2.1
leech_jgi|Helro1|154960
beetle_gi|91095023|ref|XP
bee_gi|110751403|ref|XP
silkworm_BGIBMGA004618-PA
trichoplax_jgi|Triad1|57877|fgeneshTA2
silkworm_BGIBMGA008431-PA
worm_B0412.2
silkworm_BGIBMGA005724-PA
beetle_gi|91085397|ref|XP
stella_jgi|Nemve1|196852|fgenesh1
mosquito_AAEL003224-PA
mosquito_AAEL002974-PA
leech_jgi|Helro1|107188
snail_jgi|Lotgi1|195882|estExt
capitella_jgi|Capca1|172350|estExt
daphnia_jgi|Dappu1|307211|PASA
mosquito_AAEL013906-PA
trichoplax_jgi|Triad1|9129|gw1.7.372.1
mosquito_AAEL009167-PA
beetle_gi|167234380|ref|NP
bee_gi|48094723|ref|XP
stella_jgi|Nemve1|89805|e
ciona_jgi|Cioin2|272641|gw1.02q.1408.1
mus_gi|6671642|ref|NP
chick_gi|45384064|ref|NP
fish_gi|41054970|ref|NP
mus_gi|6680798|ref|NP
chick_gi|118086433|ref|XP
frog_jgi|Xentr4|313630|e
frog_jgi|Xentr4|460182|estExt
mus_gi|6680800|ref|NP
human_gi|4502427|ref|NP
chick_gi|118100868|ref|XP
frog_jgi|Xentr4|477339|fgenesh1
BMP7frog
2.0

S1 B

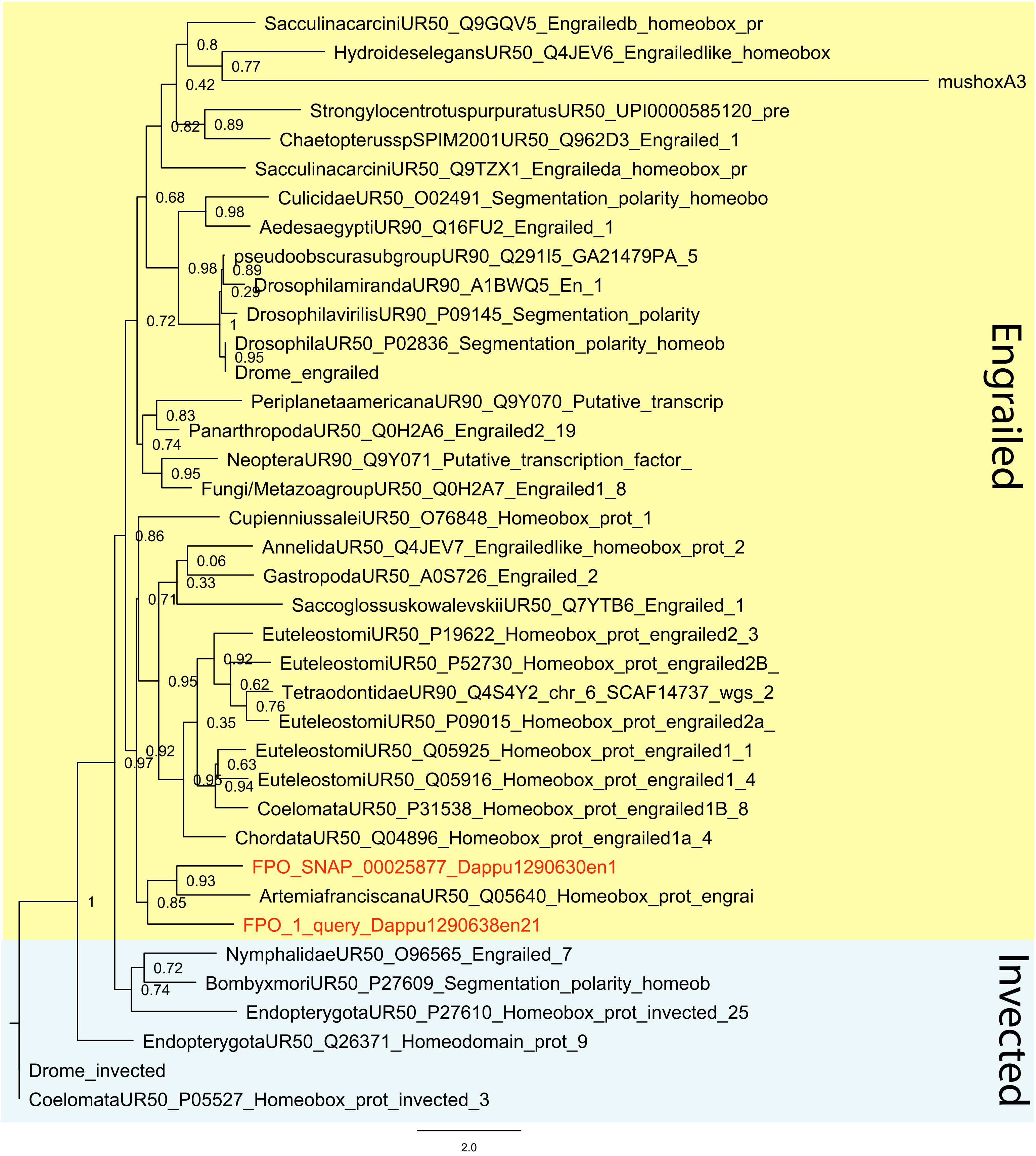
SacculinacarciniUR50_Q9GQV5_Engrailedb_homeobox_pr
HydroideselegansUR50_Q4JEV6_Engrailedlike_homeobox
mushoxA3
StrongylocentrotuspurpuratusUR50_UPI0000585120_pre
ChaetopterusspSPIM2001UR50_Q962D3_Engrailed_1
SacculinacarciniUR50_Q9TZX1_Engraileda_homeobox_pr
CulicidaeUR50_O02491_Segmentation_polarity_homeobo
AedesaegyptiUR90_Q16FU2_Engrailed_1
pseudoobscurasubgroupUR90_Q291I5_GA21479PA_5
DrosophilamirandaUR90_A1BWQ5_En_1
DrosophilavirilisUR90_P09145_Segmentation_polarity
DrosophilaUR50_P02836_Segmentation_polarity_homeob
Drome_engrailed
PeriplanetaamericanaUR90_Q9Y070_Putative_transcrip
PanarthropodaUR50_Q0H2A6_Engrailed2_19
NeopteraUR90_Q9Y071_Putative_transcription_factor_
Fungi/MetazoagroupUR50_Q0H2A7_Engrailed1_8
CupienniussaleiUR50_O76848_Homeobox_prot_1
AnnelidaUR50_Q4JEV7_Engrailedlike_homeobox_prot_2
GastropodaUR50_A0S726_Engrailed_2
SaccoglossuskowalevskiiUR50_Q7YTB6_Engrailed_1
EuteleostomiUR50_P19622_Homeobox_prot_engrailed2_3
EuteleostomiUR50_P52730_Homeobox_prot_engrailed2B_
TetraodontidaeUR90_Q4S4Y2_chr_6_SCAF14737_wgs_2
EuteleostomiUR50_P09015_Homeobox_prot_engrailed2a_
EuteleostomiUR50_Q05925_Homeobox_prot_engrailed1_1
EuteleostomiUR50_Q05916_Homeobox_prot_engrailed1_4
CoelomataUR50_P31538_Homeobox_prot_engrailed1B_8
ChordataUR50_Q04896_Homeobox_prot_engrailed1a_4
FPO_SNAP_00025877_Dappu1290630en1
ArtemiafranciscanaUR50_Q05640_Homeobox_prot_engrai
FPO_1_query_Dappu1290638en21
NymphalidaeUR50_O96565_Engrailed_7
BombyxmoriUR50_P27609_Segmentation_polarity_homeob
EndopterygotaUR50_P27610_Homeobox_prot_invected_25
EndopterygotaUR50_Q26371_Homeodomain_prot_9
Drome_invected
CoelomataUR50_P05527_Homeobox_prot_invected_3
Engrailed
Invected
2.0

S2 A

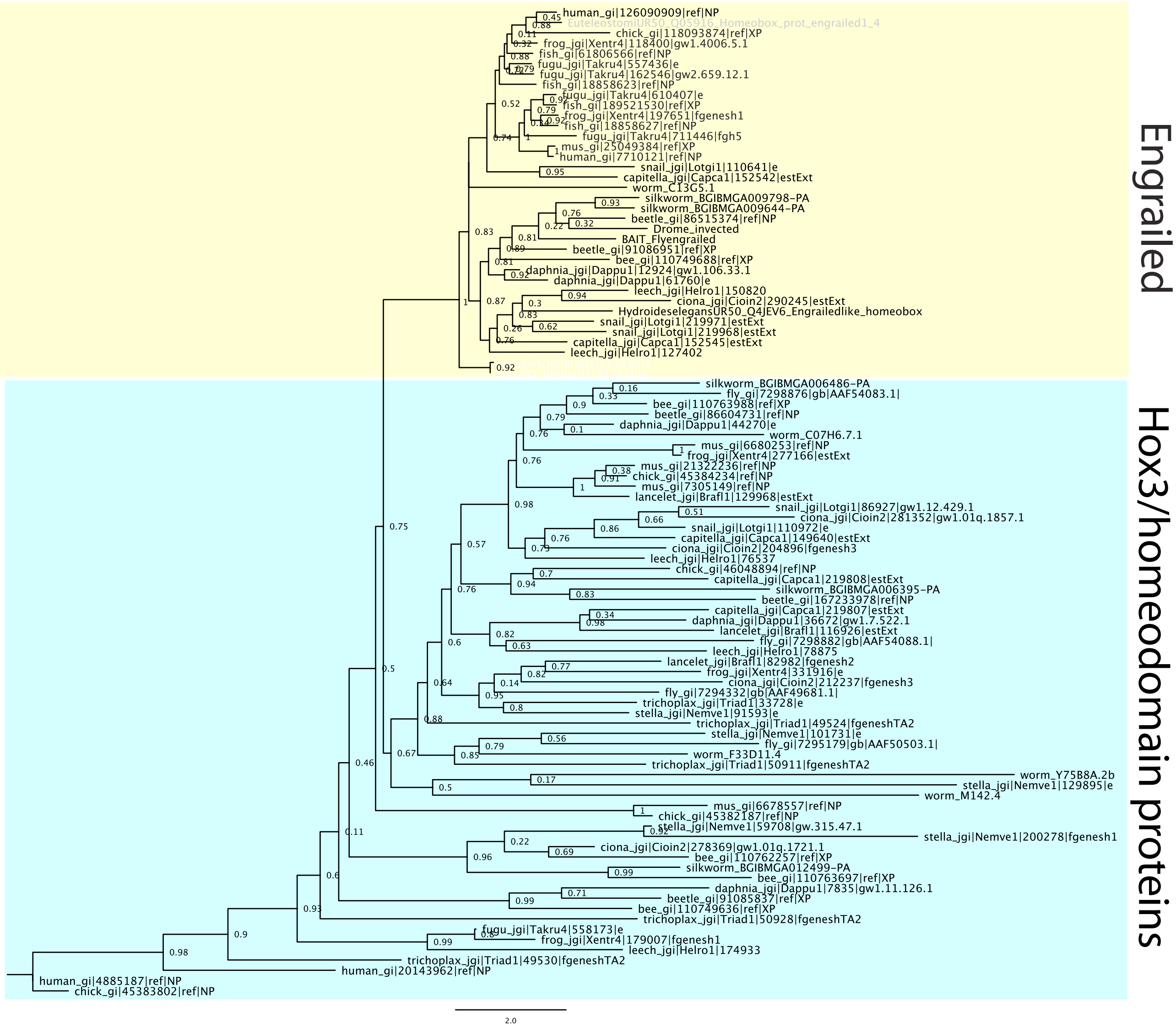
Engrailed
Hox3/homeodomain proteins
human_gi|126090909|ref|NP
EuteleostomiUR50_Q05916_Homeobox_prot_engrailed1_4
chick_gi|118093874|ref|XP
frog_jgi|Xentr4|118400|gw1.4006.5.1
fish_gi|61806566|ref|NP
fugu_jgi|Takru4|557436|e
fugu_jgi|Takru4|162546|gw2.659.12.1
fish_gi|18858623|ref|NP
fugu_jgi|Takru4|610407|e
fish_gi|189521530|ref|XP
frog_jgi|Xentr4|197651|fgenesh1
fish_gi|18858627|ref|NP
fugu_jgi|Takru4|711446|fgh5
mus_gi|25049384|ref|XP
human_gi|7710121|ref|NP
snail_jgi|Lotgi1|110641|e
capitella_jgi|Capca1|152542|estExt
worm_C13G5.1
silkworm_BGIBMGA009798-PA
silkworm_BGIBMGA009644-PA
beetle_gi|86515374|ref|NP
Drome_invected
BAIT_Flyengrailed
beetle_gi|91086951|ref|XP
bee_gi|110749688|ref|XP
daphnia_jgi|Dappu1|12924|gw1.106.33.1
daphnia_jgi|Dappu1|61760|e
leech_jgi|Helro1|150820
ciona_jgi|Cioin2|290245|estExt
HydroideselegansUR50_Q4JEV6_Engrailedlike_homeobox
snail_jgi|Lotgi1|219971|estExt
snail_jgi|Lotgi1|219968|estExt
capitella_jgi|Capca1|152545|estExt
leech_jgi|Helro1|127402
silkworm_BGIBMGA006486-PA
fly_gi|7298876|gb|AAF54083.1|
bee_gi|110763988|ref|XP
beetle_gi|86604731|ref|NP
daphnia_jgi|Dappu1|44270|e
worm_C07H6.7.1
mus_gi|6680253|ref|NP
frog_jgi|Xentr4|277166|estExt
mus_gi|21322236|ref|NP
chick_gi|45384234|ref|NP
mus_gi|7305149|ref|NP
lancelet_jgi|Brafl1|129968|estExt
snail_jgi|Lotgi1|86927|gw1.12.429.1
ciona_jgi|Cioin2|281352|gw1.01q.1857.1
snail_jgi|Lotgi1|110972|e
capitella_jgi|Capca1|149640|estExt
ciona_jgi|Cioin2|204896|fgenesh3
leech_jgi|Helro1|76537
chick_gi|46048894|ref|NP
capitella_jgi|Capca1|219808|estExt
silkworm_BGIBMGA006395-PA
beetle_gi|167233978|ref|NP
capitella_jgi|Capca1|219807|estExt
daphnia_jgi|Dappu1|36672|gw1.7.522.1
lancelet_jgi|Brafl1|116926|estExt
fly_gi|7298882|gb|AAF54088.1|
leech_jgi|Helro1|78875
lancelet_jgi|Brafl1|82982|fgenesh2
frog_jgi|Xentr4|331916|e
ciona_jgi|Cioin2|212237|fgenesh3
fly_gi|7294332|gb|AAF49681.1|
trichoplax_jgi|Triad1|33728|e
stella_jgi|Nemve1|91593|e
trichoplax_jgi|Triad1|49524|fgeneshTA2
stella_jgi|Nemve1|101731|e
fly_gi|7295179|gb|AAF50503.1|
worm_F33D11.4
trichoplax_jgi|Triad1|50911|fgeneshTA2
worm_Y75B8A.2b
stella_jgi|Nemve1|129895|e
worm_M142.4
mus_gi|6678557|ref|NP
chick_gi|45382187|ref|NP
stella_jgi|Nemve1|59708|gw.315.47.1
stella_jgi|Nemve1|200278|fgenesh1
ciona_jgi|Cioin2|278369|gw1.01q.1721.1
bee_gi|110762257|ref|XP
silkworm_BGIBMGA012499-PA
bee_gi|110763697|ref|XP
daphnia_jgi|Dappu1|7835|gw1.11.126.1
beetle_gi|91085837|ref|XP
bee_gi|110749636|ref|XP
trichoplax_jgi|Triad1|50928|fgeneshTA2
fugu_jgi|Takru4|558173|e
frog_jgi|Xentr4|179007|fgenesh1
leech_jgi|Helro1|174933
trichoplax_jgi|Triad1|49530|fgeneshTA2
human_gi|20143962|ref|NP
human_gi|4885187|ref|NP
chick_gi|45383802|ref|NP
2.0

S2 B

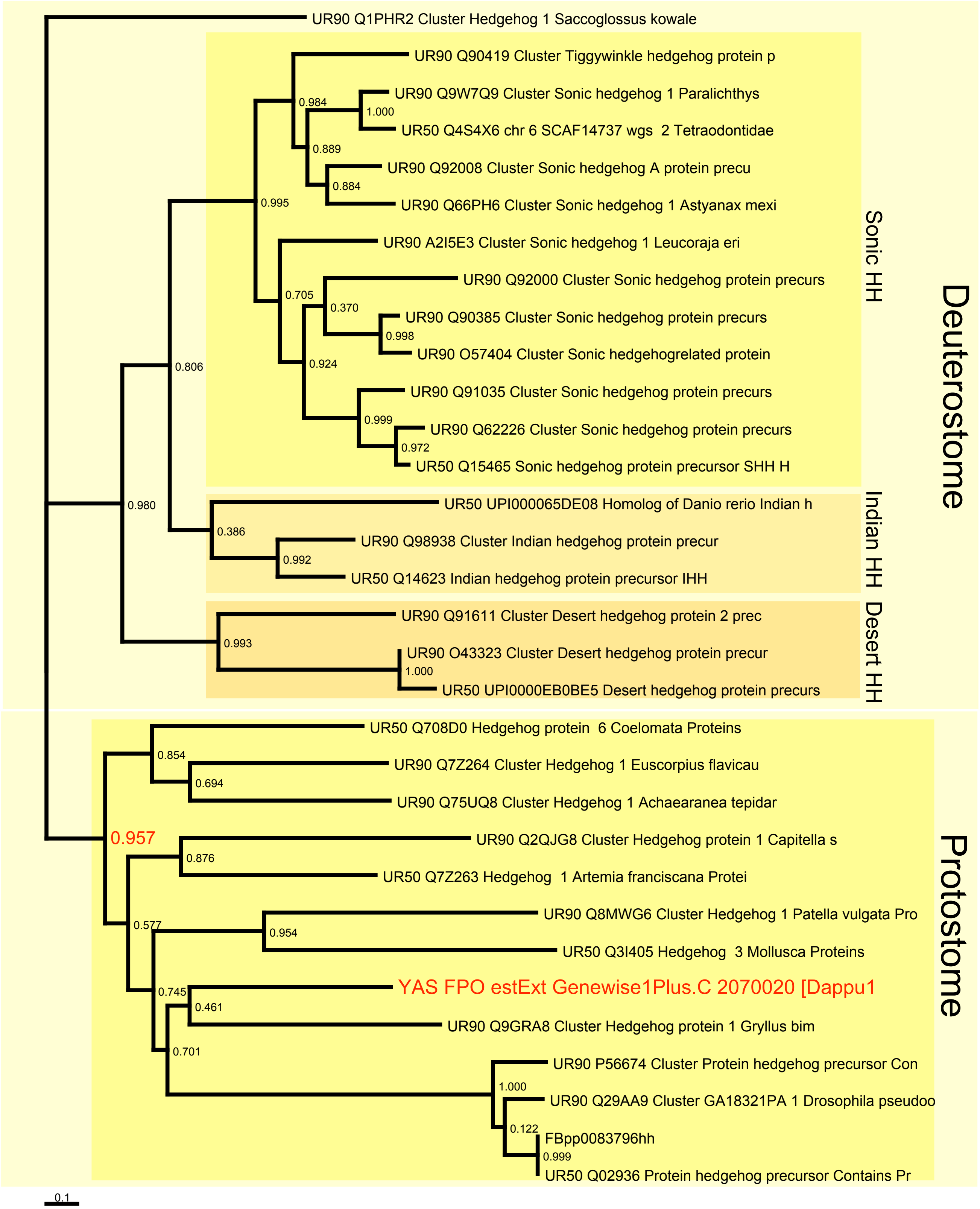

UR90 Q1PHR2 Cluster Hedgehog 1 Saccoglossus kowale
UR90 Q90419 Cluster Tiggywinkle hedgehog protein p
0.984
UR90 Q9W7Q9 Cluster Sonic hedgehog 1 Paralichthys
1.000
UR50 Q4S4X6 chr 6 SCAF14737 wgs 2 Tetraodontidae
0.889
UR90 Q92008 Cluster Sonic hedgehog A protein precu
0.884
UR90 Q66PH6 Cluster Sonic hedgehog 1 Astyanax mexi
0.995
UR90 A2I5E3 Cluster Sonic hedgehog 1 Leucoraja eri
UR90 Q92000 Cluster Sonic hedgehog protein precurs
0.705
0.370
UR90 Q90385 Cluster Sonic hedgehog protein precurs
0.998
UR90 O57404 Cluster Sonic hedgehogrelated protein
0.924
0.806
UR90 Q91035 Cluster Sonic hedgehog protein precurs
0.999
UR90 Q62226 Cluster Sonic hedgehog protein precurs
0.972
UR50 Q15465 Sonic hedgehog protein precursor SHH H
Sonic HH
0.980
UR50 UPI000065DE08 Homolog of Danio rerio Indian h
0.386
UR90 Q98938 Cluster Indian hedgehog protein precur
0.992
UR50 Q14623 Indian hedgehog protein precursor IHH
Indian HH
UR90 Q91611 Cluster Desert hedgehog protein 2 prec
0.993
UR90 O43323 Cluster Desert hedgehog protein precur
1.000
UR50 UPI0000EB0BE5 Desert hedgehog protein precurs
Desert HH
Deuterostome
Hedgehog
UR50 Q708D0 Hedgehog protein 6 Coelomata Proteins
0.854
UR90 Q7Z264 Cluster Hedgehog 1 Euscorpius flavicau
0.694
UR90 Q75UQ8 Cluster Hedgehog 1 Achaearanea tepidar
0.957
UR90 Q2QJG8 Cluster Hedgehog protein 1 Capitella s
0.876
UR50 Q7Z263 Hedgehog 1 Artemia franciscana Protei
UR90 Q8MWG6 Cluster Hedgehog 1 Patella vulgata Pro
0.577
0.954
UR50 Q3I405 Hedgehog 3 Mollusca Proteins
0.745
YAS FPO estExt Genewise1Plus.C 2070020 [Dappu1
0.461
UR90 Q9GRA8 Cluster Hedgehog protein 1 Gryllus bim
0.701
UR90 P56674 Cluster Protein hedgehog precursor Con
1.000
UR90 Q29AA9 Cluster GA18321PA 1 Drosophila pseudoo
0.122
FBpp0083796hh
0.999
UR50 Q02936 Protein hedgehog precursor Contains Pr
Protostome
0.1

S3 A

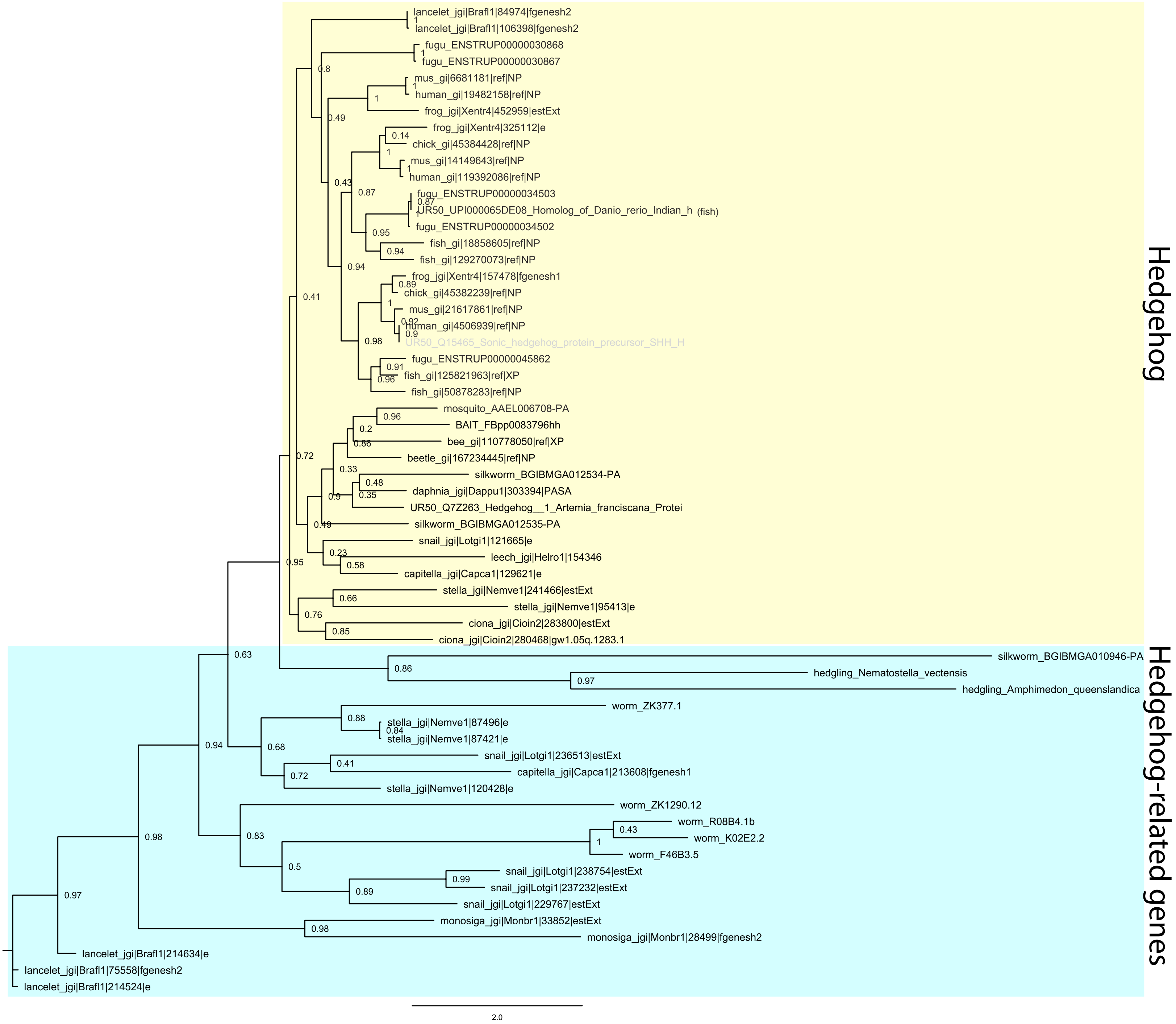
Hedgehog
Hedgehog-related genes
lancelet_jgi|Brafl1|84974|fgenesh2
lancelet_jgi|Brafl1|106398|fgenesh2
fugu_ENSTRUP00000030868
fugu_ENSTRUP00000030867
mus_gi|6681181|ref|NP
human_gi|19482158|ref|NP
frog_jgi|Xentr4|452959|estExt
frog_jgi|Xentr4|325112|e
chick_gi|45384428|ref|NP
mus_gi|14149643|ref|NP
human_gi|119392086|ref|NP
fugu_ENSTRUP00000034503
UR50_UPI000065DE08_Homolog_of_Danio_rerio_Indian_h (fish)
fugu_ENSTRUP00000034502
fish_gi|18858605|ref|NP
fish_gi|129270073|ref|NP
frog_jgi|Xentr4|157478|fgenesh1
chick_gi|45382239|ref|NP
mus_gi|21617861|ref|NP
human_gi|4506939|ref|NP
UR50_Q15465_Sonic_hedgehog_protein_precursor_SHH_H
fugu_ENSTRUP00000045862
fish_gi|125821963|ref|XP
fish_gi|50878283|ref|NP
mosquito_AAEL006708-PA
BAIT_FBpp0083796hh
bee_gi|110778050|ref|XP
beetle_gi|167234445|ref|NP
silkworm_BGIBMGA012534-PA
daphnia_jgi|Dappu1|303394|PASA
UR50_Q7Z263_Hedgehog__1_Artemia_franciscana_Protei
silkworm_BGIBMGA012535-PA
snail_jgi|Lotgi1|121665|e
leech_jgi|Helro1|154346
capitella_jgi|Capca1|129621|e
stella_jgi|Nemve1|241466|estExt
stella_jgi|Nemve1|95413|e
ciona_jgi|Cioin2|283800|estExt
ciona_jgi|Cioin2|280468|gw1.05q.1283.1
silkworm_BGIBMGA010946-PA
hedgling_Nematostella_vectensis
hedgling_Amphimedon_queenslandica
worm_ZK377.1
stella_jgi|Nemve1|87496|e
stella_jgi|Nemve1|87421|e
snail_jgi|Lotgi1|236513|estExt
capitella_jgi|Capca1|213608|fgenesh1
stella_jgi|Nemve1|120428|e
worm_ZK1290.12
worm_R08B4.1b
worm_K02E2.2
worm_F46B3.5
snail_jgi|Lotgi1|238754|estExt
snail_jgi|Lotgi1|237232|estExt
snail_jgi|Lotgi1|229767|estExt
monosiga_jgi|Monbr1|33852|estExt
monosiga_jgi|Monbr1|28499|fgenesh2
lancelet_jgi|Brafl1|214634|e
lancelet_jgi|Brafl1|75558|fgenesh2
lancelet_jgi|Brafl1|214524|e
2.0

S3 B

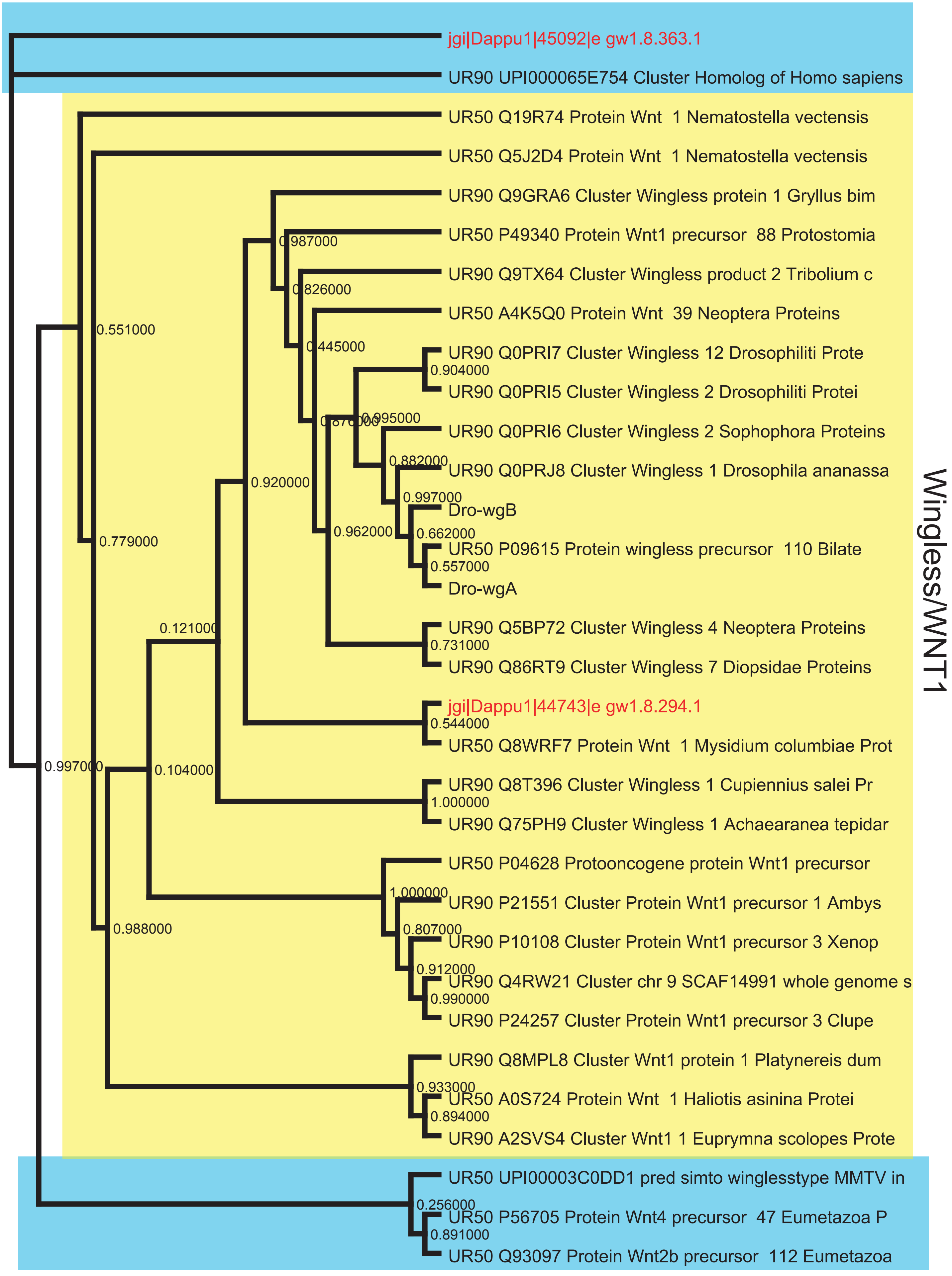
jgi|Dappu1|45092|e gw1.8.363.1
UR90 UPI000065E754 Cluster Homolog of Homo sapiens
UR50 Q19R74 Protein Wnt 1 Nematostella vectensis
UR50 Q5J2D4 Protein Wnt 1 Nematostella vectensis
UR90 Q9GRA6 Cluster Wingless protein 1 Gryllus bim
UR50 P49340 Protein Wnt1 precursor 88 Protostomia
UR90 Q9TX64 Cluster Wingless product 2 Tribolium c
UR50 A4K5Q0 Protein Wnt 39 Neoptera Proteins
UR90 Q0PRI7 Cluster Wingless 12 Drosophiliti Prote
UR90 Q0PRI5 Cluster Wingless 2 Drosophiliti Protei
UR90 Q0PRI6 Cluster Wingless 2 Sophophora Proteins
UR90 Q0PRJ8 Cluster Wingless 1 Drosophila ananassa
Dro-wgB
UR50 P09615 Protein wingless precursor 110 Bilate
Dro-wgA
UR90 Q5BP72 Cluster Wingless 4 Neoptera Proteins
UR90 Q86RT9 Cluster Wingless 7 Diopsidae Proteins
jgi|Dappu1|44743|e gw1.8.294.1
UR50 Q8WRF7 Protein Wnt 1 Mysidium columbiae Prot
UR90 Q8T396 Cluster Wingless 1 Cupiennius salei Pr
UR90 Q75PH9 Cluster Wingless 1 Achaearanea tepidar
UR50 P04628 Protooncogene protein Wnt1 precursor
UR90 P21551 Cluster Protein Wnt1 precursor 1 Ambys
UR90 P10108 Cluster Protein Wnt1 precursor 3 Xenop
UR90 Q4RW21 Cluster chr 9 SCAF14991 whole genome s
UR90 P24257 Cluster Protein Wnt1 precursor 3 Clupe
UR90 Q8MPL8 Cluster Wnt1 protein 1 Platynereis dum
UR50 A0S724 Protein Wnt 1 Haliotis asinina Protei
UR90 A2SVS4 Cluster Wnt1 1 Euprymna scolopes Prote
UR50 UPI00003C0DD1 pred simto winglesstype MMTV in
UR50 P56705 Protein Wnt4 precursor 47 Eumetazoa P
UR50 Q93097 Protein Wnt2b precursor 112 Eumetazoa
0.987000
0.826000
0.551000
0.445000
0.904000
0.995000
0.882000
0.920000
0.997000
0.962000
0.662000
0.557000
0.779000
0.121000
0.731000
0.544000
0.997000
0.104000
1.000000
1.000000
0.807000
0.988000
0.912000
0.990000
0.933000
0.894000
0.256000
0.891000
Wingless/WNT1

S4 A

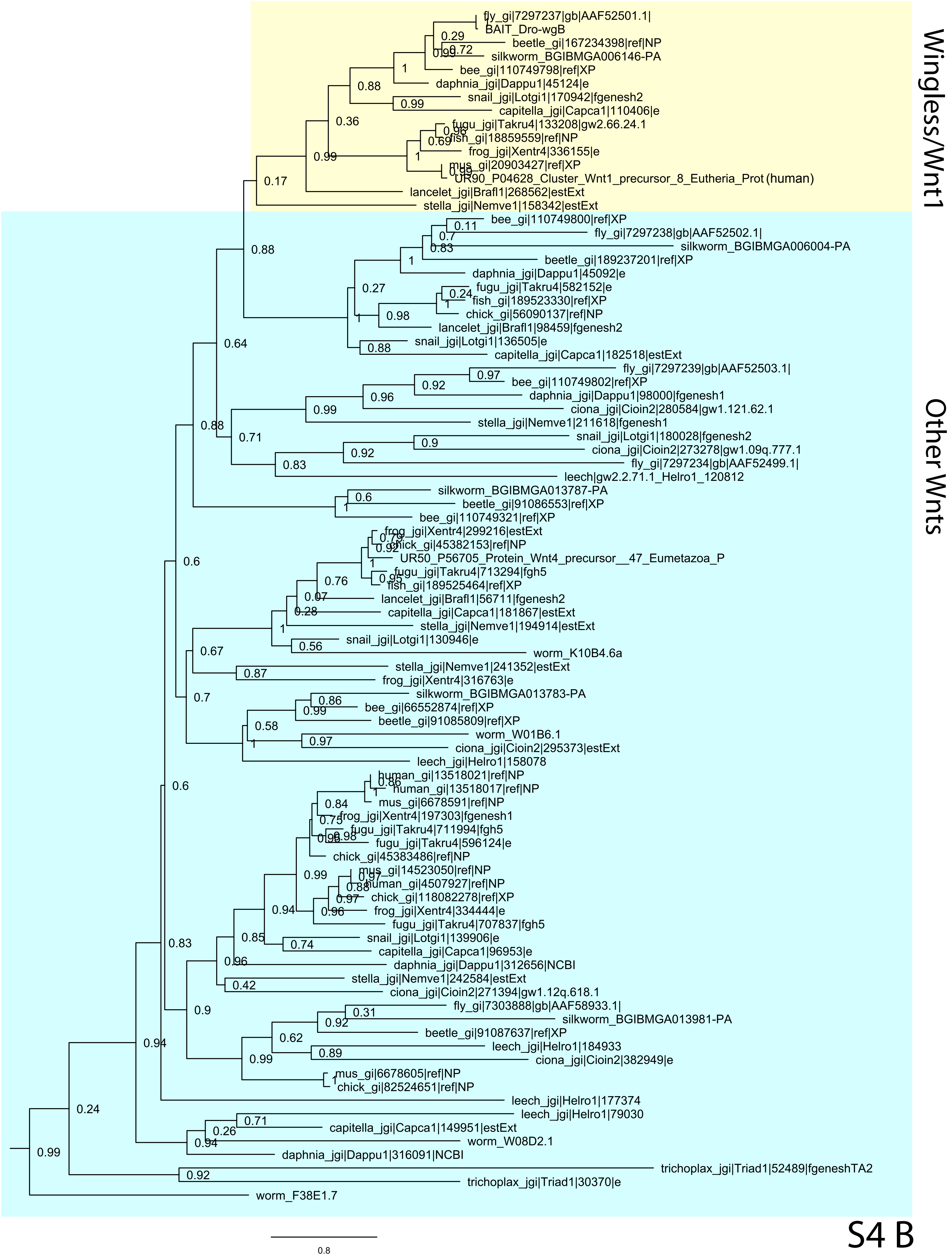
Wingless/Wnt1
Other Wnts
fly_gi|7297237|gb|AAF52501.1|
BAIT_Dro-wgB
beetle_gi|167234398|ref|NP
silkworm_BGIBMGA006146-PA
bee_gi|110749798|ref|XP
daphnia_jgi|Dappu1|45124|e
snail_jgi|Lotgi1|170942|fgenesh2
capitella_jgi|Capca1|110406|e
fugu_jgi|Takru4|133208|gw2.66.24.1
fish_gi|18859559|ref|NP
frog_jgi|Xentr4|336155|e
mus_gi|20903427|ref|XP
UR90_P04628_Cluster_Wnt1_precursor_8_Eutheria_Prot (human)
lancelet_jgi|Brafl1|268562|estExt
stella_jgi|Nemve1|158342|estExt
bee_gi|110749800|ref|XP
fly_gi|7297238|gb|AAF52502.1|
silkworm_BGIBMGA006004-PA
beetle_gi|189237201|ref|XP
daphnia_jgi|Dappu1|45092|e
fugu_jgi|Takru4|582152|e
fish_gi|189523330|ref|XP
chick_gi|56090137|ref|NP
lancelet_jgi|Brafl1|98459|fgenesh2
snail_jgi|Lotgi1|136505|e
capitella_jgi|Capca1|182518|estExt
fly_gi|7297239|gb|AAF52503.1|
bee_gi|110749802|ref|XP
daphnia_jgi|Dappu1|98000|fgenesh1
ciona_jgi|Cioin2|280584|gw1.121.62.1
stella_jgi|Nemve1|211618|fgenesh1
snail_jgi|Lotgi1|180028|fgenesh2
ciona_jgi|Cioin2|273278|gw1.09q.777.1
fly_gi|7297234|gb|AAF52499.1|
leech|gw2.2.71.1_Helro1_120812
silkworm_BGIBMGA013787-PA
beetle_gi|91086553|ref|XP
bee_gi|110749321|ref|XP
frog_jgi|Xentr4|299216|estExt
chick_gi|45382153|ref|NP
UR50_P56705_Protein_Wnt4_precursor__47_Eumetazoa_P
fugu_jgi|Takru4|713294|fgh5
fish_gi|189525464|ref|XP
lancelet_jgi|Brafl1|56711|fgenesh2
capitella_jgi|Capca1|181867|estExt
stella_jgi|Nemve1|194914|estExt
snail_jgi|Lotgi1|130946|e
worm_K10B4.6a
stella_jgi|Nemve1|241352|estExt
frog_jgi|Xentr4|316763|e
silkworm_BGIBMGA013783-PA
bee_gi|66552874|ref|XP
beetle_gi|91085809|ref|XP
worm_W01B6.1
ciona_jgi|Cioin2|295373|estExt
leech_jgi|Helro1|158078
human_gi|13518021|ref|NP
human_gi|13518017|ref|NP
mus_gi|6678591|ref|NP
frog_jgi|Xentr4|197303|fgenesh1
fugu_jgi|Takru4|711994|fgh5
fugu_jgi|Takru4|596124|e
chick_gi|45383486|ref|NP
mus_gi|14523050|ref|NP
human_gi|4507927|ref|NP
chick_gi|118082278|ref|XP
frog_jgi|Xentr4|334444|e
fugu_jgi|Takru4|707837|fgh5
snail_jgi|Lotgi1|139906|e
capitella_jgi|Capca1|96953|e
daphnia_jgi|Dappu1|312656|NCBI
stella_jgi|Nemve1|242584|estExt
ciona_jgi|Cioin2|271394|gw1.12q.618.1
fly_gi|7303888|gb|AAF58933.1|
silkworm_BGIBMGA013981-PA
beetle_gi|91087637|ref|XP
leech_jgi|Helro1|184933
ciona_jgi|Cioin2|382949|e
mus_gi|6678605|ref|NP
chick_gi|82524651|ref|NP
leech_jgi|Helro1|177374
leech_jgi|Helro1|79030
capitella_jgi|Capca1|149951|estExt
worm_W08D2.1
daphnia_jgi|Dappu1|316091|NCBI
trichoplax_jgi|Triad1|52489|fgeneshTA2
trichoplax_jgi|Triad1|30370|e
worm_F38E1.7
0.8

S4 B

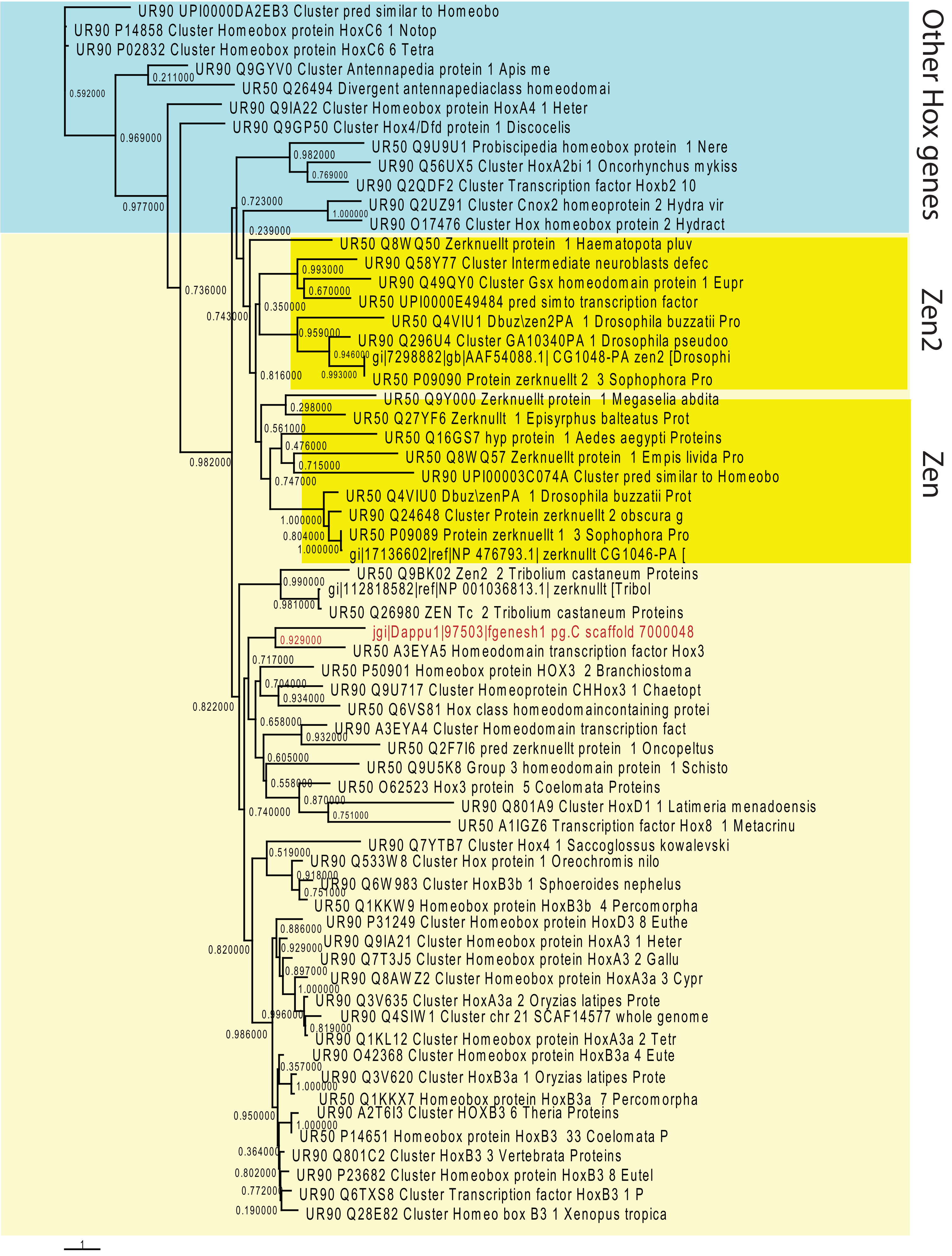
UR90 UPI0000DA2EB3 Cluster pred similar to Homeobo
UR90 P14858 Cluster Homeobox protein HoxC6 1 Notop
UR90 P02832 Cluster Homeobox protein HoxC6 6 Tetra
UR90 Q9GYV0 Cluster Antennapedia protein 1 Apis me
UR50 Q26494 Divergent antennapediaclass homeodomai
UR90 Q9IA22 Cluster Homeobox protein HoxA4 1 Heter
UR90 Q9GP50 Cluster Hox4/Dfd protein 1 Discocelis
UR50 Q9U9U1 Probiscipedia homeobox protein 1 Nere
UR90 Q56UX5 Cluster HoxA2bi 1 Oncorhynchus mykiss
UR90 Q2QDF2 Cluster Transcription factor Hoxb2 10
UR90 Q2UZ91 Cluster Cnox2 homeoprotein 2 Hydra vir
UR90 O17476 Cluster Hox homeobox protein 2 Hydract
UR50 Q8WQ50 Zerknuellt protein 1 Haematopota pluv
UR90 Q58Y77 Cluster Intermediate neuroblasts defec
UR90 Q49QY0 Cluster Gsx homeodomain protein 1 Eupr
UR50 UPI0000E49484 pred simto transcription factor
UR50 Q4VIU1 Dbuz\zen2PA 1 Drosophila buzzatii Pro
UR90 Q296U4 Cluster GA10340PA 1 Drosophila pseudoo
gi|7298882|gb|AAF54088.1| CG1048-PA zen2 [Drosophi
UR50 P09090 Protein zerknuellt 2 3 Sophophora Pro
UR50 Q9Y000 Zerknuellt protein 1 Megaselia abdita
UR50 Q27YF6 Zerknullt 1 Episyrphus balteatus Prot
UR50 Q16GS7 hyp protein 1 Aedes aegypti Proteins
UR50 Q8WQ57 Zerknuellt protein 1 Empis livida Pro
UR90 UPI00003C074A Cluster pred similar to Homeobo
UR50 Q4VIU0 Dbuz\zenPA 1 Drosophila buzzatii Prot
UR90 Q24648 Cluster Protein zerknuellt 2 obscura g
UR50 P09089 Protein zerknuellt 1 3 Sophophora Pro
gi|17136602|ref|NP 476793.1| zerknullt CG1046-PA [
UR50 Q9BK02 Zen2 2 Tribolium castaneum Proteins
gi|112818582|ref|NP 001036813.1| zerknullt [Tribol
UR50 Q26980 ZEN Tc 2 Tribolium castaneum Proteins
jgi|Dappu1|97503|fgenesh1 pg.C scaffold 7000048
UR50 A3EYA5 Homeodomain transcription factor Hox3
UR50 P50901 Homeobox protein HOX3 2 Branchiostoma
UR90 Q9U717 Cluster Homeoprotein CHHox3 1 Chaetopt
UR50 Q6VS81 Hox class homeodomaincontaining protei
UR90 A3EYA4 Cluster Homeodomain transcription fact
UR50 Q2F7I6 pred zerknuellt protein 1 Oncopeltus
UR50 Q9U5K8 Group 3 homeodomain protein 1 Schisto
UR50 O62523 Hox3 protein 5 Coelomata Proteins
UR90 Q801A9 Cluster HoxD1 1 Latimeria menadoensis
UR50 A1IGZ6 Transcription factor Hox8 1 Metacrinu
UR90 Q7YTB7 Cluster Hox4 1 Saccoglossus kowalevski
UR90 Q533W8 Cluster Hox protein 1 Oreochromis nilo
UR90 Q6W983 Cluster HoxB3b 1 Sphoeroides nephelus
UR50 Q1KKW9 Homeobox protein HoxB3b 4 Percomorpha
UR90 P31249 Cluster Homeobox protein HoxD3 8 Euthe
UR90 Q9IA21 Cluster Homeobox protein HoxA3 1 Heter
UR90 Q7T3J5 Cluster Homeobox protein HoxA3 2 Gallu
UR90 Q8AWZ2 Cluster Homeobox protein HoxA3a 3 Cypr
UR90 Q3V635 Cluster HoxA3a 2 Oryzias latipes Prote
UR90 Q4SIW1 Cluster chr 21 SCAF14577 whole genome
UR90 Q1KL12 Cluster Homeobox protein HoxA3a 2 Tetr
UR90 O42368 Cluster Homeobox protein HoxB3a 4 Eute
UR90 Q3V620 Cluster HoxB3a 1 Oryzias latipes Prote
UR50 Q1KKX7 Homeobox protein HoxB3a 7 Percomorpha
UR90 A2T6I3 Cluster HOXB3 6 Theria Proteins
UR50 P14651 Homeobox protein HoxB3 33 Coelomata P
UR90 Q801C2 Cluster HoxB3 3 Vertebrata Proteins
UR90 P23682 Cluster Homeobox protein HoxB3 8 Eutel
UR90 Q6TXS8 Cluster Transcription factor HoxB3 1 P
UR90 Q28E82 Cluster Homeo box B3 1 Xenopus tropica
Other Hox genes
Zen2
Zen
1

S5 A

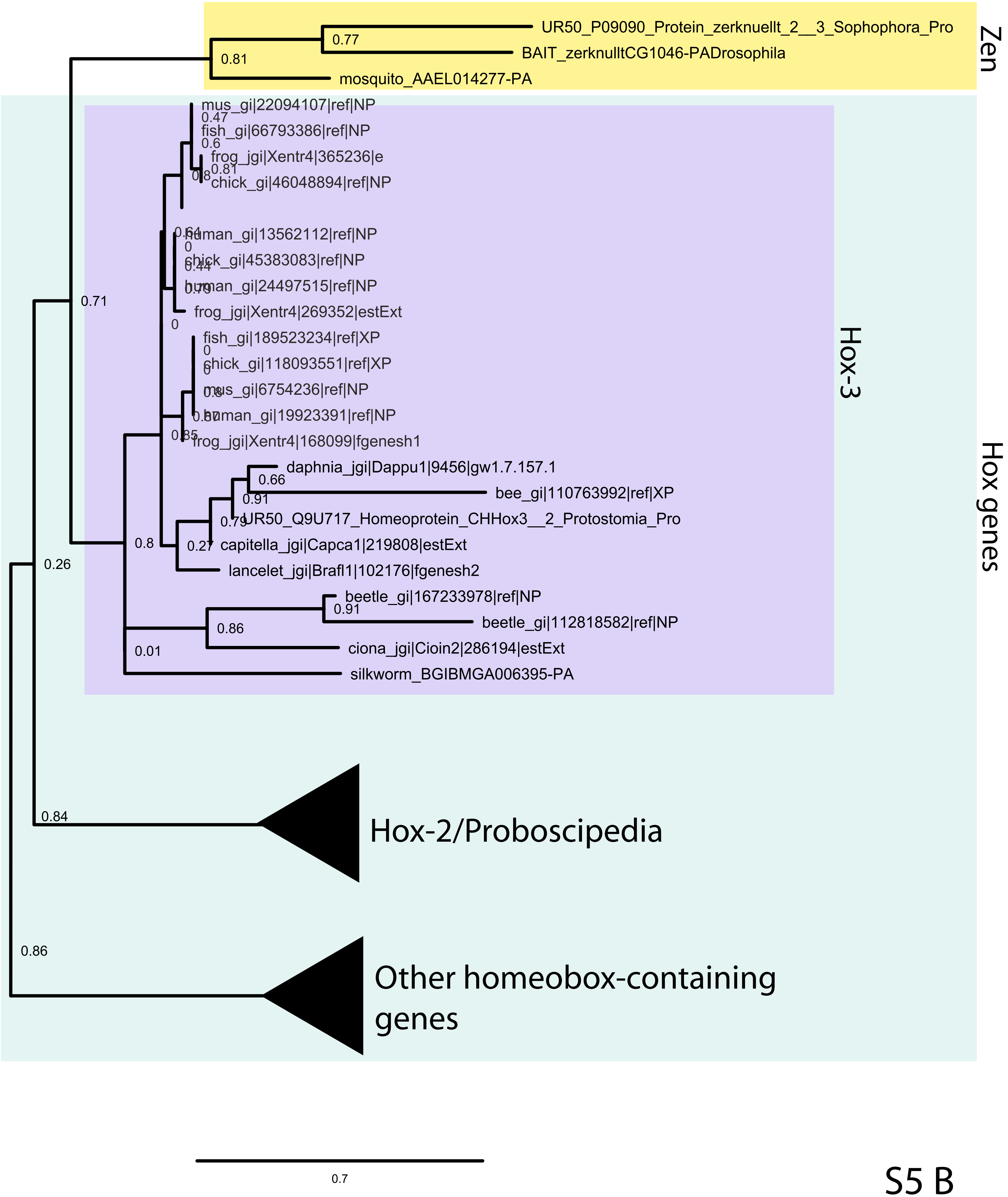

UR50_P09090_Protein_zerknuellt_2__3_Sophophora_Pro
BAIT_zerknulltCG1046-PADrosophila
mosquito_AAEL014277-PA
Zen
mus_gi|22094107|ref|NP
fish_gi|66793386|ref|NP
frog_jgi|Xentr4|365236|e
chick_gi|46048894|ref|NP
human_gi|13562112|ref|NP
chick_gi|45383083|ref|NP
human_gi|24497515|ref|NP
frog_jgi|Xentr4|269352|estExt
fish_gi|189523234|ref|XP
chick_gi|118093551|ref|XP
mus_gi|6754236|ref|NP
human_gi|19923391|ref|NP
frog_jgi|Xentr4|168099|fgenesh1
daphnia_jgi|Dappu1|9456|gw1.7.157.1
bee_gi|110763992|ref|XP
UR50_Q9U717_Homeoprotein_CHHox3__2_Protostomia_Pro
capitella_jgi|Capca1|219808|estExt
lancelet_jgi|Brafl1|102176|fgenesh2
beetle_gi|167233978|ref|NP
beetle_gi|112818582|ref|NP
ciona_jgi|Cioin2|286194|estExt
silkworm_BGIBMGA006395-PA
Hox-3
Hox genes
Hox-2/Proboscipedia
Other homeobox-containing genes
0.7

S5 B

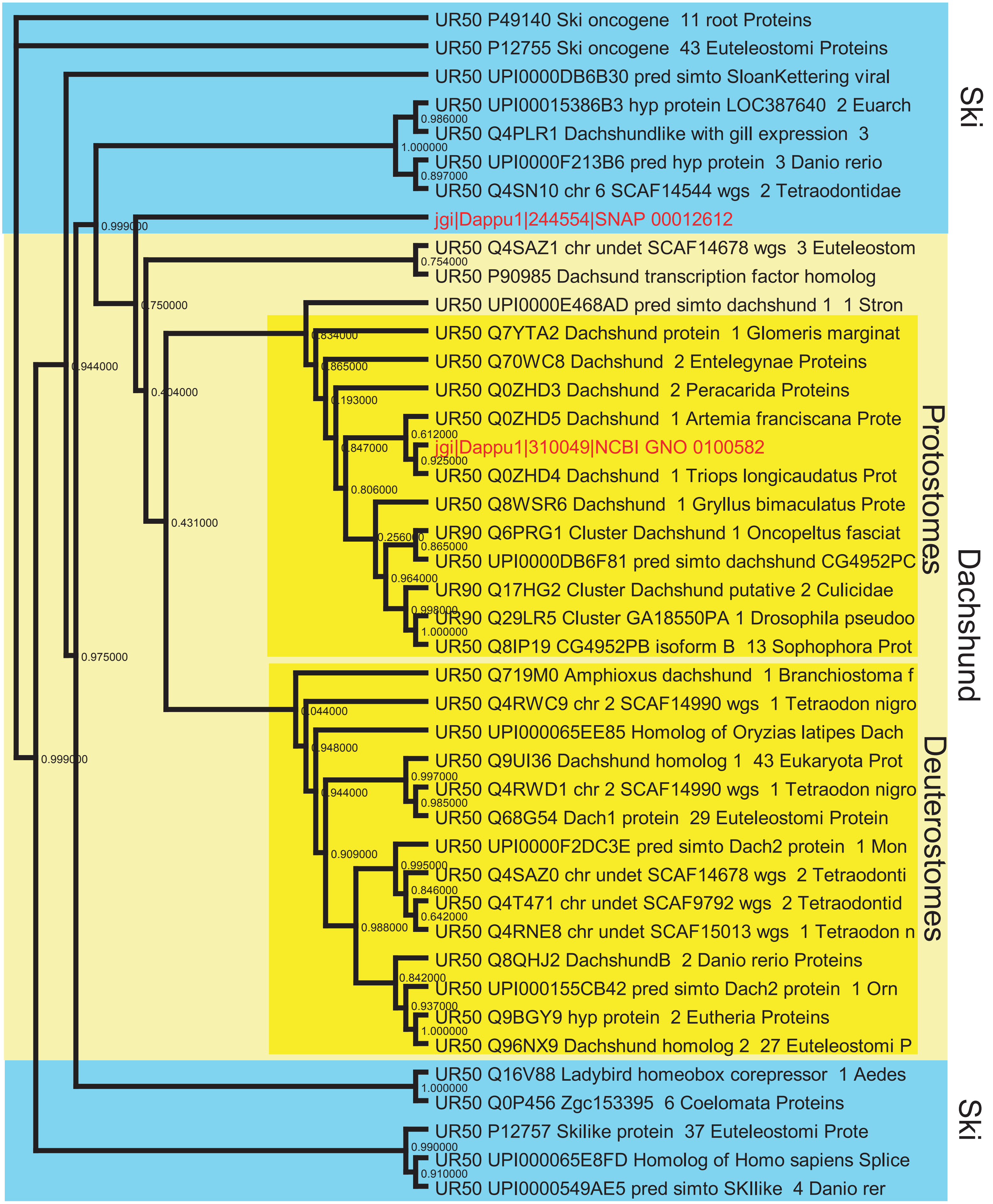
UR50 P49140 Ski oncogene 11 root Proteins
UR50 P12755 Ski oncogene 43 Euteleostomi Proteins
UR50 UPI0000DB6B30 pred simto SloanKettering viral
UR50 UPI00015386B3 hyp protein LOC387640 2 Euarch
0.986000
UR50 Q4PLR1 Dachshundlike with gill expression 3
1.000000
UR50 UPI0000F213B6 pred hyp protein 3 Danio rerio
0.897000
UR50 Q4SN10 chr 6 SCAF14544 wgs 2 Tetraodontidae
0.999000
jgi|Dappu1|244554|SNAP 00012612
UR50 Q4SAZ1 chr undet SCAF14678 wgs 3 Euteleostom
0.754000
UR50 P90985 Dachsund transcription factor homolog
0.750000
UR50 UPI0000E468AD pred simto dachshund 1 1 Stron
0.834000
UR50 Q7YTA2 Dachshund protein 1 Glomeris marginat
0.944000
0.865000
UR50 Q70WC8 Dachshund 2 Entelegynae Proteins
0.404000
0.193000
UR50 Q0ZHD3 Dachshund 2 Peracarida Proteins
UR50 Q0ZHD5 Dachshund 1 Artemia franciscana Prote
0.612000
0.847000
jgi|Dappu1|310049|NCBI GNO 0100582
0.925000
UR50 Q0ZHD4 Dachshund 1 Triops longicaudatus Prot
0.806000
UR50 Q8WSR6 Dachshund 1 Gryllus bimaculatus Prote
0.431000
0.256000
UR90 Q6PRG1 Cluster Dachshund 1 Oncopeltus fasciat
0.865000
UR50 UPI0000DB6F81 pred simto dachshund CG4952PC
0.964000
UR90 Q17HG2 Cluster Dachshund putative 2 Culicidae
0.998000
UR90 Q29LR5 Cluster GA18550PA 1 Drosophila pseudoo
1.000000
UR50 Q8IP19 CG4952PB isoform B 13 Sophophora Prot
0.975000
UR50 Q719M0 Amphioxus dachshund 1 Branchiostoma f
0.044000
UR50 Q4RWC9 chr 2 SCAF14990 wgs 1 Tetraodon nigro
UR50 UPI000065EE85 Homolog of Oryzias latipes Dach
0.999000
0.948000
UR50 Q9UI36 Dachshund homolog 1 43 Eukaryota Prot
0.997000
0.944000
UR50 Q4RWD1 chr 2 SCAF14990 wgs 1 Tetraodon nigro
0.985000
UR50 Q68G54 Dach1 protein 29 Euteleostomi Protein
UR50 UPI0000F2DC3E pred simto Dach2 protein 1 Mon
0.909000
0.995000
UR50 Q4SAZ0 chr undet SCAF14678 wgs 2 Tetraodonti
0.846000
UR50 Q4T471 chr undet SCAF9792 wgs 2 Tetraodontid
0.642000
0.988000
UR50 Q4RNE8 chr undet SCAF15013 wgs 1 Tetraodon n
UR50 Q8QHJ2 DachshundB 2 Danio rerio Proteins
0.842000
UR50 UPI000155CB42 pred simto Dach2 protein 1 Orn
0.937000
UR50 Q9BGY9 hyp protein 2 Eutheria Proteins
1.000000
UR50 Q96NX9 Dachshund homolog 2 27 Euteleostomi P
UR50 Q16V88 Ladybird homeobox corepressor 1 Aedes
1.000000
UR50 Q0P456 Zgc153395 6 Coelomata Proteins
UR50 P12757 Skilike protein 37 Euteleostomi Prote
0.990000
UR50 UPI000065E8FD Homolog of Homo sapiens Splice
0.910000
UR50 UPI0000549AE5 pred simto SKIlike 4 Danio rer
Ski
Protostomes
Dachshund
Deuterostomes
Ski

S6 A

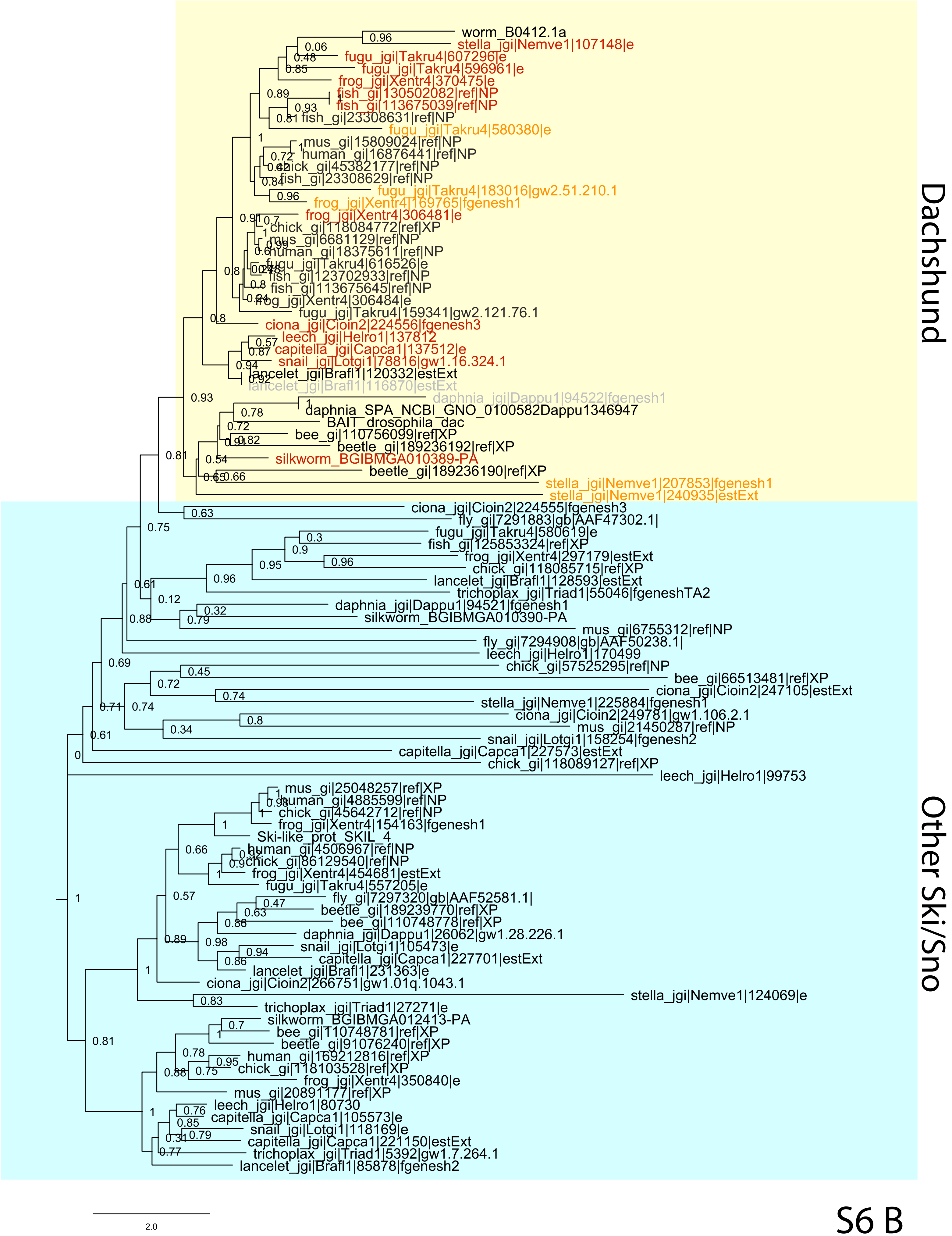
worm_B0412.1a
stella_jgi|Nemve1|107148|e
fugu_jgi|Takru4|607296|e
fugu_jgi|Takru4|596961|e
frog_jgi|Xentr4|370475|e
fish_gi|130502082|ref|NP
fish_gi|113675039|ref|NP
fish_gi|23308631|ref|NP
fugu_jgi|Takru4|580380|e
mus_gi|15809024|ref|NP
human_gi|16876441|ref|NP
chick_gi|45382177|ref|NP
fish_gi|23308629|ref|NP
fugu_jgi|Takru4|183016|gw2.51.210.1
frog_jgi|Xentr4|169765|fgenesh1
frog_jgi|Xentr4|306481|e
chick_gi|118084772|ref|XP
mus_gi|6681129|ref|NP
human_gi|18375611|ref|NP
fugu_jgi|Takru4|616526|e
fish_gi|123702933|ref|NP
fish_gi|113675645|ref|NP
frog_jgi|Xentr4|306484|e
fugu_jgi|Takru4|159341|gw2.121.76.1
ciona_jgi|Cioin2|224556|fgenesh3
leech_jgi|Helro1|137812
capitella_jgi|Capca1|137512|e
snail_jgi|Lotgi1|78816|gw1.16.324.1
lancelet_jgi|Brafl1|120332|estExt
lancelet_jgi|Brafl1|116870|estExt
daphnia_jgi|Dappu1|94522|fgenesh1
daphnia_SPA_NCBI_GNO_0100582Dappu1346947
BAIT_drosophila_dac
bee_gi|110756099|ref|XP
beetle_gi|189236192|ref|XP
silkworm_BGIBMGA010389-PA
beetle_gi|189236190|ref|XP
stella_jgi|Nemve1|207853|fgenesh1
stella_jgi|Nemve1|240935|estExt
ciona_jgi|Cioin2|224555|fgenesh3
fly_gi|7291883|gb|AAF47302.1|
fugu_jgi|Takru4|580619|e
fish_gi|125853324|ref|XP
frog_jgi|Xentr4|297179|estExt
chick_gi|118085715|ref|XP
lancelet_jgi|Brafl1|128593|estExt
trichoplax_jgi|Triad1|55046|fgeneshTA2
daphnia_jgi|Dappu1|94521|fgenesh1
silkworm_BGIBMGA010390-PA
mus_gi|6755312|ref|NP
fly_gi|7294908|gb|AAF50238.1|
leech_jgi|Helro1|170499
chick_gi|57525295|ref|NP
bee_gi|66513481|ref|XP
ciona_jgi|Cioin2|247105|estExt
stella_jgi|Nemve1|225884|fgenesh1
ciona_jgi|Cioin2|249781|gw1.106.2.1
mus_gi|21450287|ref|NP
snail_jgi|Lotgi1|158254|fgenesh2
capitella_jgi|Capca1|227573|estExt
chick_gi|118089127|ref|XP
leech_jgi|Helro1|99753
mus_gi|25048257|ref|XP
human_gi|4885599|ref|NP
chick_gi|45642712|ref|NP
frog_jgi|Xentr4|154163|fgenesh1
Ski-like_prot_SKIL_4
human_gi|4506967|ref|NP
chick_gi|86129540|ref|NP
frog_jgi|Xentr4|454681|estExt
fugu_jgi|Takru4|557205|e
fly_gi|7297320|gb|AAF52581.1|
beetle_gi|189239770|ref|XP
bee_gi|110748778|ref|XP
daphnia_jgi|Dappu1|26062|gw1.28.226.1
snail_jgi|Lotgi1|105473|e
capitella_jgi|Capca1|227701|estExt
lancelet_jgi|Brafl1|231363|e
ciona_jgi|Cioin2|266751|gw1.01q.1043.1
stella_jgi|Nemve1|124069|e
trichoplax_jgi|Triad1|27271|e
silkworm_BGIBMGA012413-PA
bee_gi|110748781|ref|XP
beetle_gi|91076240|ref|XP
human_gi|169212816|ref|XP
chick_gi|118103528|ref|XP
frog_jgi|Xentr4|350840|e
mus_gi|20891177|ref|XP
leech_jgi|Helro1|80730
capitella_jgi|Capca1|105573|e
snail_jgi|Lotgi1|118169|e
capitella_jgi|Capca1|221150|estExt
trichoplax_jgi|Triad1|5392|gw1.7.264.1
lancelet_jgi|Brafl1|85878|fgenesh2
Dachshund
Other Ski/Sno
2.0

S6 B

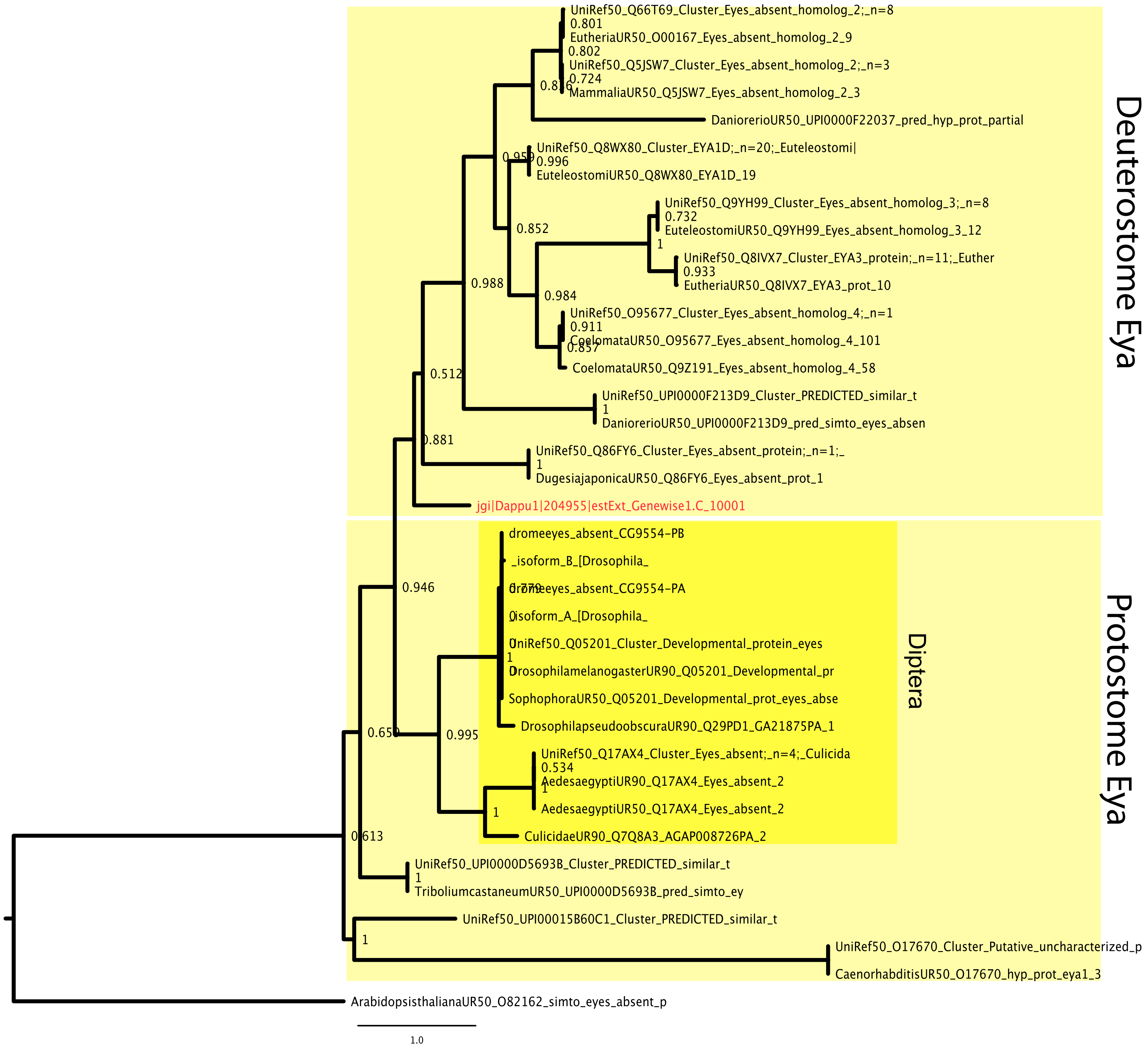
UniRef50_Q66T69_Cluster_Eyes_absent_homolog_2;_n=8
0.801
EutheriaUR50_O00167_Eyes_absent_homolog_2_9
0.802
UniRef50_Q5JSW7_Cluster_Eyes_absent_homolog_2;_n=3
0.724
MammaliaUR50_Q5JSW7_Eyes_absent_homolog_2_3
DaniorerioUR50_UPI0000F22037_pred_hyp_prot_partial
UniRef50_Q8WX80_Cluster_EYA1D;_n=20;_Euteleostomi|
0.996
EuteleostomiUR50_Q8WX80_EYA1D_19
0.852
UniRef50_Q9YH99_Cluster_Eyes_absent_homolog_3;_n=8
0.732
EuteleostomiUR50_Q9YH99_Eyes_absent_homolog_3_12
1
UniRef50_Q8IVX7_Cluster_EYA3_protein;_n=11;_Euther
0.933
EutheriaUR50_Q8IVX7_EYA3_prot_10
0.988
0.984
UniRef50_O95677_Cluster_Eyes_absent_homolog_4;_n=1
0.911
CoelomataUR50_O95677_Eyes_absent_homolog_4_101
CoelomataUR50_Q9Z191_Eyes_absent_homolog_4_58
0.512
UniRef50_UPI0000F213D9_Cluster_PREDICTED_similar_t
1
DaniorerioUR50_UPI0000F213D9_pred_simto_eyes_absen
UniRef50_Q86FY6_Cluster_Eyes_absent_protein;_n=1;_
1
DugesiajaponicaUR50_Q86FY6_Eyes_absent_prot_1
jgi|Dappu1|204955|estExt_Genewise1.C_10001
dromeeyes_absent_CG9554-PB
_isoform_B_[Drosophila_
0.946
dromeeyes_absent_CG9554-PA
isoform_A_[Drosophila_
UniRef50_Q05201_Cluster_Developmental_protein_eyes
1
DrosophilamelanogasterUR90_Q05201_Developmental_pr
SophophoraUR50_Q05201_Developmental_prot_eyes_abse
DrosophilapseudoobscuraUR90_Q29PD1_GA21875PA_1
0.995
UniRef50_Q17AX4_Cluster_Eyes_absent;_n=4;_Culicida
0.534
AedesaegyptiUR90_Q17AX4_Eyes_absent_2
1
AedesaegyptiUR50_Q17AX4_Eyes_absent_2
1
CulicidaeUR90_Q7Q8A3_AGAP008726PA_2
UniRef50_UPI0000D5693B_Cluster_PREDICTED_similar_t
1
TriboliumcastaneumUR50_UPI0000D5693B_pred_simto_ey
UniRef50_UPI00015B60C1_Cluster_PREDICTED_similar_t
1
UniRef50_O17670_Cluster_Putative_uncharacterized_p
CaenorhabditisUR50_O17670_hyp_prot_eya1_3
ArabidopsisthalianaUR50_O82162_simto_eyes_absent_p
1.0
Deuterostome Eya
Diptera
Protostome Eya

S7 A

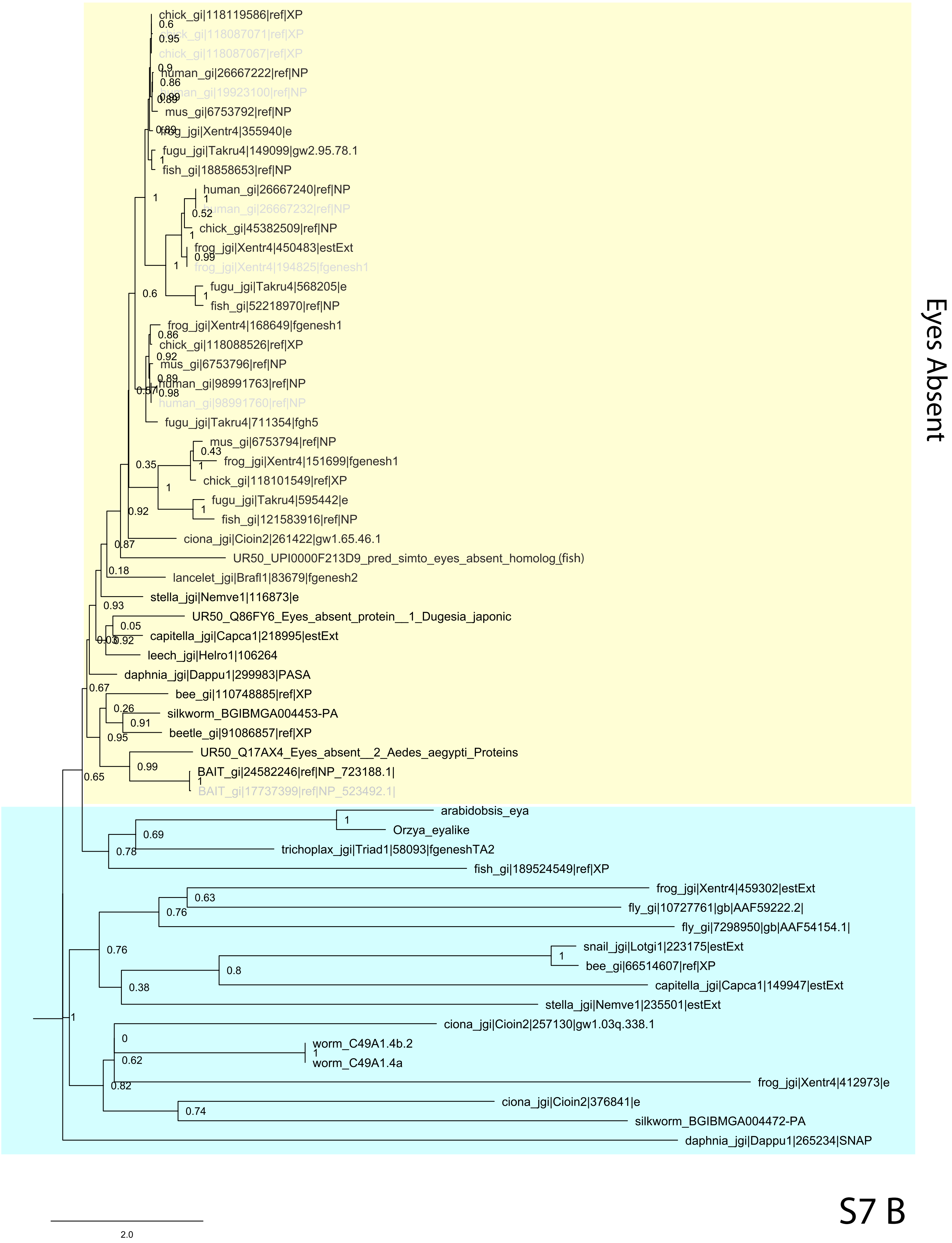
Eyes Absent
chick_gi|118119586|ref|XP
chick_gi|118087071|ref|XP
chick_gi|118087067|ref|XP
human_gi|26667222|ref|NP
human_gi|19923100|ref|NP
mus_gi|6753792|ref|NP
frog_jgi|Xentr4|355940|e
fugu_jgi|Takru4|149099|gw2.95.78.1
fish_gi|18858653|ref|NP
human_gi|26667240|ref|NP
human_gi|26667232|ref|NP
chick_gi|45382509|ref|NP
frog_jgi|Xentr4|450483|estExt
frog_jgi|Xentr4|194825|fgenesh1
fugu_jgi|Takru4|568205|e
fish_gi|52218970|ref|NP
frog_jgi|Xentr4|168649|fgenesh1
chick_gi|118088526|ref|XP
mus_gi|6753796|ref|NP
human_gi|98991763|ref|NP
human_gi|98991760|ref|NP
fugu_jgi|Takru4|711354|fgh5
mus_gi|6753794|ref|NP
frog_jgi|Xentr4|151699|fgenesh1
chick_gi|118101549|ref|XP
fugu_jgi|Takru4|595442|e
fish_gi|121583916|ref|NP
ciona_jgi|Cioin2|261422|gw1.65.46.1
UR50_UPI0000F213D9_pred_simto_eyes_absent_homolog_(fish)
lancelet_jgi|Brafl1|83679|fgenesh2
stella_jgi|Nemve1|116873|e
UR50_Q86FY6_Eyes_absent_protein__1_Dugesia_japonic
capitella_jgi|Capca1|218995|estExt
leech_jgi|Helro1|106264
daphnia_jgi|Dappu1|299983|PASA
bee_gi|110748885|ref|XP
silkworm_BGIBMGA004453-PA
beetle_gi|91086857|ref|XP
UR50_Q17AX4_Eyes_absent__2_Aedes_aegypti_Proteins
BAIT_gi|24582246|ref|NP_723188.1|
BAIT_gi|17737399|ref|NP_523492.1|
arabidobsis_eya
Orzya_eyalike
trichoplax_jgi|Triad1|58093|fgeneshTA2
fish_gi|189524549|ref|XP
frog_jgi|Xentr4|459302|estExt
fly_gi|10727761|gb|AAF59222.2|
fly_gi|7298950|gb|AAF54154.1|
snail_jgi|Lotgi1|223175|estExt
bee_gi|66514607|ref|XP
capitella_jgi|Capca1|149947|estExt
stella_jgi|Nemve1|235501|estExt
ciona_jgi|Cioin2|257130|gw1.03q.338.1
worm_C49A1.4b.2
worm_C49A1.4a
frog_jgi|Xentr4|412973|e
ciona_jgi|Cioin2|376841|e
silkworm_BGIBMGA004472-PA
daphnia_jgi|Dappu1|265234|SNAP
2.0

S7 B

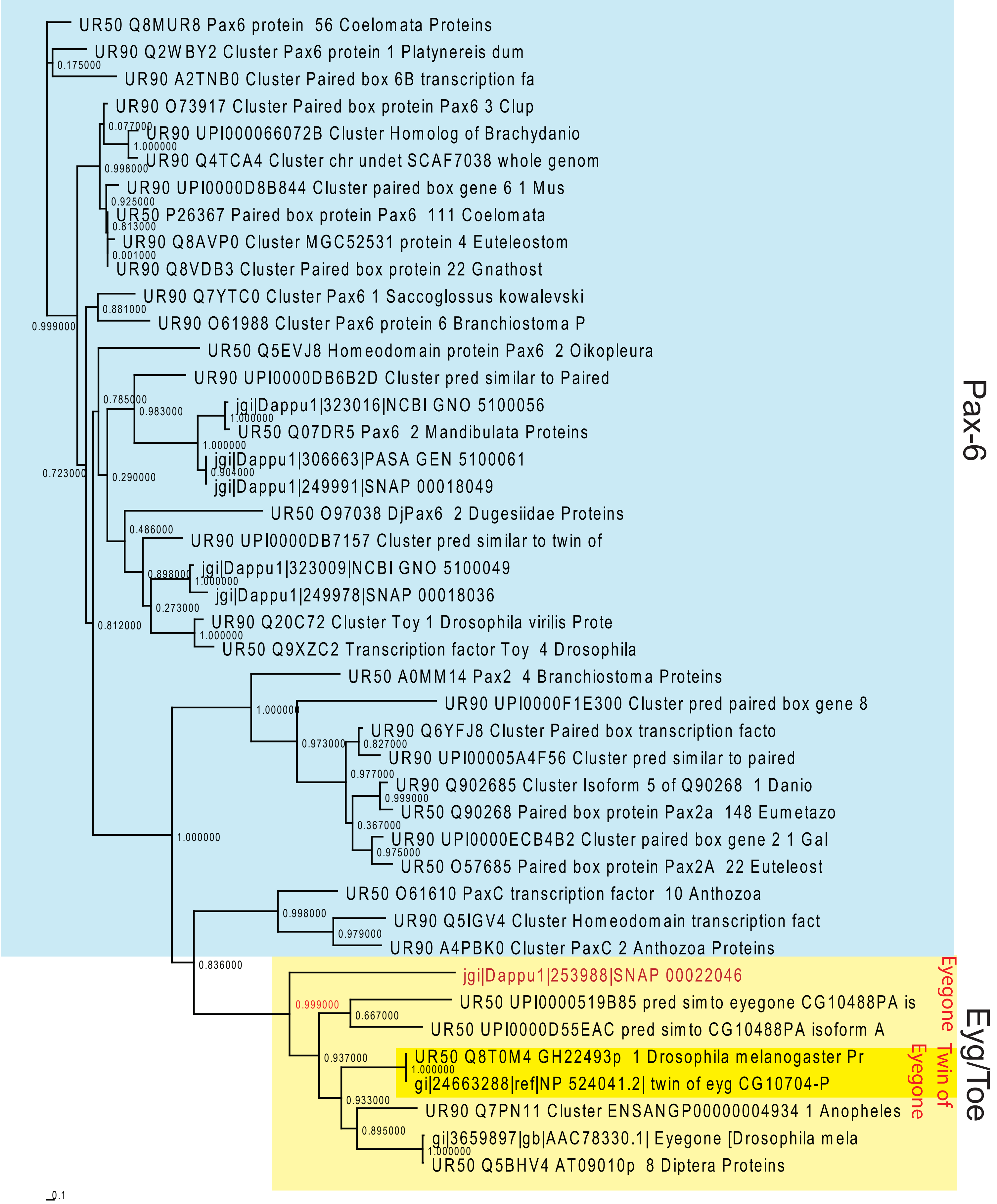
UR50 Q8MUR8 Pax6 protein 56 Coelomata Proteins
UR90 Q2WBY2 Cluster Pax6 protein 1 Platynereis dum
UR90 A2TNB0 Cluster Paired box 6B transcription fa
UR90 O73917 Cluster Paired box protein Pax6 3 Clup
UR90 UPI000066072B Cluster Homolog of Brachydanio
UR90 Q4TCA4 Cluster chr undet SCAF7038 whole genom
UR90 UPI0000D8B844 Cluster paired box gene 6 1 Mus
UR50 P26367 Paired box protein Pax6 111 Coelomata
UR90 Q8AVP0 Cluster MGC52531 protein 4 Euteleostom
UR90 Q8VDB3 Cluster Paired box protein 22 Gnathost
UR90 Q7YTC0 Cluster Pax6 1 Saccoglossus kowalevski
UR90 O61988 Cluster Pax6 protein 6 Branchiostoma P
UR50 Q5EVJ8 Homeodomain protein Pax6 2 Oikopleura
UR90 UPI0000DB6B2D Cluster pred similar to Paired
jgi|Dappu1|323016|NCBI GNO 5100056
UR50 Q07DR5 Pax6 2 Mandibulata Proteins
jgi|Dappu1|306663|PASA GEN 5100061
jgi|Dappu1|249991|SNAP 00018049
UR50 O97038 DjPax6 2 Dugesiidae Proteins
UR90 UPI0000DB7157 Cluster pred similar to twin of
jgi|Dappu1|323009|NCBI GNO 5100049
jgi|Dappu1|249978|SNAP 00018036
UR90 Q20C72 Cluster Toy 1 Drosophila virilis Prote
UR50 Q9XZC2 Transcription factor Toy 4 Drosophila
UR50 A0MM14 Pax2 4 Branchiostoma Proteins
UR90 UPI0000F1E300 Cluster pred paired box gene 8
UR90 Q6YFJ8 Cluster Paired box transcription facto
UR90 UPI00005A4F56 Cluster pred similar to paired
UR90 Q902685 Cluster Isoform 5 of Q90268 1 Danio
UR50 Q90268 Paired box protein Pax2a 148 Eumetazo
UR90 UPI0000ECB4B2 Cluster paired box gene 2 1 Gal
UR50 O57685 Paired box protein Pax2A 22 Euteleost
UR50 O61610 PaxC transcription factor 10 Anthozoa
UR90 Q5IGV4 Cluster Homeodomain transcription fact
UR90 A4PBK0 Cluster PaxC 2 Anthozoa Proteins
jgi|Dappu1|253988|SNAP 00022046
UR50 UPI0000519B85 pred sim to eyegone CG10488PA is
UR50 UPI0000D55EAC pred sim to CG10488PA isoform A
UR50 Q8T0M4 GH22493p 1 Drosophila melanogaster Pr
gi|24663288|ref|NP 524041.2| twin of eyg CG10704-P
UR90 Q7PN11 Cluster ENSANGP00000004934 1 Anopheles
gi|3659897|gb|AAC78330.1| Eyegone [Drosophila mela
UR50 Q5BHV4 AT09010p 8 Diptera Proteins
Pax-6
Eyg/Toe
Eyegone
Twin of Eyegone
0.1

S8 A

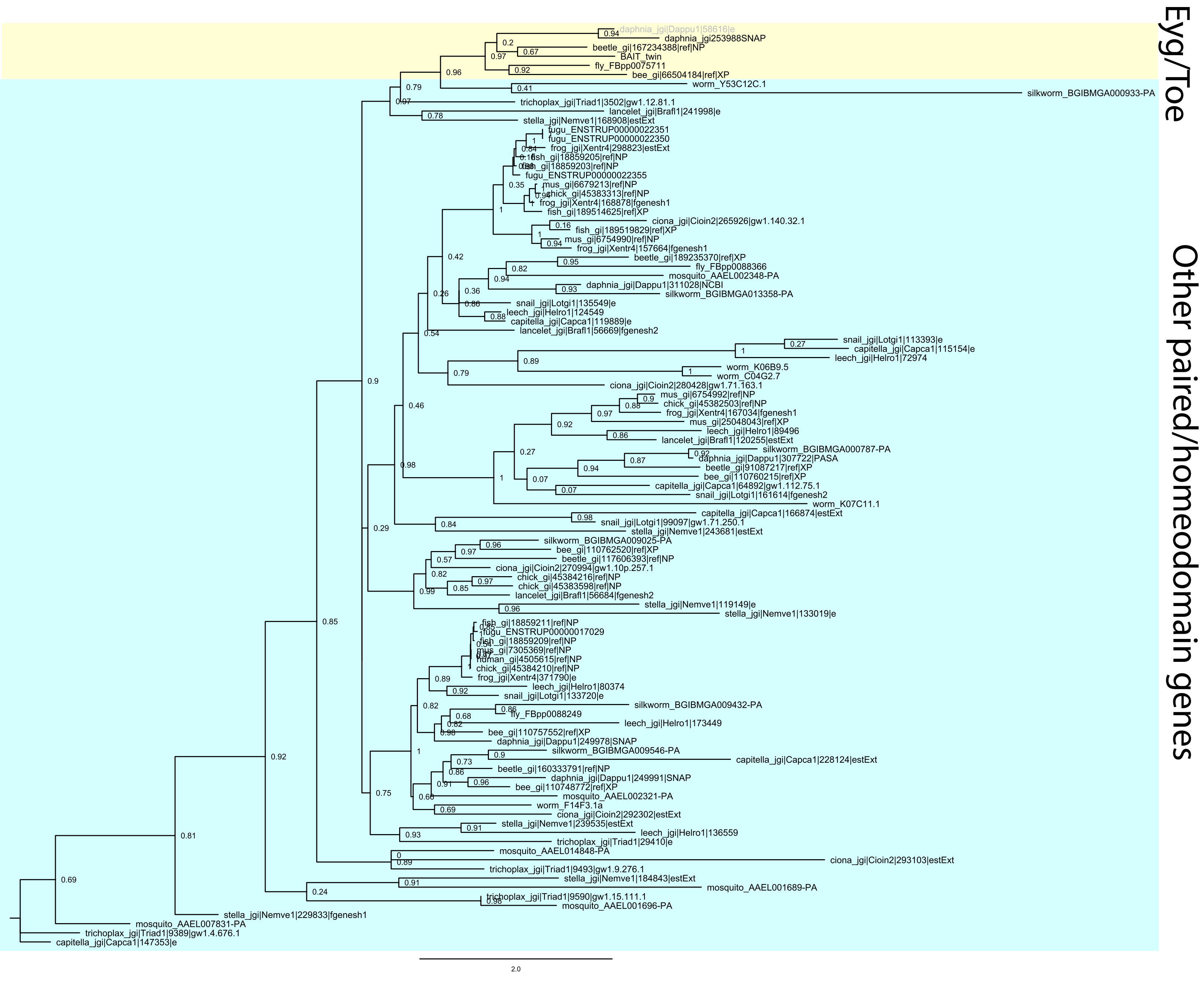
Eyg/Toe
Other paired/homeodomain genes
daphnia_jgi|Dappu1|58616|e
daphnia_jgi253988SNAP
beetle_gi|167234388|ref|NP
BAIT_twin
fly_FBpp0075711
bee_gi|66504184|ref|XP
worm_Y53C12C.1
silkworm_BGIBMGA000933-PA
trichoplax_jgi|Triad1|3502|gw1.12.81.1
lancelet_jgi|Brafl1|241998|e
stella_jgi|Nemve1|168908|estExt
fugu_ENSTRUP00000022351
fugu_ENSTRUP00000022350
frog_jgi|Xentr4|298823|estExt
fish_gi|18859205|ref|NP
fish_gi|18859203|ref|NP
fugu_ENSTRUP00000022355
mus_gi|6679213|ref|NP
chick_gi|45383313|ref|NP
frog_jgi|Xentr4|168878|fgenesh1
fish_gi|189514625|ref|XP
ciona_jgi|Cioin2|265926|gw1.140.32.1
fish_gi|189519829|ref|XP
mus_gi|6754990|ref|NP
frog_jgi|Xentr4|157664|fgenesh1
beetle_gi|189235370|ref|XP
fly_FBpp0088366
mosquito_AAEL002348-PA
daphnia_jgi|Dappu1|311028|NCBI
silkworm_BGIBMGA013358-PA
snail_jgi|Lotgi1|135549|e
leech_jgi|Helro1|124549
capitella_jgi|Capca1|119889|e
lancelet_jgi|Brafl1|56669|fgenesh2
snail_jgi|Lotgi1|113393|e
capitella_jgi|Capca1|115154|e
leech_jgi|Helro1|72974
worm_K06B9.5
worm_C04G2.7
ciona_jgi|Cioin2|280428|gw1.71.163.1
mus_gi|6754992|ref|NP
chick_gi|45382503|ref|NP
frog_jgi|Xentr4|167034|fgenesh1
mus_gi|25048043|ref|XP
leech_jgi|Helro1|89496
lancelet_jgi|Brafl1|120255|estExt
silkworm_BGIBMGA000787-PA
daphnia_jgi|Dappu1|307722|PASA
beetle_gi|91087217|ref|XP
bee_gi|110760215|ref|XP
capitella_jgi|Capca1|64892|gw1.112.75.1
snail_jgi|Lotgi1|161614|fgenesh2
worm_K07C11.1
capitella_jgi|Capca1|166874|estExt
snail_jgi|Lotgi1|99097|gw1.71.250.1
stella_jgi|Nemve1|243681|estExt
silkworm_BGIBMGA009025-PA
bee_gi|110762520|ref|XP
beetle_gi|117606393|ref|NP
ciona_jgi|Cioin2|270994|gw1.10p.257.1
chick_gi|45384216|ref|NP
chick_gi|45383598|ref|NP
lancelet_jgi|Brafl1|56684|fgenesh2
stella_jgi|Nemve1|119149|e
stella_jgi|Nemve1|133019|e
fish_gi|18859211|ref|NP
fugu_ENSTRUP00000017029
fish_gi|18859209|ref|NP
mus_gi|7305369|ref|NP
human_gi|4505615|ref|NP
chick_gi|45384210|ref|NP
frog_jgi|Xentr4|371790|e
leech_jgi|Helro1|80374
snail_jgi|Lotgi1|133720|e
silkworm_BGIBMGA009432-PA
fly_FBpp0088249
leech_jgi|Helro1|173449
bee_gi|110757552|ref|XP
daphnia_jgi|Dappu1|249978|SNAP
silkworm_BGIBMGA009546-PA
capitella_jgi|Capca1|228124|estExt
beetle_gi|160333791|ref|NP
daphnia_jgi|Dappu1|249991|SNAP
bee_gi|110748772|ref|XP
mosquito_AAEL002321-PA
worm_F14F3.1a
ciona_jgi|Cioin2|292302|estExt
stella_jgi|Nemve1|239535|estExt
leech_jgi|Helro1|136559
trichoplax_jgi|Triad1|29410|e
mosquito_AAEL014848-PA
ciona_jgi|Cioin2|293103|estExt
trichoplax_jgi|Triad1|9493|gw1.9.276.1
stella_jgi|Nemve1|184843|estExt
mosquito_AAEL001689-PA
trichoplax_jgi|Triad1|9590|gw1.15.111.1
mosquito_AAEL001696-PA
stella_jgi|Nemve1|229833|fgenesh1
mosquito_AAEL007831-PA
trichoplax_jgi|Triad1|9389|gw1.4.676.1
capitella_jgi|Capca1|147353|e
2.0

S8 B

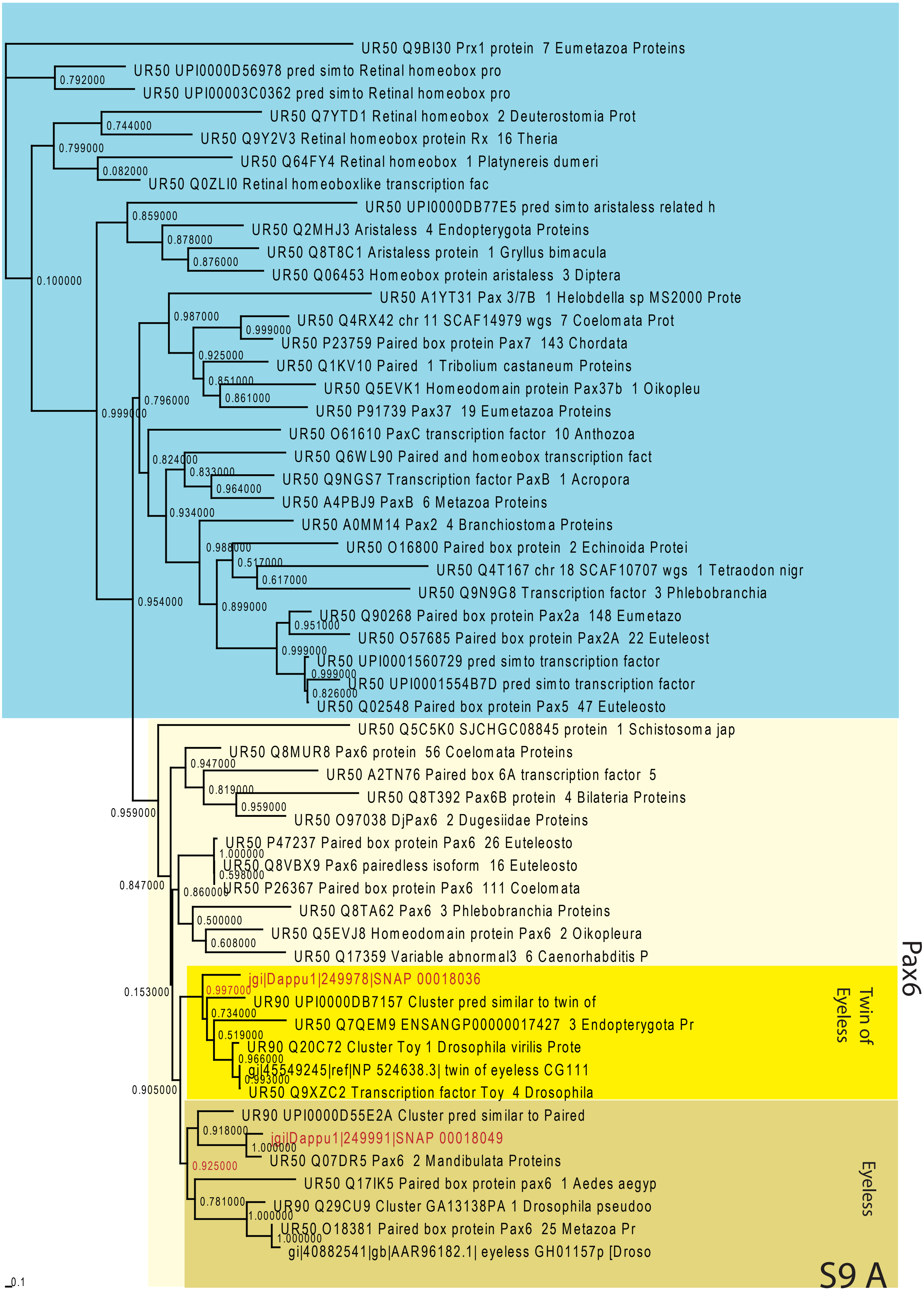

UR50 Q9BI30 Prx1 protein 7 Eumetazoa Proteins
0.792000
UR50 UPI0000D56978 pred simto Retinal homeobox pro
UR50 UPI00003C0362 pred simto Retinal homeobox pro
0.744000
UR50 Q7YTD1 Retinal homeobox 2 Deuterostomia Prot
0.799000
UR50 Q9Y2V3 Retinal homeobox protein Rx 16 Theria
0.082000
UR50 Q64FY4 Retinal homeobox 1 Platynereis dumeri
UR50 Q0ZLI0 Retinal homeoboxlike transcription fac
0.100000
0.859000
UR50 UPI0000DB77E5 pred simto aristaless related h
0.878000
UR50 Q2MHJ3 Aristaless 4 Endopterygota Proteins
0.876000
UR50 Q8T8C1 Aristaless protein 1 Gryllus bimacula
UR50 Q06453 Homeobox protein aristaless 3 Diptera
0.987000
UR50 A1YT31 Pax 3/7B 1 Helobdella sp MS2000 Prote
0.999000
UR50 Q4RX42 chr 11 SCAF14979 wgs 7 Coelomata Prot
UR50 P23759 Paired box protein Pax7 143 Chordata
0.925000
UR50 Q1KV10 Paired 1 Tribolium castaneum Proteins
0.851000
0.861000
UR50 Q5EVK1 Homeodomain protein Pax37b 1 Oikopleu
0.796000
0.999000
UR50 P91739 Pax37 19 Eumetazoa Proteins
UR50 O61610 PaxC transcription factor 10 Anthozoa
0.824000
UR50 Q6WL90 Paired and homeobox transcription fact
0.833000
UR50 Q9NGS7 Transcription factor PaxB 1 Acropora
0.964000
UR50 A4PBJ9 PaxB 6 Metazoa Proteins
0.934000
UR50 A0MM14 Pax2 4 Branchiostoma Proteins
0.988000
UR50 O16800 Paired box protein 2 Echinoida Protei
0.517000
UR50 Q4T167 chr 18 SCAF10707 wgs 1 Tetraodon nigr
0.617000
UR50 Q9N9G8 Transcription factor 3 Phlebobranchia
0.954000
0.899000
UR50 Q90268 Paired box protein Pax2a 148 Eumetazo
0.951000
UR50 O57685 Paired box protein Pax2A 22 Euteleost
0.999000
UR50 UPI0001560729 pred simto transcription factor
0.999000
UR50 UPI0001554B7D pred simto transcription factor
0.826000
UR50 Q02548 Paired box protein Pax5 47 Euteleosto
UR50 Q5C5K0 SJCHGC08845 protein 1 Schistosoma jap
UR50 Q8MUR8 Pax6 protein 56 Coelomata Proteins
0.947000
UR50 A2TN76 Paired box 6A transcription factor 5
0.819000
UR50 Q8T392 Pax6B protein 4 Bilateria Proteins
0.959000
0.959000
UR50 O97038 DjPax6 2 Dugesiidae Proteins
UR50 P47237 Paired box protein Pax6 26 Euteleosto
1.000000
UR50 Q8VBX9 Pax6 pairedless isoform 16 Euteleosto
0.598000
0.847000
0.860000
UR50 P26367 Paired box protein Pax6 111 Coelomata
0.500000
UR50 Q8TA62 Pax6 3 Phlebobranchia Proteins
UR50 Q5EVJ8 Homeodomain protein Pax6 2 Oikopleura
0.608000
UR50 Q17359 Variable abnormal3 6 Caenorhabditis P
Pax6
jgi|Dappu1|249978|SNAP 00018036
0.997000
0.153000
UR90 UPI0000DB7157 Cluster pred similar to twin of
0.734000
Twin of Eyeless
UR50 Q7QEM9 ENSANGP00000017427 3 Endopterygota Pr
0.519000
UR90 Q20C72 Cluster Toy 1 Drosophila virilis Prote
0.966000
gi|45549245|ref|NP 524638.3| twin of eyeless CG111
0.993000
0.905000
UR50 Q9XZC2 Transcription factor Toy 4 Drosophila
UR90 UPI0000D55E2A Cluster pred similar to Paired
0.918000
jgi|Dappu1|249991|SNAP 00018049
1.000000
UR50 Q07DR5 Pax6 2 Mandibulata Proteins
0.925000
UR50 Q17IK5 Paired box protein pax6 1 Aedes aegyp
Eyeless
0.781000
UR90 Q29CU9 Cluster GA13138PA 1 Drosophila pseudoo
1.000000
UR50 O18381 Paired box protein Pax6 25 Metazoa Pr
1.000000
gi|40882541|gb|AAR96182.1| eyeless GH01157p [Droso
0.1
S9 A

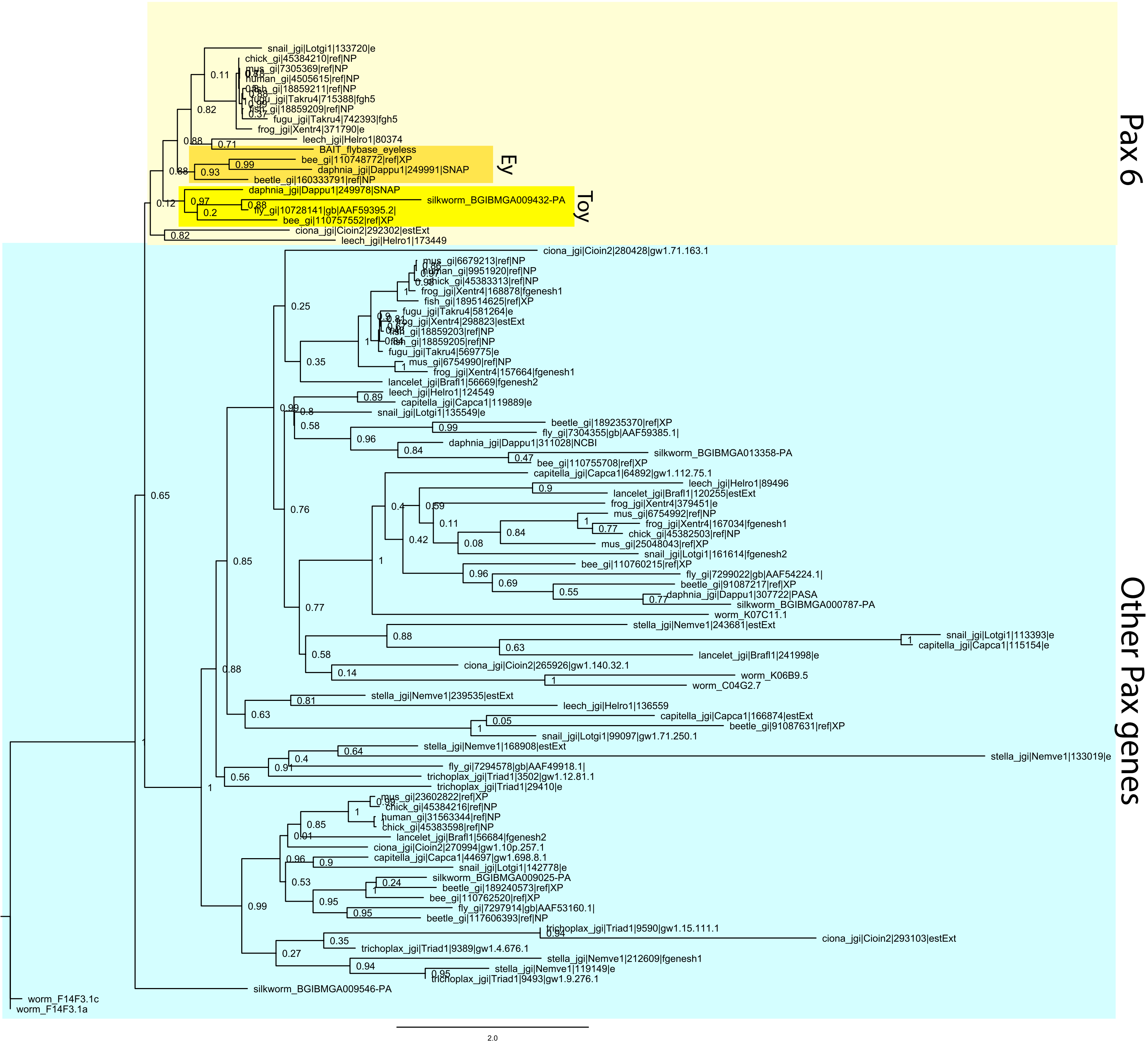
Pax 6
Ey
Toy
Other Pax genes
snail_jgi|Lotgi1|133720|e
chick_gi|45384210|ref|NP
mus_gi|7305369|ref|NP
human_gi|4505615|ref|NP
fish_gi|18859211|ref|NP
fugu_jgi|Takru4|715388|fgh5
fish_gi|18859209|ref|NP
fugu_jgi|Takru4|742393|fgh5
frog_jgi|Xentr4|371790|e
leech_jgi|Helro1|80374
BAIT_flybase_eyeless
bee_gi|110748772|ref|XP
daphnia_jgi|Dappu1|249991|SNAP
beetle_gi|160333791|ref|NP
daphnia_jgi|Dappu1|249978|SNAP
silkworm_BGIBMGA009432-PA
fly_gi|10728141|gb|AAF59395.2|
bee_gi|110757552|ref|XP
ciona_jgi|Cioin2|292302|estExt
leech_jgi|Helro1|173449
ciona_jgi|Cioin2|280428|gw1.71.163.1
mus_gi|6679213|ref|NP
human_gi|9951920|ref|NP
chick_gi|45383313|ref|NP
frog_jgi|Xentr4|168878|fgenesh1
fish_gi|189514625|ref|XP
fugu_jgi|Takru4|581264|e
frog_jgi|Xentr4|298823|estExt
fish_gi|18859203|ref|NP
fish_gi|18859205|ref|NP
fugu_jgi|Takru4|569775|e
mus_gi|6754990|ref|NP
frog_jgi|Xentr4|157664|fgenesh1
lancelet_jgi|Brafl1|56669|fgenesh2
leech_jgi|Helro1|124549
capitella_jgi|Capca1|119889|e
snail_jgi|Lotgi1|135549|e
beetle_gi|189235370|ref|XP
fly_gi|7304355|gb|AAF59385.1|
daphnia_jgi|Dappu1|311028|NCBI
silkworm_BGIBMGA013358-PA
bee_gi|110755708|ref|XP
capitella_jgi|Capca1|64892|gw1.112.75.1
leech_jgi|Helro1|89496
lancelet_jgi|Brafl1|120255|estExt
frog_jgi|Xentr4|379451|e
mus_gi|6754992|ref|NP
frog_jgi|Xentr4|167034|fgenesh1
chick_gi|45382503|ref|NP
mus_gi|25048043|ref|XP
snail_jgi|Lotgi1|161614|fgenesh2
bee_gi|110760215|ref|XP
fly_gi|7299022|gb|AAF54224.1|
beetle_gi|91087217|ref|XP
daphnia_jgi|Dappu1|307722|PASA
silkworm_BGIBMGA000787-PA
worm_K07C11.1
stella_jgi|Nemve1|243681|estExt
snail_jgi|Lotgi1|113393|e
capitella_jgi|Capca1|115154|e
lancelet_jgi|Brafl1|241998|e
ciona_jgi|Cioin2|265926|gw1.140.32.1
worm_K06B9.5
worm_C04G2.7
stella_jgi|Nemve1|239535|estExt
leech_jgi|Helro1|136559
capitella_jgi|Capca1|166874|estExt
beetle_gi|91087631|ref|XP
snail_jgi|Lotgi1|99097|gw1.71.250.1
stella_jgi|Nemve1|168908|estExt
stella_jgi|Nemve1|133019|e
fly_gi|7294578|gb|AAF49918.1|
trichoplax_jgi|Triad1|3502|gw1.12.81.1
trichoplax_jgi|Triad1|29410|e
mus_gi|23602822|ref|XP
chick_gi|45384216|ref|NP
human_gi|31563344|ref|NP
chick_gi|45383598|ref|NP
lancelet_jgi|Brafl1|56684|fgenesh2
ciona_jgi|Cioin2|270994|gw1.10p.257.1
capitella_jgi|Capca1|44697|gw1.698.8.1
snail_jgi|Lotgi1|142778|e
silkworm_BGIBMGA009025-PA
beetle_gi|189240573|ref|XP
bee_gi|110762520|ref|XP
fly_gi|7297914|gb|AAF53160.1|
beetle_gi|117606393|ref|NP
trichoplax_jgi|Triad1|9590|gw1.15.111.1
ciona_jgi|Cioin2|293103|estExt
trichoplax_jgi|Triad1|9389|gw1.4.676.1
stella_jgi|Nemve1|212609|fgenesh1
stella_jgi|Nemve1|119149|e
trichoplax_jgi|Triad1|9493|gw1.9.276.1
silkworm_BGIBMGA009546-PA
worm_F14F3.1c
worm_F14F3.1a
2.0

S9 B-1

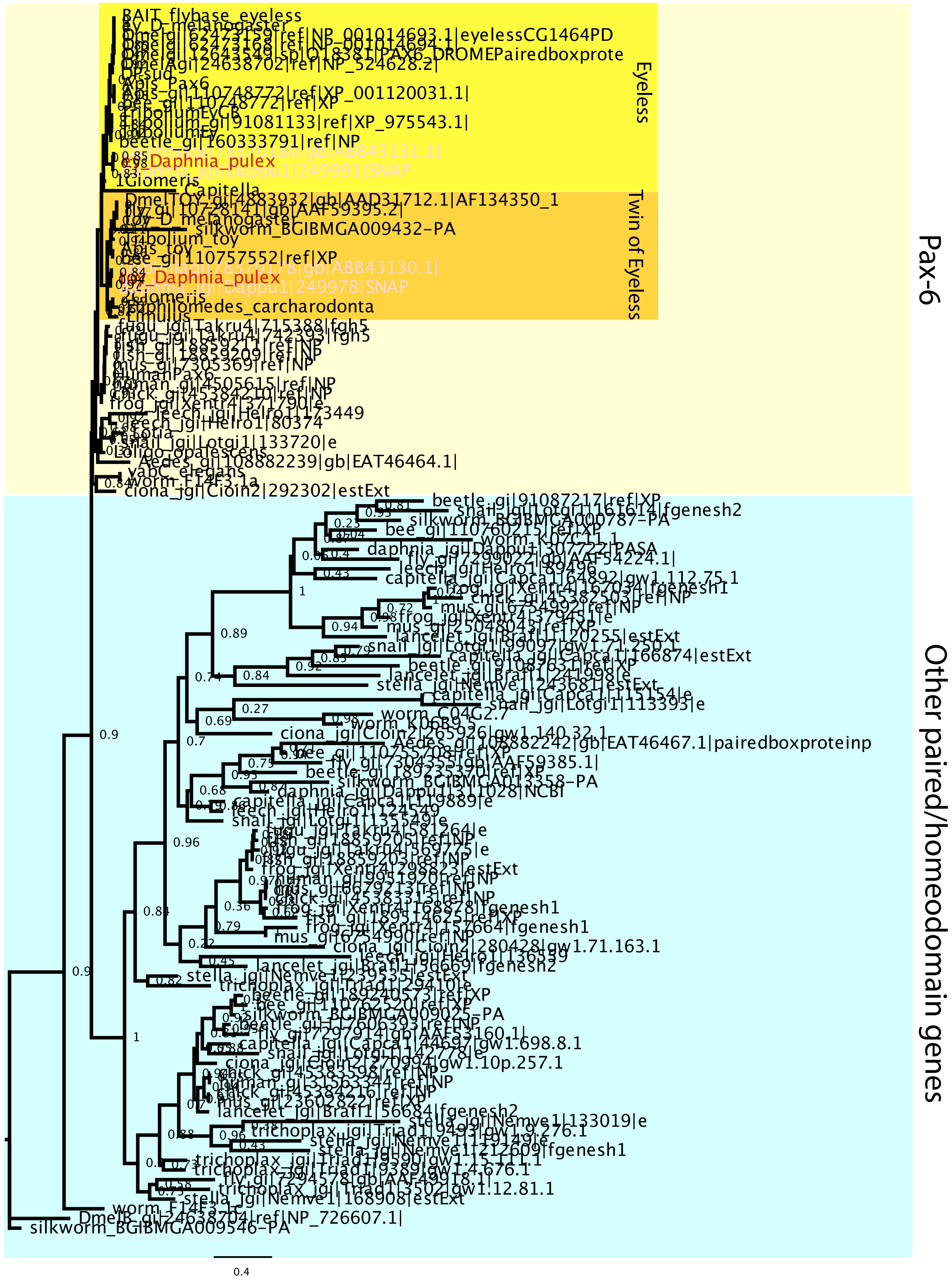
Eyeless
Twin of Eyeless
Pax-6
Other paired/homeodomain genes
0.4
BAIT_flybase_eyeless
ey_D_melanogaster
Dmel|gi|62473159|ref|NP_001014693.1|eyelessCG1464PD
Apis_Pax6
ey_Daphnia_pulex
Glomeris
Capitella
silkworm_BGIBMGA009432-PA
toy_Daphnia_pulex
Euphilomedes_carcharodonta
Limulus
Loligo_opalescens
Aedes_gi|108882239|gb|EAT46464.1|
vab3_elegans
ciona_jgi|Cioin2|292302|estExt
beetle_gi|91087217|ref|XP
capitella_jgi|Capca1|64892|gw1.112.75.1
Aedes_gi|108882242|gb|EAT46467.1|pairedboxproteinp
daphnia_jgi|Dappu1|311028|NCBI
lancelet_jgi|Brafl1|56684|fgeneshl2
stella_jgi|Nemve1|168908|estExt
Dmel|B_gi|24638704|ref|NP_726607.1|
silkworm_BGIBMGA009546-PA

S9 B-2

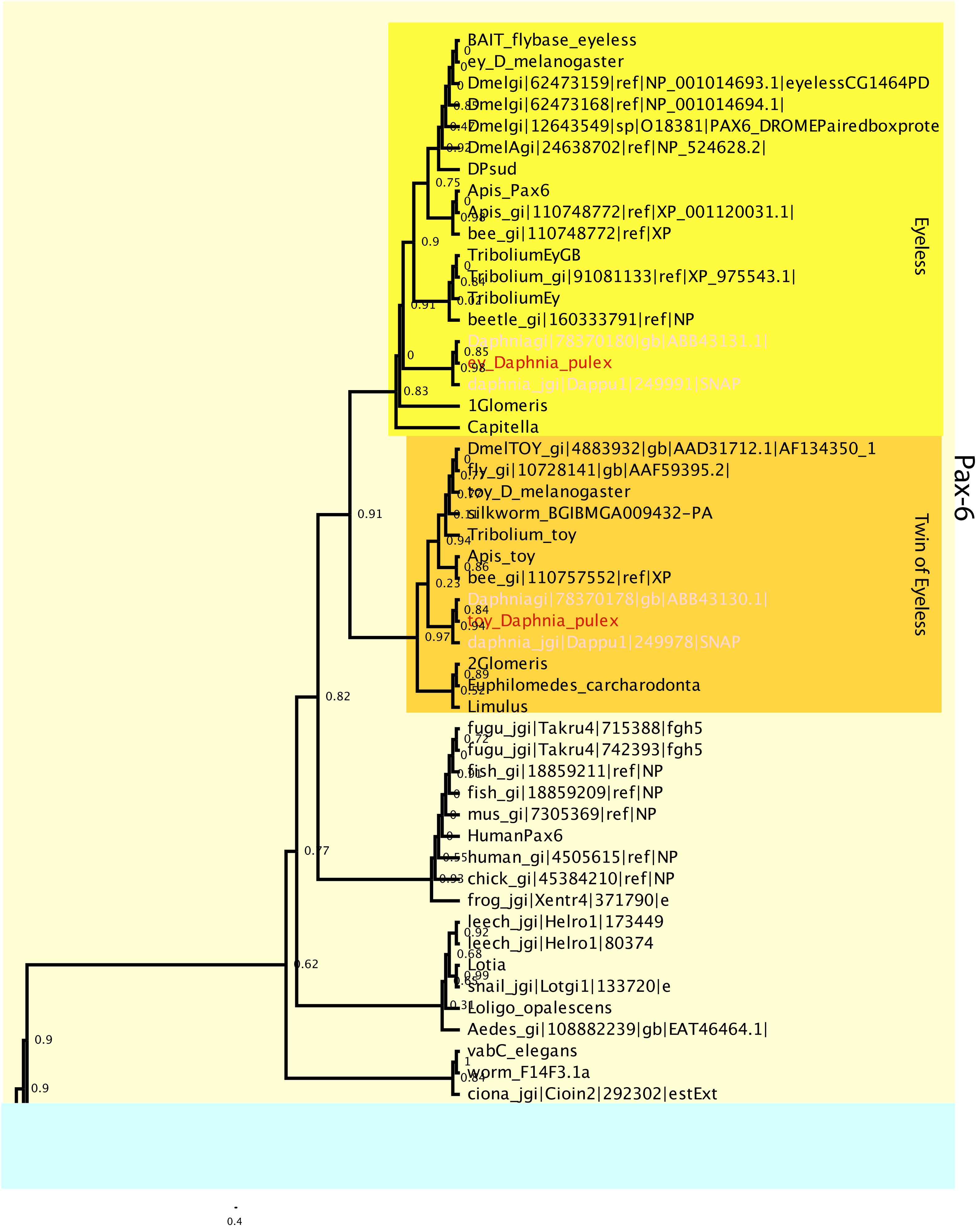
BAIT_flybase_eyeless
ey_D_melanogaster
Dmelgi|62473159|ref|NP_001014693.1|eyelessCG1464PD
Dmelgi|62473168|ref|NP_001014694.1|
Dmelgi|12643549|sp|O18381|PAX6_DROMEPairedboxprote
DmelAgi|24638702|ref|NP_524628.2|
DPsud
Apis_Pax6
Apis_gi|110748772|ref|XP_001120031.1|
bee_gi|110748772|ref|XP
TriboliumEyGB
Tribolium_gi|91081133|ref|XP_975543.1|
TriboliumEy
beetle_gi|160333791|ref|NP
Daphniagi|78370180|gb|ABB43131.1|
ey_Daphnia_pulex
daphnia_jgi|Dappu1|249991|SNAP
1Glomeris
Capitella
DmelTOY_gi|4883932|gb|AAD31712.1|AF134350_1
fly_gi|10728141|gb|AAF59395.2|
toy_D_melanogaster
silkworm_BGIBMGA009432-PA
Tribolium_toy
Apis_toy
bee_gi|110757552|ref|XP
Daphniagi|78370178|gb|ABB43130.1|
toy_Daphnia_pulex
daphnia_jgi|Dappu1|249978|SNAP
2Glomeris
Euphilomedes_carcharodonta
Limulus
fugu_jgi|Takru4|715388|fgh5
fugu_jgi|Takru4|742393|fgh5
fish_gi|18859211|ref|NP
fish_gi|18859209|ref|NP
mus_gi|7305369|ref|NP
HumanPax6
human_gi|4505615|ref|NP
chick_gi|45384210|ref|NP
frog_jgi|Xentr4|371790|e
leech_jgi|Helro1|173449
leech_jgi|Helro1|80374
Lotia
snail_jgi|Lotgi1|133720|e
Loligo_opalescens
Aedes_gi|108882239|gb|EAT46464.1|
vabC_elegans
worm_F14F3.1a
ciona_jgi|Cioin2|292302|estExt
Eyeless
Twin of Eyeless
Pax-6
0.4

S9 B-3

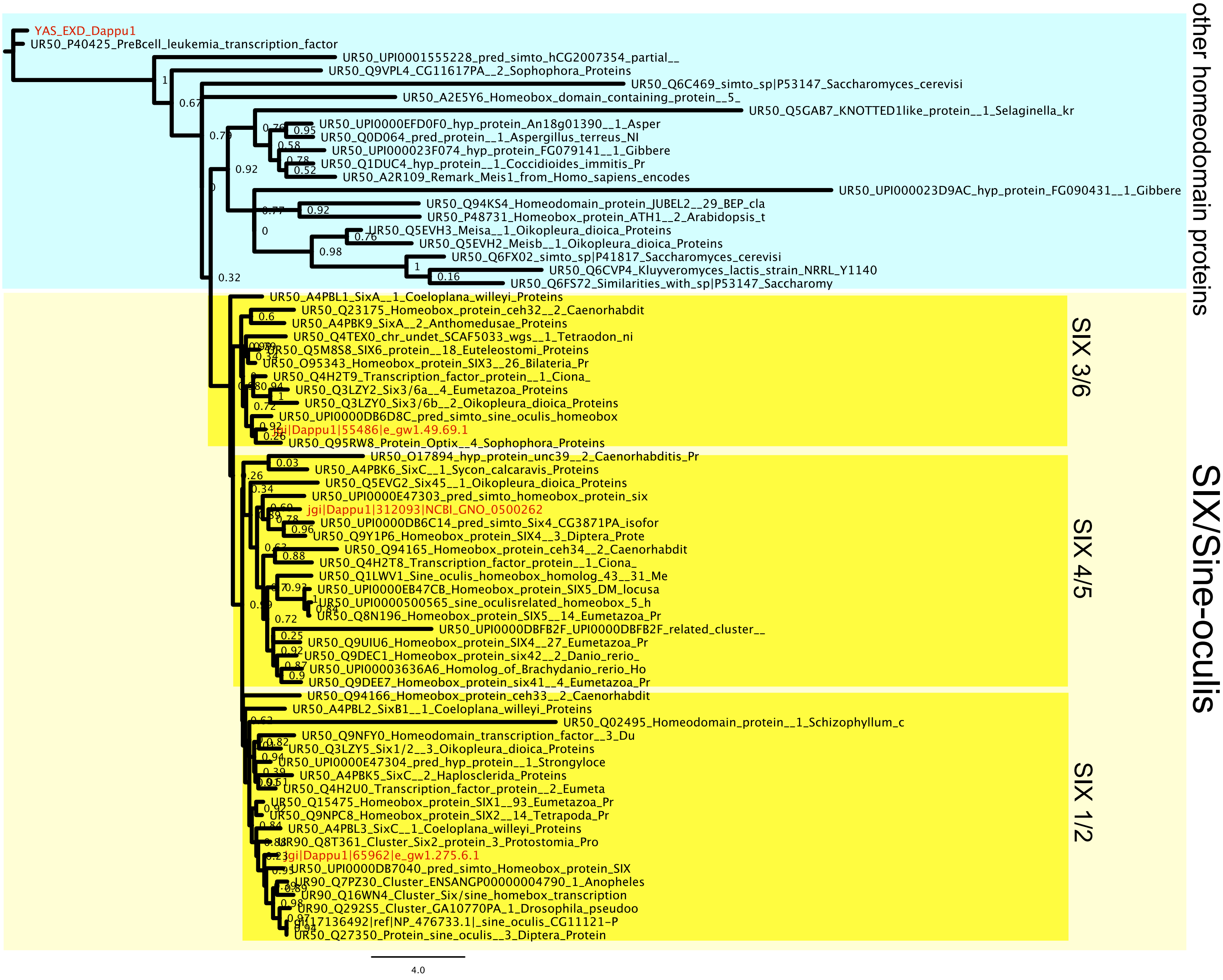
other homeodomain proteins
SIX/Sine-oculis
SIX 3/6
SIX 4/5
SIX 1/2
YAS_EXD_Dappu1
UR50_P40425_PreBcell_leukemia_transcription_factor
UR50_UPI0001555228_pred_simto_hCG2007354_partial__
UR50_Q9VPL4_CG11617PA__2_Sophophora_Proteins
UR50_Q6C469_simto_sp|P53147_Saccharomyces_cerevisi
UR50_A2E5Y6_Homeobox_domain_containing_protein__5_
UR50_Q5GAB7_KNOTTED1like_protein__1_Selaginella_kr
UR50_UPI0000EFD0F0_hyp_protein_An18g01390__1_Asper
UR50_Q0D064_pred_protein__1_Aspergillus_terreus_NI
UR50_UPI000023F074_hyp_protein_FG079141__1_Gibbere
UR50_Q1DUC4_hyp_protein__1_Coccidioides_immitis_Pr
UR50_A2R109_Remark_Meis1_from_Homo_sapiens_encodes
UR50_UPI000023D9AC_hyp_protein_FG090431__1_Gibbere
UR50_Q94KS4_Homeodomain_protein_JUBEL2__29_BEP_cla
UR50_P48731_Homeobox_protein_ATH1__2_Arabidopsis_t
UR50_Q5EVH3_Meisa__1_Oikopleura_dioica_Proteins
UR50_Q5EVH2_Meisb__1_Oikopleura_dioica_Proteins
UR50_Q6FX02_simto_sp|P41817_Saccharomyces_cerevisi
UR50_Q6CVP4_Kluyveromyces_lactis_strain_NRRL_Y1140
UR50_Q6FS72_Similarities_with_sp|P53147_Saccharomy
UR50_A4PBL1_SixA__1_Coeloplana_willeyi_Proteins
UR50_Q23175_Homeobox_protein_ceh32__2_Caenorhabdit
UR50_A4PBK9_SixA__2_Anthomedusae_Proteins
UR50_Q4TEX0_chr_undet_SCAF5033_wgs__1_Tetraodon_ni
UR50_Q5M8S8_SIX6_protein__18_Euteleostomi_Proteins
UR50_O95343_Homeobox_protein_SIX3__26_Bilateria_Pr
UR50_Q4H2T9_Transcription_factor_protein__1_Ciona_
UR50_Q3LZY2_Six3/6a__4_Eumetazoa_Proteins
UR50_Q3LZY0_Six3/6b__2_Oikopleura_dioica_Proteins
UR50_UPI0000DB6D8C_pred_simto_sine_oculis_homeobox
jgi|Dappu1|55486|e_gw1.49.69.1
UR50_Q95RW8_Protein_Optix__4_Sophophora_Proteins
UR50_O17894_hyp_protein_unc39__2_Caenorhabditis_Pr
UR50_A4PBK6_SixC__1_Sycon_calcaravis_Proteins
UR50_Q5EVG2_Six45__1_Oikopleura_dioica_Proteins
UR50_UPI0000E47303_pred_simto_homeobox_protein_six
jgi|Dappu1|312093|NCBI_GNO_0500262
UR50_UPI0000DB6C14_pred_simto_Six4_CG3871PA_isofor
UR50_Q9Y1P6_Homeobox_protein_SIX4__3_Diptera_Prote
UR50_Q94165_Homeobox_protein_ceh34__2_Caenorhabdit
UR50_Q4H2T8_Transcription_factor_protein__1_Ciona_
UR50_Q1LWV1_Sine_oculis_homeobox_homolog_43__31_Me
UR50_UPI0000EB47CB_Homeobox_protein_SIX5_DM_locusa
UR50_UPI0000500565_sine_oculisrelated_homeobox_5_h
UR50_Q8N196_Homeobox_protein_SIX5__14_Eumetazoa_Pr
UR50_UPI0000DBFB2F_UPI0000DBFB2F_related_cluster__
UR50_Q9UIU6_Homeobox_protein_SIX4__27_Eumetazoa_Pr
UR50_Q9DEC1_Homeobox_protein_six42__2_Danio_rerio_
UR50_UPI00003636A6_Homolog_of_Brachydanio_rerio_Ho
UR50_Q9DEE7_Homeobox_protein_six41__4_Eumetazoa_Pr
UR50_Q94166_Homeobox_protein_ceh33__2_Caenorhabdit
UR50_A4PBL2_SixB1__1_Coeloplana_willeyi_Proteins
UR50_Q02495_Homeodomain_protein__1_Schizophyllum_c
UR50_Q9NFY0_Homeodomain_transcription_factor__3_Du
UR50_Q3LZY5_Six1/2__3_Oikopleura_dioica_Proteins
UR50_UPI0000E47304_pred_hyp_protein__1_Strongyloce
UR50_A4PBK5_SixC__2_Haplosclerida_Proteins
UR50_Q4H2U0_Transcription_factor_protein__2_Eumeta
UR50_Q15475_Homeobox_protein_SIX1__93_Eumetazoa_Pr
UR50_Q9NPC8_Homeobox_protein_SIX2__14_Tetrapoda_Pr
UR50_A4PBL3_SixC__1_Coeloplana_willeyi_Proteins
UR90_Q8T361_Cluster_Six2_protein_3_Protostomia_Pro
jgi|Dappu1|65962|e_gw1.275.6.1
UR50_UPI0000DB7040_pred_simto_Homeobox_protein_SIX
UR90_Q7PZ30_Cluster_ENSANGP00000004790_1_Anopheles
UR90_Q16WN4_Cluster_Six/sine_homebox_transcription
UR90_Q292S5_Cluster_GA10770PA_1_Drosophila_pseudoo
gi|17136492|ref|NP_476733.1|_sine_oculis_CG11121-P
UR50_Q27350_Protein_sine_oculis__3_Diptera_Protein
4.0

S10 A

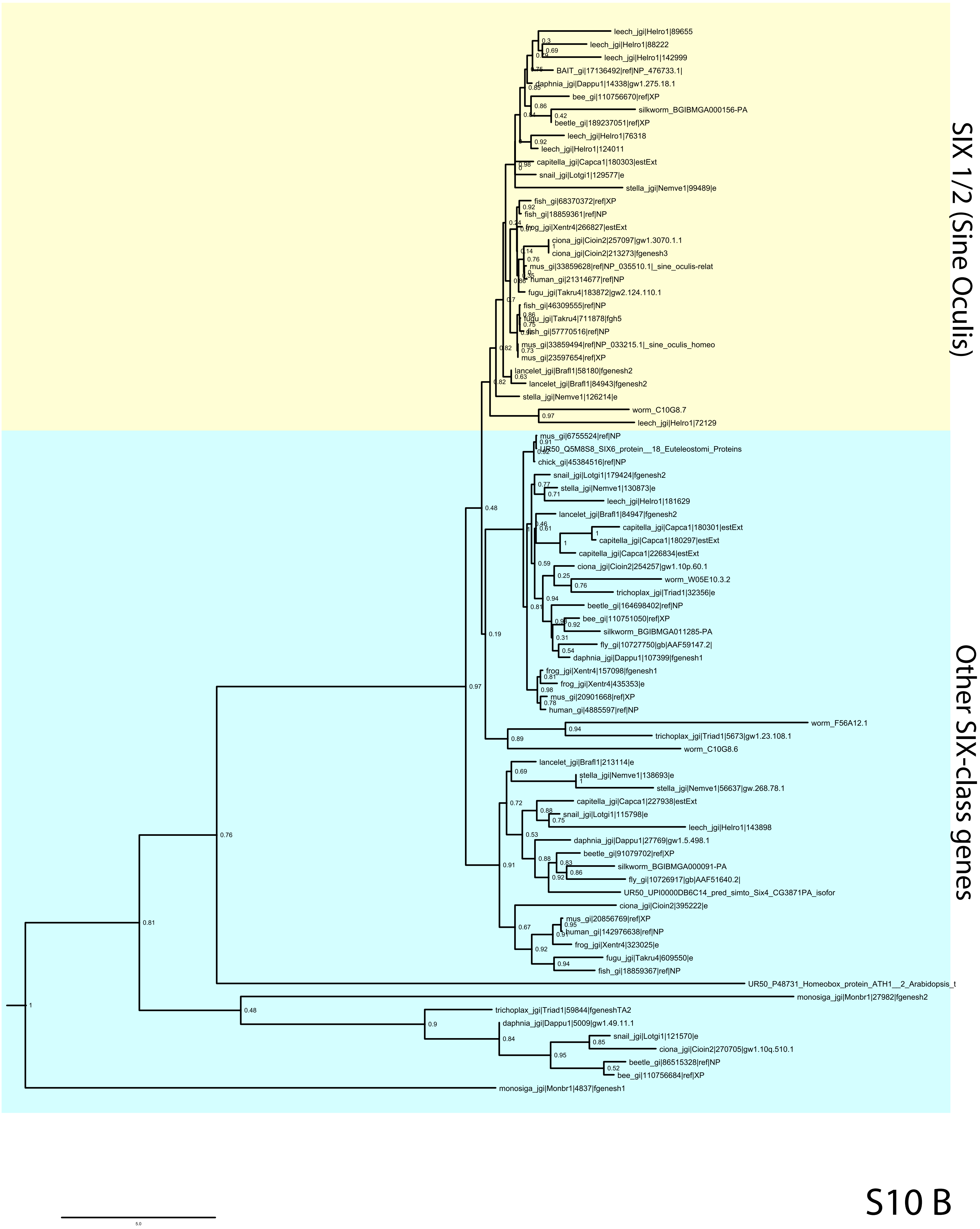
SIX 1/2 (Sine Oculis)
Other SIX-class genes
leech_jgi|Helro1|89655
leech_jgi|Helro1|88222
leech_jgi|Helro1|142999
BAIT_gi|17136492|ref|NP_476733.1|
daphnia_jgi|Dappu1|14338|gw1.275.18.1
bee_gi|110756670|ref|XP
silkworm_BGIBMGA000156-PA
beetle_gi|189237051|ref|XP
leech_jgi|Helro1|76318
leech_jgi|Helro1|124011
capitella_jgi|Capca1|180303|estExt
snail_jgi|Lotgi1|129577|e
stella_jgi|Nemve1|99489|e
fish_gi|68370372|ref|XP
fish_gi|18859361|ref|NP
frog_jgi|Xentr4|266827|estExt
ciona_jgi|Cioin2|257097|gw1.3070.1.1
ciona_jgi|Cioin2|213273|fgenesh3
mus_gi|33859628|ref|NP_035510.1|_sine_oculis-relat
human_gi|21314677|ref|NP
fugu_jgi|Takru4|183872|gw2.124.110.1
fish_gi|46309555|ref|NP
fugu_jgi|Takru4|711878|fgh5
fish_gi|57770516|ref|NP
mus_gi|33859494|ref|NP_033215.1|_sine_oculis_homeo
mus_gi|23597654|ref|XP
lancelet_jgi|Brafl1|58180|fgenesh2
lancelet_jgi|Brafl1|84943|fgenesh2
stella_jgi|Nemve1|126214|e
worm_C10G8.7
leech_jgi|Helro1|72129
mus_gi|6755524|ref|NP
UR50_Q5M8S8_SIX6_protein__18_Euteleostomi_Proteins
chick_gi|45384516|ref|NP
snail_jgi|Lotgi1|179424|fgenesh2
stella_jgi|Nemve1|130873|e
leech_jgi|Helro1|181629
lancelet_jgi|Brafl1|84947|fgenesh2
capitella_jgi|Capca1|180301|estExt
capitella_jgi|Capca1|180297|estExt
capitella_jgi|Capca1|226834|estExt
ciona_jgi|Cioin2|254257|gw1.10p.60.1
worm_W05E10.3.2
trichoplax_jgi|Triad1|32356|e
beetle_gi|164698402|ref|NP
bee_gi|110751050|ref|XP
silkworm_BGIBMGA011285-PA
fly_gi|10727750|gb|AAF59147.2|
daphnia_jgi|Dappu1|107399|fgenesh1
frog_jgi|Xentr4|157098|fgenesh1
frog_jgi|Xentr4|435353|e
mus_gi|20901668|ref|XP
human_gi|4885597|ref|NP
worm_F56A12.1
trichoplax_jgi|Triad1|5673|gw1.23.108.1
worm_C10G8.6
lancelet_jgi|Brafl1|213114|e
stella_jgi|Nemve1|138693|e
stella_jgi|Nemve1|56637|gw.268.78.1
capitella_jgi|Capca1|227938|estExt
snail_jgi|Lotgi1|115798|e
leech_jgi|Helro1|143898
daphnia_jgi|Dappu1|27769|gw1.5.498.1
beetle_gi|91079702|ref|XP
silkworm_BGIBMGA000091-PA
fly_gi|10726917|gb|AAF51640.2|
UR50_UPI0000DB6C14_pred_simto_Six4_CG3871PA_isofor
ciona_jgi|Cioin2|395222|e
mus_gi|20856769|ref|XP
human_gi|142976638|ref|NP
frog_jgi|Xentr4|323025|e
fugu_jgi|Takru4|609550|e
fish_gi|18859367|ref|NP
UR50_P48731_Homeobox_protein_ATH1__2_Arabidopsis_t
monosiga_jgi|Monbr1|27982|fgenesh2
trichoplax_jgi|Triad1|59844|fgeneshTA2
daphnia_jgi|Dappu1|5009|gw1.49.11.1
snail_jgi|Lotgi1|121570|e
ciona_jgi|Cioin2|270705|gw1.10q.510.1
beetle_gi|86515328|ref|NP
bee_gi|110756684|ref|XP
monosiga_jgi|Monbr1|4837|fgenesh1
5.0

S10 B

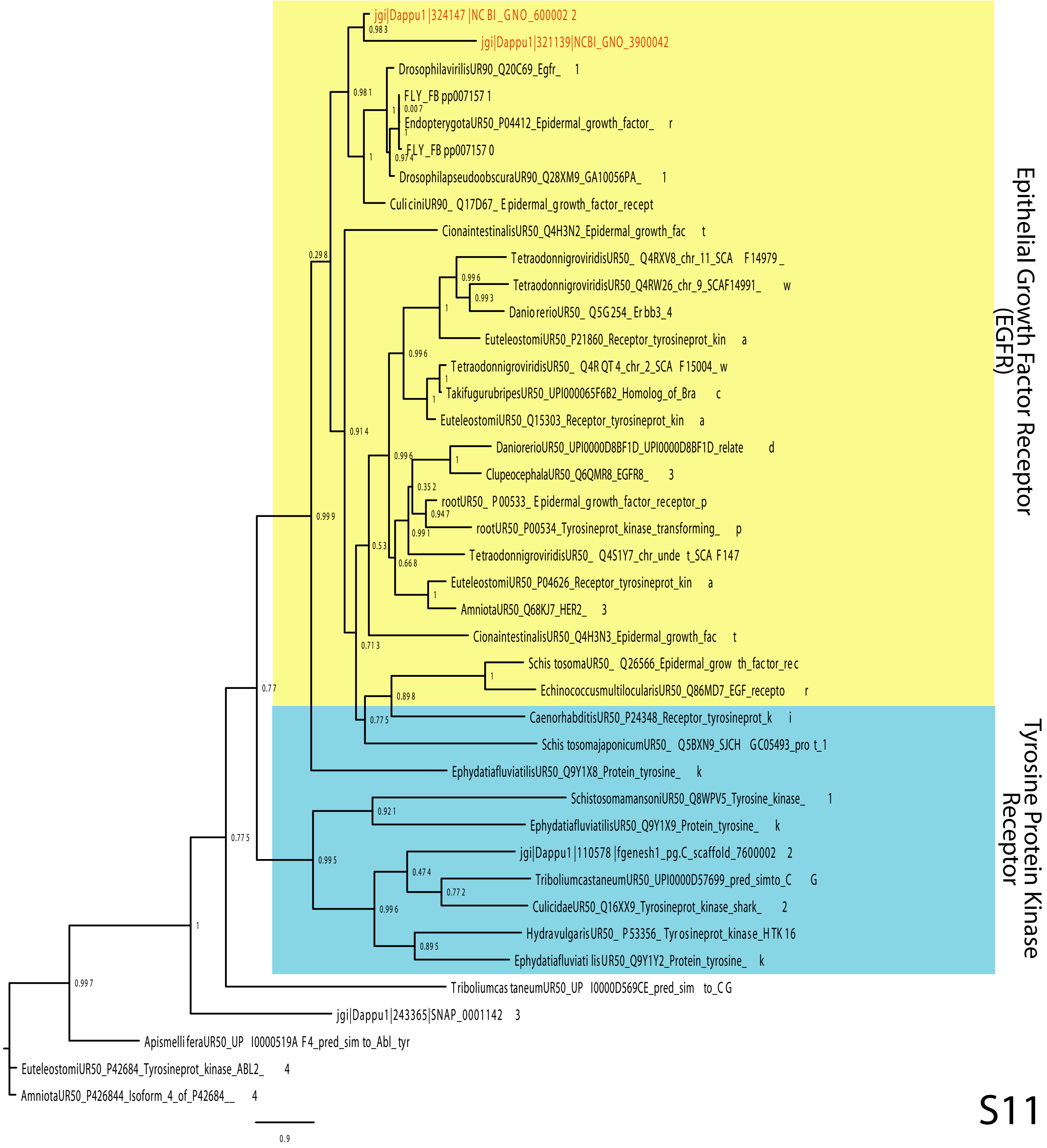

jgi|Dappu1|324147|NCBI_GNO_6000022
jgi|Dappu1|321139|NCBI_GNO_3900042
DrosophilavirilisUR90_Q20C69_Egfr_ 1
FLY_FBpp0071571
EndopterygotaUR50_P04412_Epidermal_growth_factor_ r
FLY_FBpp0071570
DrosophilapseudoobscuraUR90_Q28XM9_GA10056PA_ 1
CuliciniUR90_Q17D67_Epidermal_growth_factor_recept
CionaintestinalisUR50_Q4H3N2_Epidermal_growth_fac t
TetraodonnigroviridisUR50_Q4RXV8_chr_11_SCA F14979_
TetraodonnigroviridisUR50_Q4RW26_chr_9_SCAF14991_ w
DaniorerioUR50_Q5G254_Erbb3_4
EuteleostomiUR50_P21860_Receptor_tyrosineprot_kin a
TetraodonnigroviridisUR50_Q4RQT4_chr_2_SCA F15004_w
TakifugurubripesUR50_UPI000065F6B2_Homolog_of_Bra c
EuteleostomiUR50_Q15303_Receptor_tyrosineprot_kin a
DaniorerioUR50_UPI0000D8BF1D_UPI0000D8BF1D_relate d
ClupeocephalaUR50_Q6QMR8_EGFR8_ 3
rootUR50_P00533_Epidermal_growth_factor_receptor_p
rootUR50_P00534_Tyrosineprot_kinase_transforming_ p
TetraodonnigroviridisUR50_Q4S1Y7_chr_unde t_SCA F147
EuteleostomiUR50_P04626_Receptor_tyrosineprot_kin a
AmniotaUR50_Q68KJ7_HER2_ 3
CionaintestinalisUR50_Q4H3N3_Epidermal_growth_fac t
SchistosomaUR50_Q26566_Epidermal_grow th_factor_rec
EchinococcusmultilocularisUR50_Q86MD7_EGF_recepto r
CaenorhabditisUR50_P24348_Receptor_tyrosineprot_k i
SchistosomajaponicumUR50_Q5BXN9_SJCH GC05493_pro t_1
EphydatiafluviatilisUR50_Q9Y1X8_Protein_tyrosine_ k
SchistosomamansoniUR50_Q8WPV5_Tyrosine_kinase_ 1
EphydatiafluviatilisUR50_Q9Y1X9_Protein_tyrosine_ k
jgi|Dappu1|110578|fgenesh1_pg.C_scaffold_7600002 2
TriboliumcastaneumUR50_UPI0000D57699_pred_simto_C G
CulicidaeUR50_Q16XX9_Tyrosineprot_kinase_shark_ 2
HydravulgarisUR50_P53356_Tyrosineprot_kinase_HTK16
EphydatiafluviatilisUR50_Q9Y1Y2_Protein_tyrosine_ k
TriboliumcastaneumUR50_UP I0000D569CE_pred_sim to_CG
jgi|Dappu1|243365|SNAP_0001142 3
ApismelliferaUR50_UP I0000519A F4_pred_sim to_Abl_tyr
EuteleostomiUR50_P42684_Tyrosineprot_kinase_ABL2_ 4
AmniotaUR50_P426844_Isoform_4_of_P42684__ 4
0.983
0.981
1
0.007
1
0.974
1
0.298
0.996
0.993
1
0.996
1
1
0.914
0.996
1
0.352
0.947
0.991
0.53
0.668
1
0.713
1
0.898
0.775
0.999
0.77
0.921
0.995
0.474
0.772
0.996
0.895
0.775
1
0.997
0.9
Epithelial Growth Factor Receptor (EGFR)
Tyrosine Protein Kinase Receptor

S11 A

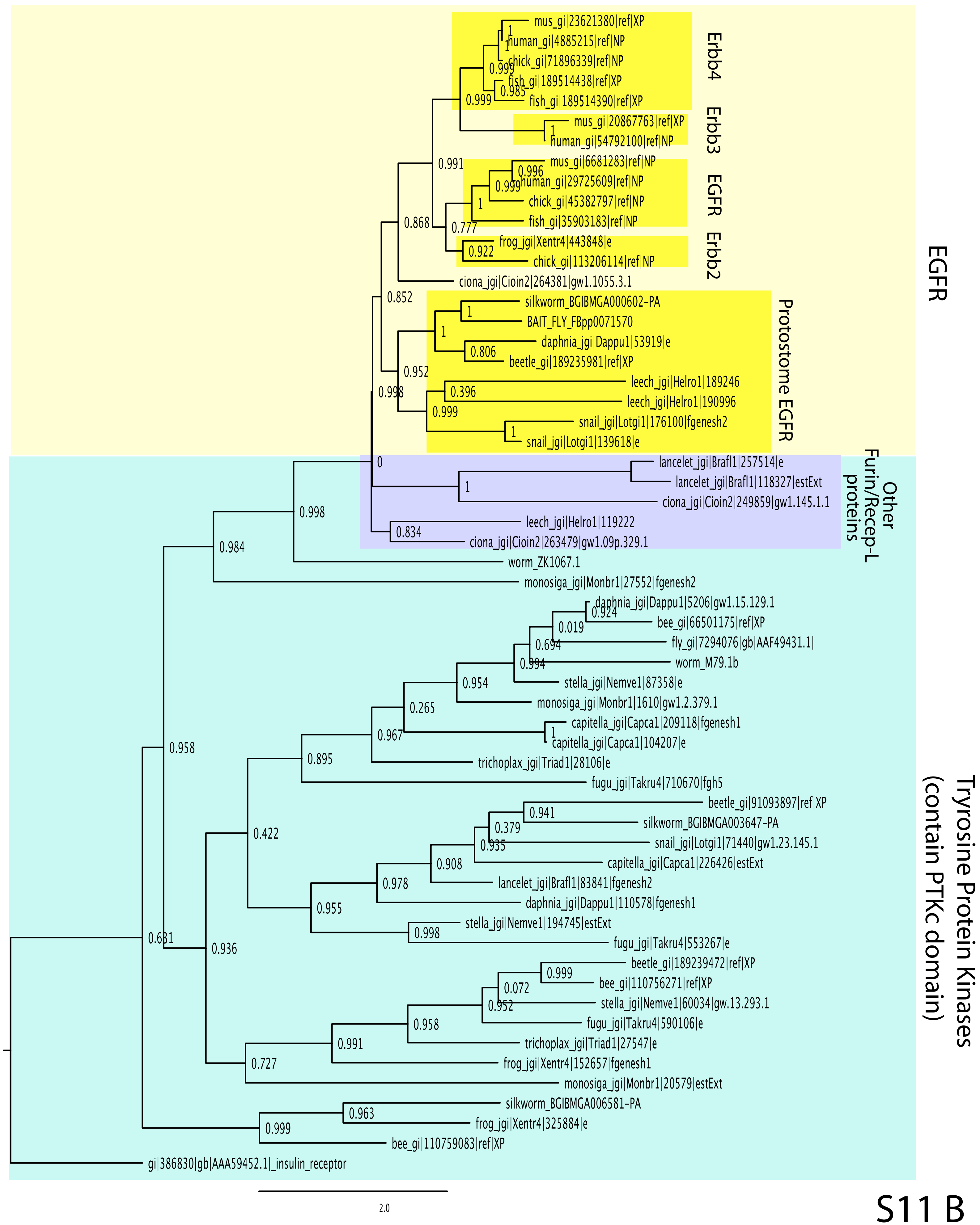
EGFR
Erbb4
Erbb3
EGFR
Erbb2
Protostome EGFR
Other Furin/Recep-L proteins
Tyrosine Protein Kinases (contain PTKc domain)
mus_gi|23621380|ref|XP
human_gi|4885215|ref|NP
chick_gi|71896339|ref|NP
fish_gi|189514438|ref|XP
fish_gi|189514390|ref|XP
mus_gi|20867763|ref|XP
human_gi|54792100|ref|NP
mus_gi|6681283|ref|NP
human_gi|29725609|ref|NP
chick_gi|45382797|ref|NP
fish_gi|35903183|ref|NP
frog_jgi|Xentr4|443848|e
chick_gi|113206114|ref|NP
ciona_jgi|Cioin2|264381|gw1.1055.3.1
silkworm_BGIBMGA000602-PA
BAIT_FLY_FBpp0071570
daphnia_jgi|Dappu1|53919|e
beetle_gi|189235981|ref|XP
leech_jgi|Helro1|189246
leech_jgi|Helro1|190996
snail_jgi|Lotgi1|176100|fgenesh2
snail_jgi|Lotgi1|139618|e
lancelet_jgi|Brafl1|257514|e
lancelet_jgi|Brafl1|118327|estExt
ciona_jgi|Cioin2|249859|gw1.145.1.1
leech_jgi|Helro1|119222
ciona_jgi|Cioin2|263479|gw1.09p.329.1
worm_ZK1067.1
monosiga_jgi|Monbr1|27552|fgenesh2
daphnia_jgi|Dappu1|5206|gw1.15.129.1
bee_gi|66501175|ref|XP
fly_gi|7294076|gb|AAF49431.1|
worm_M79.1b
stella_jgi|Nemve1|87358|e
monosiga_jgi|Monbr1|1610|gw1.2.379.1
capitella_jgi|Capca1|209118|fgenesh1
capitella_jgi|Capca1|104207|e
trichoplax_jgi|Triad1|28106|e
fugu_jgi|Takru4|710670|fgh5
beetle_gi|91093897|ref|XP
silkworm_BGIBMGA003647-PA
snail_jgi|Lotgi1|71440|gw1.23.145.1
capitella_jgi|Capca1|226426|estExt
lancelet_jgi|Brafl1|83841|fgenesh2
daphnia_jgi|Dappu1|110578|fgenesh1
stella_jgi|Nemve1|194745|estExt
fugu_jgi|Takru4|553267|e
beetle_gi|189239472|ref|XP
bee_gi|110756271|ref|XP
stella_jgi|Nemve1|60034|gw.13.293.1
fugu_jgi|Takru4|590106|e
trichoplax_jgi|Triad1|27547|e
frog_jgi|Xentr4|152657|fgenesh1
monosiga_jgi|Monbr1|20579|estExt
silkworm_BGIBMGA006581-PA
frog_jgi|Xentr4|325884|e
bee_gi|110759083|ref|XP
gi|386830|gb|AAA59452.1|_insulin_receptor
2.0

S11 B

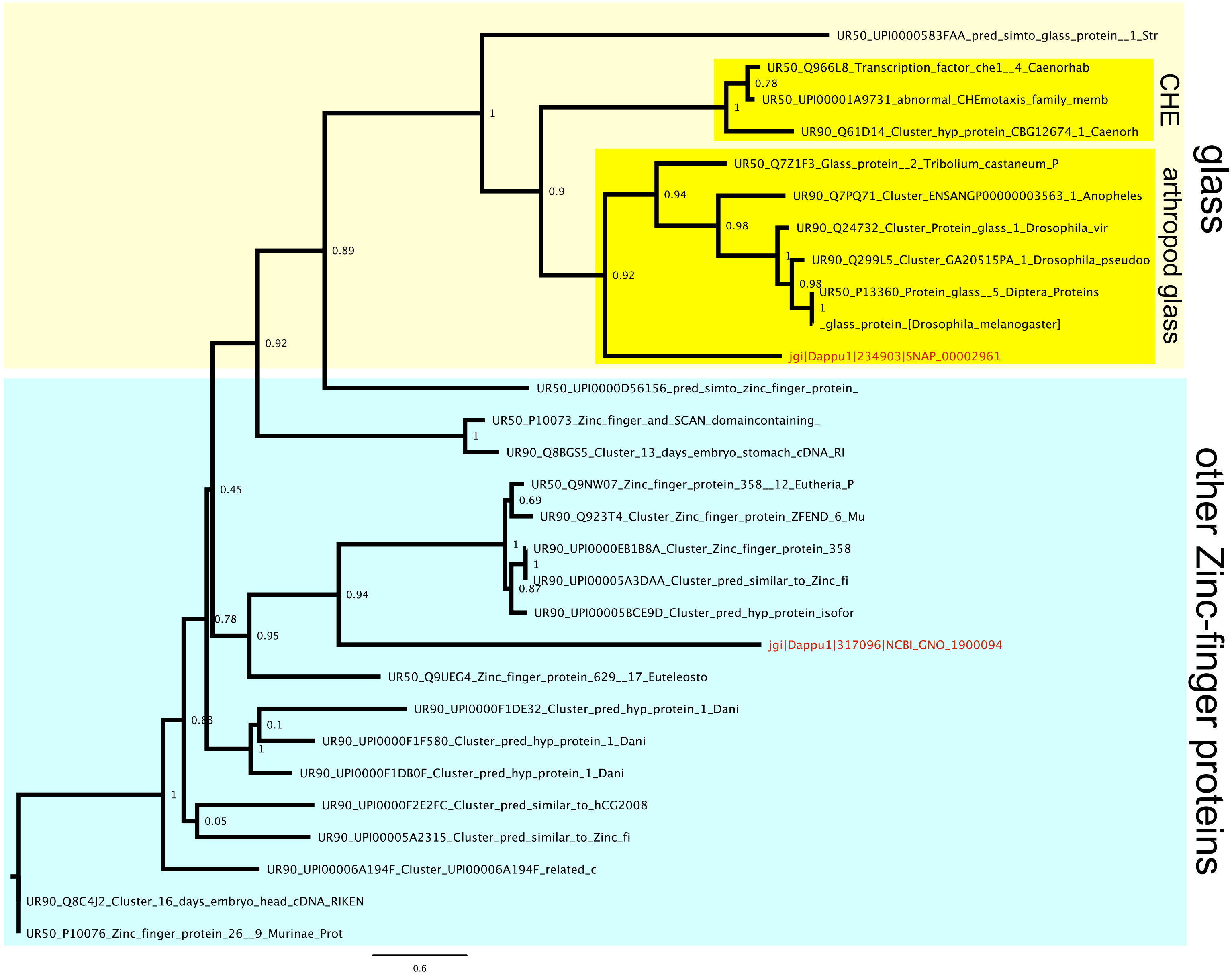
UR50_UPI0000583FAA_pred_simto_glass_protein__1_Str
UR50_Q966L8_Transcription_factor_che1__4_Caenorhab
UR50_UPI00001A9731_abnormal_CHEmotaxis_family_memb
UR90_Q61D14_Cluster_hyp_protein_CBG12674_1_Caenorh
UR50_Q7Z1F3_Glass_protein__2_Tribolium_castaneum_P
UR90_Q7PQ71_Cluster_ENSANGP00000003563_1_Anopheles
UR90_Q24732_Cluster_Protein_glass_1_Drosophila_vir
UR90_Q299L5_Cluster_GA20515PA_1_Drosophila_pseudoo
UR50_P13360_Protein_glass__5_Diptera_Proteins
_glass_protein_[Drosophila_melanogaster]
jgi|Dappu1|234903|SNAP_00002961
UR50_UPI0000D56156_pred_simto_zinc_finger_protein_
UR50_P10073_Zinc_finger_and_SCAN_domaincontaining_
UR90_Q8BGS5_Cluster_13_days_embryo_stomach_cDNA_RI
UR50_Q9NW07_Zinc_finger_protein_358__12_Eutheria_P
UR90_Q923T4_Cluster_Zinc_finger_protein_ZFEND_6_Mu
UR90_UPI0000EB1B8A_Cluster_Zinc_finger_protein_358
UR90_UPI00005A3DAA_Cluster_pred_similar_to_Zinc_fi
UR90_UPI00005BCE9D_Cluster_pred_hyp_protein_isofor
jgi|Dappu1|317096|NCBI_GNO_1900094
UR50_Q9UEG4_Zinc_finger_protein_629__17_Euteleosto
UR90_UPI0000F1DE32_Cluster_pred_hyp_protein_1_Dani
UR90_UPI0000F1F580_Cluster_pred_hyp_protein_1_Dani
UR90_UPI0000F1DB0F_Cluster_pred_hyp_protein_1_Dani
UR90_UPI0000F2E2FC_Cluster_pred_similar_to_hCG2008
UR90_UPI00005A2315_Cluster_pred_similar_to_Zinc_fi
UR90_UPI00006A194F_Cluster_UPI00006A194F_related_c
UR90_Q8C4J2_Cluster_16_days_embryo_head_cDNA_RIKEN
UR50_P10076_Zinc_finger_protein_26__9_Murinae_Prot
1
0.78
0.9
0.94
0.98
0.92
0.89
0.92
0.45
0.69
0.87
0.94
0.78
0.95
0.83
0.1
0.05
0.6
CHE
arthropod glass
glass
other Zinc-finger proteins

S12 A

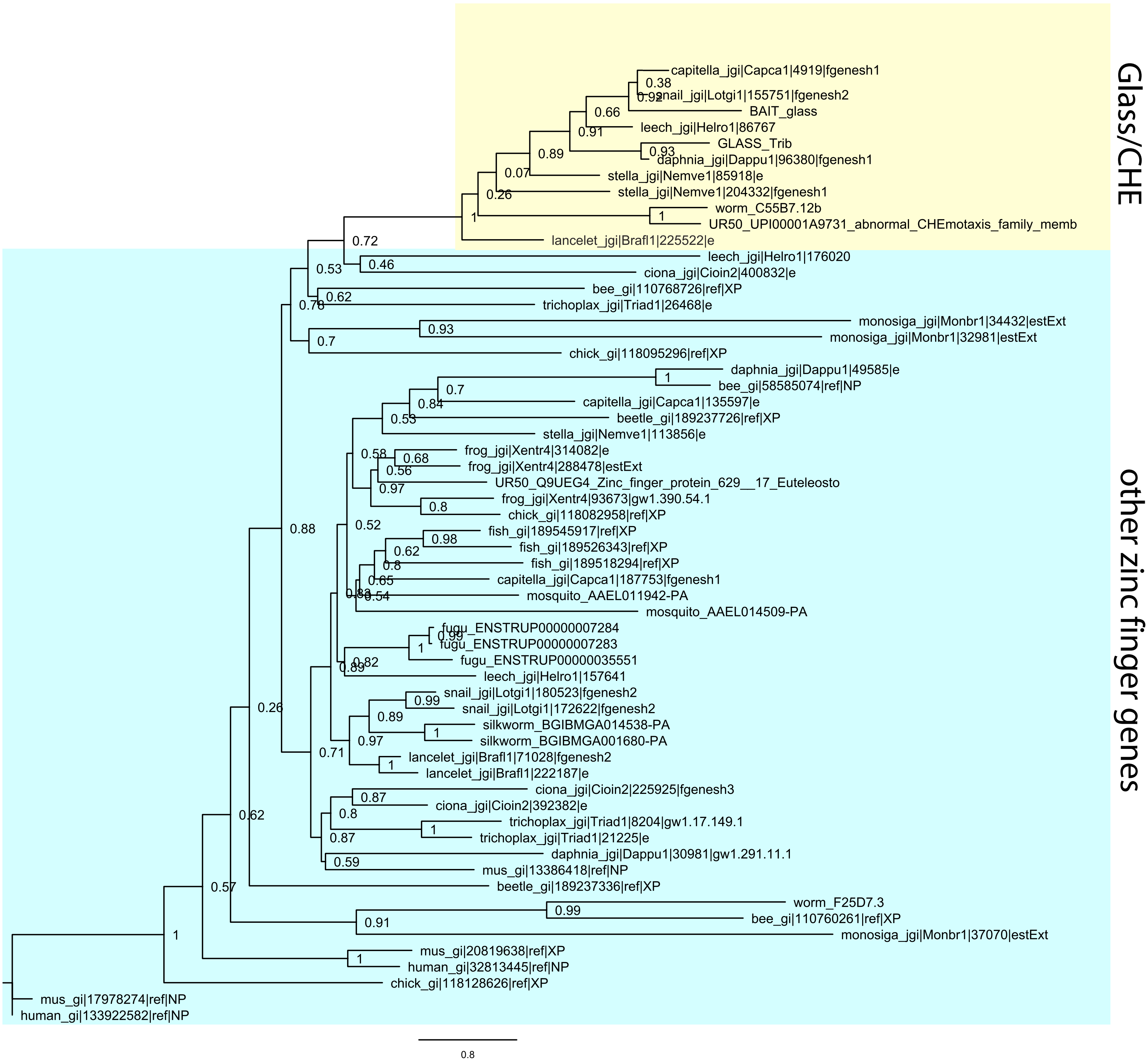
capitella_jgi|Capca1|4919|fgenesh1
0.38
0.92
snail_jgi|Lotgi1|155751|fgenesh2
BAIT_glass
0.66
leech_jgi|Helro1|86767
0.91
GLASS_Trib
0.93
daphnia_jgi|Dappu1|96380|fgenesh1
0.89
0.07
stella_jgi|Nemve1|85918|e
stella_jgi|Nemve1|204332|fgenesh1
0.26
worm_C55B7.12b
1
1
UR50_UPI00001A9731_abnormal_CHEmotaxis_family_memb
0.72
lancelet_jgi|Brafl1|225522|e
leech_jgi|Helro1|176020
0.53
0.46
ciona_jgi|Cioin2|400832|e
bee_gi|110768726|ref|XP
0.62
0.78
trichoplax_jgi|Triad1|26468|e
monosiga_jgi|Monbr1|34432|estExt
0.93
monosiga_jgi|Monbr1|32981|estExt
0.7
chick_gi|118095296|ref|XP
daphnia_jgi|Dappu1|49585|e
1
bee_gi|58585074|ref|NP
0.7
0.84
capitella_jgi|Capca1|135597|e
beetle_gi|189237726|ref|XP
0.53
stella_jgi|Nemve1|113856|e
0.58
frog_jgi|Xentr4|314082|e
0.68
frog_jgi|Xentr4|288478|estExt
0.56
UR50_Q9UEG4_Zinc_finger_protein_629__17_Euteleosto
0.97
frog_jgi|Xentr4|93673|gw1.390.54.1
0.8
chick_gi|118082958|ref|XP
0.52
0.88
fish_gi|189545917|ref|XP
0.98
fish_gi|189526343|ref|XP
0.62
0.8
fish_gi|189518294|ref|XP
0.65
capitella_jgi|Capca1|187753|fgenesh1
0.83
0.54
mosquito_AAEL011942-PA
mosquito_AAEL014509-PA
fugu_ENSTRUP00000007284
0.99
fugu_ENSTRUP00000007283
1
fugu_ENSTRUP00000035551
0.82
0.89
leech_jgi|Helro1|157641
0.26
snail_jgi|Lotgi1|180523|fgenesh2
0.99
snail_jgi|Lotgi1|172622|fgenesh2
0.89
silkworm_BGIBMGA014538-PA
1
silkworm_BGIBMGA001680-PA
0.97
0.71
lancelet_jgi|Brafl1|71028|fgenesh2
1
lancelet_jgi|Brafl1|222187|e
ciona_jgi|Cioin2|225925|fgenesh3
0.87
ciona_jgi|Cioin2|392382|e
0.62
0.8
trichoplax_jgi|Triad1|8204|gw1.17.149.1
1
0.87
trichoplax_jgi|Triad1|21225|e
daphnia_jgi|Dappu1|30981|gw1.291.11.1
0.59
mus_gi|13386418|ref|NP
0.57
beetle_gi|189237336|ref|XP
worm_F25D7.3
0.99
bee_gi|110760261|ref|XP
0.91
monosiga_jgi|Monbr1|37070|estExt
1
mus_gi|20819638|ref|XP
1
human_gi|32813445|ref|NP
chick_gi|118128626|ref|XP
mus_gi|17978274|ref|NP
human_gi|133922582|ref|NP
0.8
Glass/CHE
other zinc finger genes

S12 B

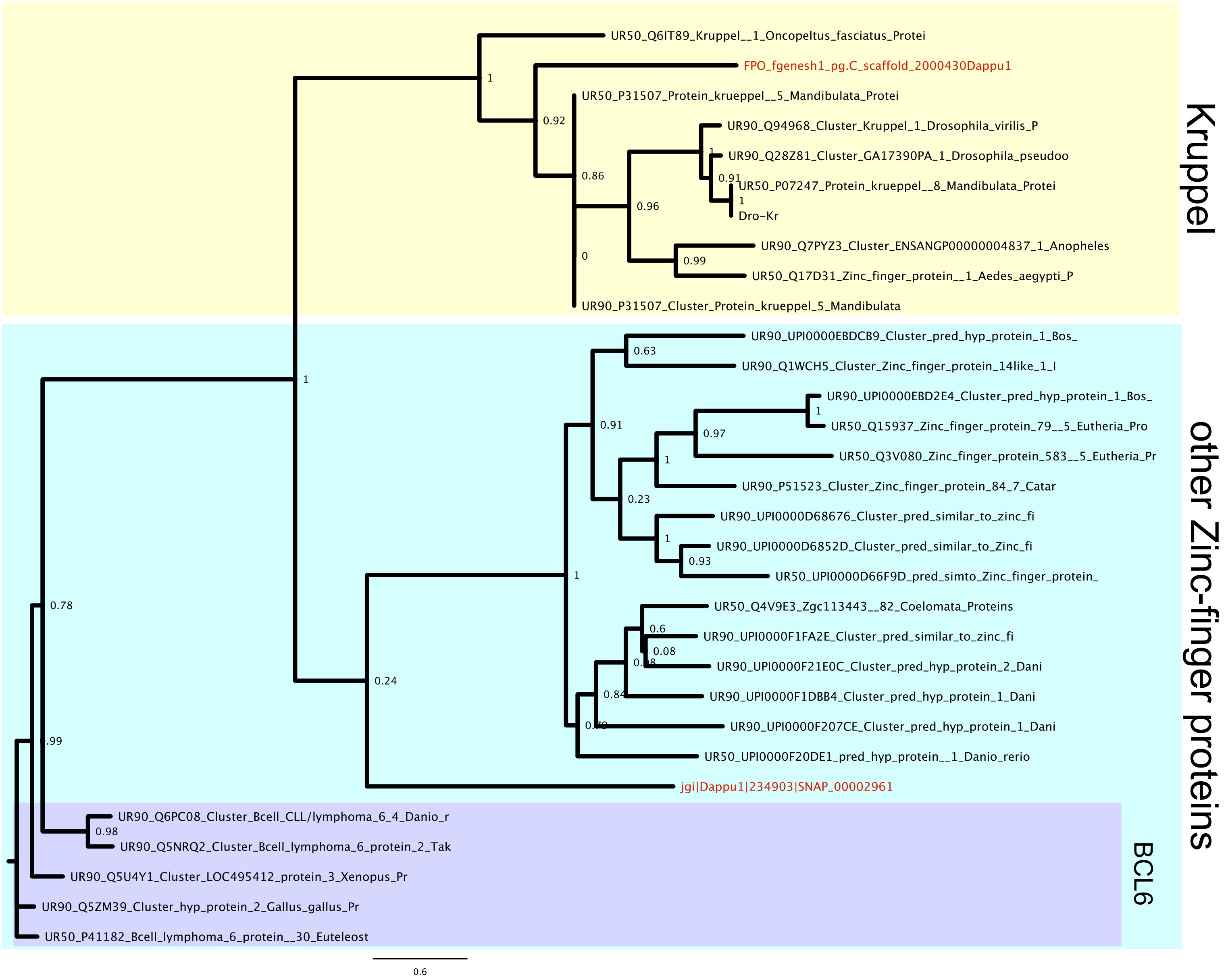

UR50_Q6IT89_Kruppel__1_Oncopeltus_fasciatus_Protei
FPO_fgenesh1_pg.C_scaffold_2000430Dappu1
UR50_P31507_Protein_krueppel__5_Mandibulata_Protei
UR90_Q94968_Cluster_Kruppel_1_Drosophila_virilis_P
UR90_Q28Z81_Cluster_GA17390PA_1_Drosophila_pseudoo
UR50_P07247_Protein_krueppel__8_Mandibulata_Protei
Dro-Kr
UR90_Q7PYZ3_Cluster_ENSANGP00000004837_1_Anopheles
UR50_Q17D31_Zinc_finger_protein__1_Aedes_aegypti_P
UR90_P31507_Cluster_Protein_krueppel_5_Mandibulata
UR90_UPI0000EBDCB9_Cluster_pred_hyp_protein_1_Bos_
UR90_Q1WCH5_Cluster_Zinc_finger_protein_14like_1_I
UR90_UPI0000EBD2E4_Cluster_pred_hyp_protein_1_Bos_
UR50_Q15937_Zinc_finger_protein_79__5_Eutheria_Pro
UR50_Q3V080_Zinc_finger_protein_583__5_Eutheria_Pr
UR90_P51523_Cluster_Zinc_finger_protein_84_7_Catar
UR90_UPI0000D68676_Cluster_pred_similar_to_zinc_fi
UR90_UPI0000D6852D_Cluster_pred_similar_to_Zinc_fi
UR50_UPI0000D66F9D_pred_simto_Zinc_finger_protein_
UR50_Q4V9E3_Zgc113443__82_Coelomata_Proteins
UR90_UPI0000F1FA2E_Cluster_pred_similar_to_zinc_fi
UR90_UPI0000F21E0C_Cluster_pred_hyp_protein_2_Dani
UR90_UPI0000F1DBB4_Cluster_pred_hyp_protein_1_Dani
UR90_UPI0000F207CE_Cluster_pred_hyp_protein_1_Dani
UR50_UPI0000F20DE1_pred_hyp_protein__1_Danio_rerio
jgi|Dappu1|234903|SNAP_00002961
UR90_Q6PC08_Cluster_Bcell_CLL/lymphoma_6_4_Danio_r
UR90_Q5NRQ2_Cluster_Bcell_lymphoma_6_protein_2_Tak
UR90_Q5U4Y1_Cluster_LOC495412_protein_3_Xenopus_Pr
UR90_Q5ZM39_Cluster_hyp_protein_2_Gallus_gallus_Pr
UR50_P41182_Bcell_lymphoma_6_protein__30_Euteleost
Kruppel
other Zinc-finger proteins
BCL6
1
0.92
0.86
0.96
0.91
1
0
0.99
0.63
0.91
0.97
1
0.23
1
0.93
1
0.6
0.08
0.84
0.24
0.78
0.98
0.6

S13 A

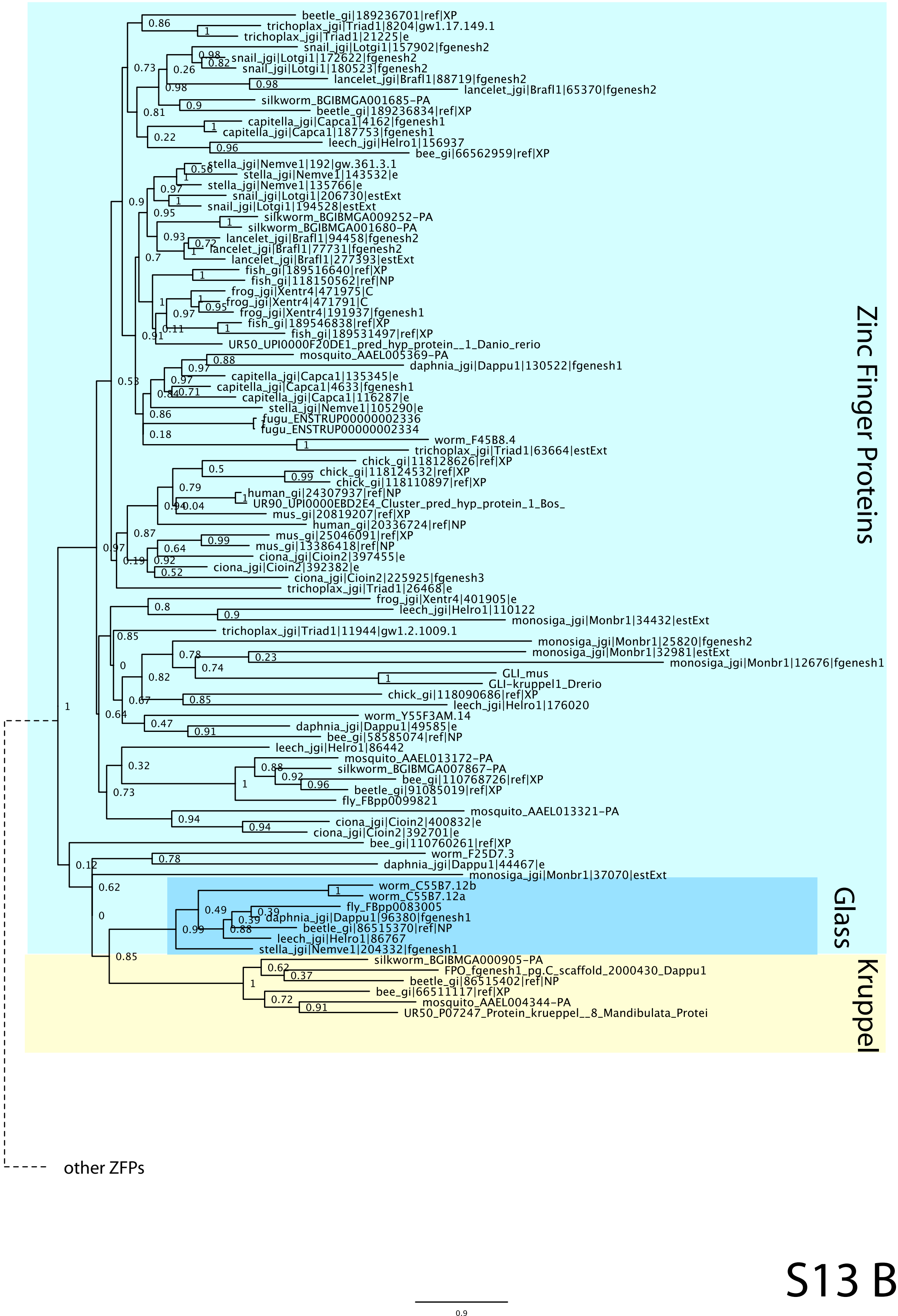
beetle_gi|189236701|ref|XP
trichoplax_jgi|Triad1|8204|gw1.17.149.1
trichoplax_jgi|Triad1|21225|e
snail_jgi|Lotgi1|157902|fgenesh2
snail_jgi|Lotgi1|172622|fgenesh2
snail_jgi|Lotgi1|180523|fgenesh2
lancelet_jgi|Brafl1|88719|fgenesh2
lancelet_jgi|Brafl1|65370|fgenesh2
silkworm_BGIBMGA001685-PA
beetle_gi|189236834|ref|XP
capitella_jgi|Capca1|4162|fgenesh1
capitella_jgi|Capca1|187753|fgenesh1
leech_jgi|Helro1|156937
bee_gi|66562959|ref|XP
stella_jgi|Nemve1|192|gw.361.3.1
stella_jgi|Nemve1|143532|e
stella_jgi|Nemve1|135766|e
snail_jgi|Lotgi1|206730|estExt
snail_jgi|Lotgi1|194528|estExt
silkworm_BGIBMGA009252-PA
silkworm_BGIBMGA001680-PA
lancelet_jgi|Brafl1|94458|fgenesh2
lancelet_jgi|Brafl1|77731|fgenesh2
lancelet_jgi|Brafl1|277393|estExt
fish_gi|189516640|ref|XP
fish_gi|118150562|ref|NP
frog_jgi|Xentr4|471975|C
frog_jgi|Xentr4|471791|C
frog_jgi|Xentr4|191937|fgenesh1
fish_gi|189546838|ref|XP
fish_gi|189531497|ref|XP
UR50_UPI0000F20DE1_pred_hyp_protein__1_Danio_rerio
mosquito_AAEL005369-PA
daphnia_jgi|Dappu1|130522|fgenesh1
capitella_jgi|Capca1|135345|e
capitella_jgi|Capca1|4633|fgenesh1
capitella_jgi|Capca1|116287|e
stella_jgi|Nemve1|105290|e
fugu_ENSTRUP00000002336
fugu_ENSTRUP00000002334
worm_F45B8.4
trichoplax_jgi|Triad1|63664|estExt
chick_gi|118128626|ref|XP
chick_gi|118124532|ref|XP
chick_gi|118110897|ref|XP
human_gi|24307937|ref|NP
UR90_UPI0000EBD2E4_Cluster_pred_hyp_protein_1_Bos_
mus_gi|20819207|ref|XP
human_gi|20336724|ref|NP
mus_gi|25046091|ref|XP
mus_gi|13386418|ref|NP
ciona_jgi|Cioin2|397455|e
ciona_jgi|Cioin2|392382|e
ciona_jgi|Cioin2|225925|fgenesh3
trichoplax_jgi|Triad1|26468|e
frog_jgi|Xentr4|401905|e
leech_jgi|Helro1|110122
monosiga_jgi|Monbr1|34432|estExt
trichoplax_jgi|Triad1|11944|gw1.2.1009.1
monosiga_jgi|Monbr1|25820|fgenesh2
monosiga_jgi|Monbr1|32981|estExt
monosiga_jgi|Monbr1|12676|fgenesh1
GLI_mus
GLI-kruppel1_Drerio
chick_gi|118090686|ref|XP
leech_jgi|Helro1|176020
worm_Y55F3AM.14
daphnia_jgi|Dappu1|49585|e
bee_gi|58585074|ref|NP
leech_jgi|Helro1|86442
mosquito_AAEL013172-PA
silkworm_BGIBMGA007867-PA
bee_gi|110768726|ref|XP
beetle_gi|91085019|ref|XP
fly_FBpp0099821
mosquito_AAEL013321-PA
ciona_jgi|Cioin2|400832|e
ciona_jgi|Cioin2|392701|e
bee_gi|110760261|ref|XP
worm_F25D7.3
daphnia_jgi|Dappu1|44467|e
monosiga_jgi|Monbr1|37070|estExt
worm_C55B7.12b
worm_C55B7.12a
fly_FBpp0083005
daphnia_jgi|Dappu1|96380|fgenesh1
beetle_gi|86515370|ref|NP
leech_jgi|Helro1|86767
stella_jgi|Nemve1|204332|fgenesh1
silkworm_BGIBMGA000905-PA
FPO_fgenesh1_pg.C_scaffold_2000430_Dappu1
beetle_gi|86515402|ref|NP
bee_gi|66511117|ref|XP
mosquito_AAEL004344-PA
UR50_P07247_Protein_krueppel__8_Mandibulata_Protei
Zinc Finger Proteins
Glass
Kruppel
other ZFPs
0.9

S13 B

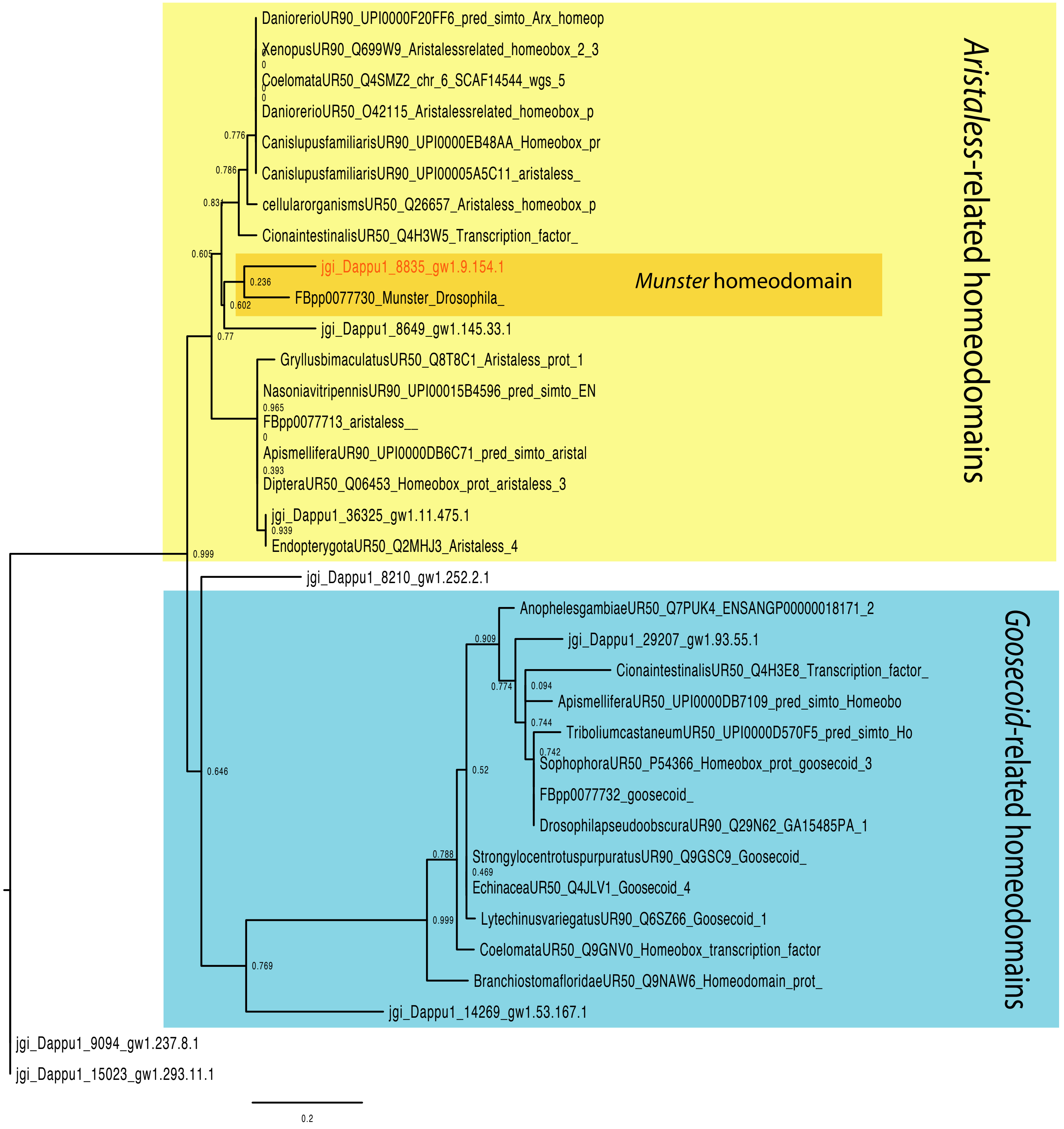
DaniorerioUR90_UPI0000F20FF6_pred_simto_Arx_homeop
XenopusUR90_Q699W9_Aristalessrelated_homeobox_2_3
CoelomataUR50_Q4SMZ2_chr_6_SCAF14544_wgs_5
DaniorerioUR50_O42115_Aristalessrelated_homeobox_p
CanislupusfamiliarisUR90_UPI0000EB48AA_Homeobox_pr
CanislupusfamiliarisUR90_UPI00005A5C11_aristaless_
cellularorganismsUR50_Q26657_Aristaless_homeobox_p
CionaintestinalisUR50_Q4H3W5_Transcription_factor_
jgi_Dappu1_8835_gw1.9.154.1
FBpp0077730_Munster_Drosophila_
Munster homeodomain
jgi_Dappu1_8649_gw1.145.33.1
GryllusbimaculatusUR50_Q8T8C1_Aristaless_prot_1
NasoniavitripennisUR90_UPI00015B4596_pred_simto_EN
FBpp0077713_aristaless__
ApismelliferaUR90_UPI0000DB6C71_pred_simto_aristal
DipteraUR50_Q06453_Homeobox_prot_aristaless_3
jgi_Dappu1_36325_gw1.11.475.1
EndopterygotaUR50_Q2MHJ3_Aristaless_4
Aristaless-related homeodomains
jgi_Dappu1_8210_gw1.252.2.1
AnophelesgambiaeUR50_Q7PUK4_ENSANGP00000018171_2
jgi_Dappu1_29207_gw1.93.55.1
CionaintestinalisUR50_Q4H3E8_Transcription_factor_
ApismelliferaUR50_UPI0000DB7109_pred_simto_Homeobo
TriboliumcastaneumUR50_UPI0000D570F5_pred_simto_Ho
SophophoraUR50_P54366_Homeobox_prot_goosecoid_3
FBpp0077732_goosecoid_
DrosophilapseudoobscuraUR90_Q29N62_GA15485PA_1
StrongylocentrotuspurpuratusUR90_Q9GSC9_Goosecoid_
EchinaceaUR50_Q4JLV1_Goosecoid_4
LytechinusvariegatusUR90_Q6SZ66_Goosecoid_1
CoelomataUR50_Q9GNV0_Homeobox_transcription_factor
BranchiostomafloridaeUR50_Q9NAW6_Homeodomain_prot_
jgi_Dappu1_14269_gw1.53.167.1
Goosecoid-related homeodomains
jgi_Dappu1_9094_gw1.237.8.1
jgi_Dappu1_15023_gw1.293.11.1
0.776
0.786
0.83
0.605
0.236
0.602
0.77
0.965
0.393
0.939
0.999
0.909
0.774
0.094
0.744
0.742
0.52
0.788
0.469
0.999
0.646
0.769
0.2

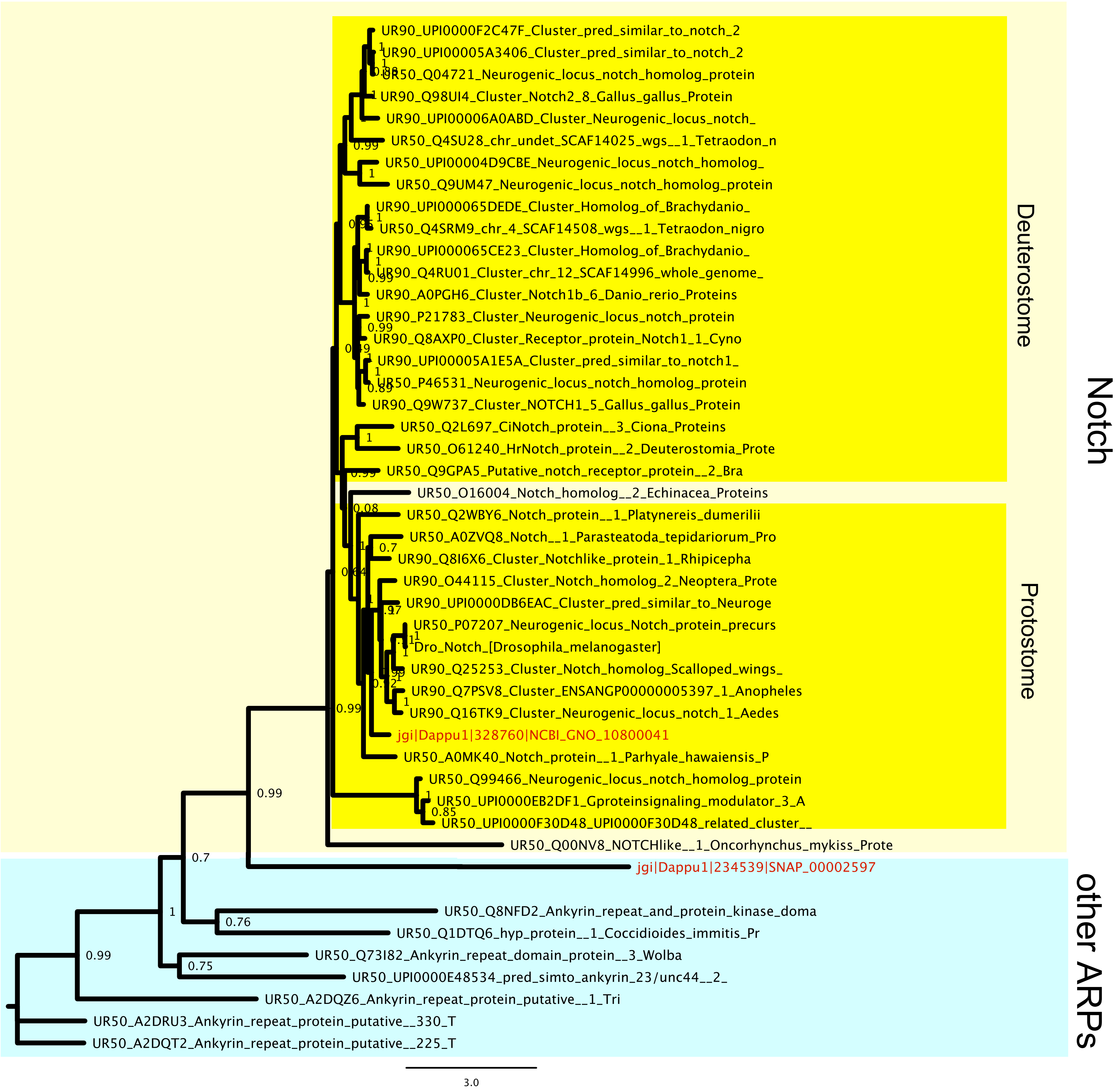

UR90_UPI0000F2C47F_Cluster_pred_similar_to_notch_2
UR90_UPI00005A3406_Cluster_pred_similar_to_notch_2
UR50_Q04721_Neurogenic_locus_notch_homolog_protein
UR90_Q98UI4_Cluster_Notch2_8_Gallus_gallus_Protein
UR90_UPI00006A0ABD_Cluster_Neurogenic_locus_notch_
UR50_Q4SU28_chr_undet_SCAF14025_wgs__1_Tetraodon_n
UR50_UPI00004D9CBE_Neurogenic_locus_notch_homolog_
UR50_Q9UM47_Neurogenic_locus_notch_homolog_protein
UR90_UPI000065DEDE_Cluster_Homolog_of_Brachydanio_
UR50_Q4SRM9_chr_4_SCAF14508_wgs__1_Tetraodon_nigro
UR90_UPI000065CE23_Cluster_Homolog_of_Brachydanio_
UR90_Q4RU01_Cluster_chr_12_SCAF14996_whole_genome_
UR90_A0PGH6_Cluster_Notch1b_6_Danio_rerio_Proteins
UR90_P21783_Cluster_Neurogenic_locus_notch_protein
UR90_Q8AXP0_Cluster_Receptor_protein_Notch1_1_Cyno
UR90_UPI00005A1E5A_Cluster_pred_similar_to_notch1_
UR50_P46531_Neurogenic_locus_notch_homolog_protein
UR90_Q9W737_Cluster_NOTCH1_5_Gallus_gallus_Protein
UR50_Q2L697_CiNotch_protein__3_Ciona_Proteins
UR50_O61240_HrNotch_protein__2_Deuterostomia_Prote
UR50_Q9GPA5_Putative_notch_receptor_protein__2_Bra
UR50_O16004_Notch_homolog__2_Echinacea_Proteins
UR50_Q2WBY6_Notch_protein__1_Platynereis_dumerilii
UR50_A0ZVQ8_Notch__1_Parasteatoda_tepidariorum_Pro
UR90_Q8I6X6_Cluster_Notchlike_protein_1_Rhipicepha
UR90_O44115_Cluster_Notch_homolog_2_Neoptera_Prote
UR90_UPI0000DB6EAC_Cluster_pred_similar_to_Neuroge
UR50_P07207_Neurogenic_locus_Notch_protein_precurs
Dro_Notch_[Drosophila_melanogaster]
UR90_Q25253_Cluster_Notch_homolog_Scalloped_wings_
UR90_Q7PSV8_Cluster_ENSANGP00000005397_1_Anopheles
UR90_Q16TK9_Cluster_Neurogenic_locus_notch_1_Aedes
jgi|Dappu1|328760|NCBI_GNO_10800041
UR50_A0MK40_Notch_protein__1_Parhyale_hawaiensis_P
UR50_Q99466_Neurogenic_locus_notch_homolog_protein
UR50_UPI0000EB2DF1_Gproteinsignaling_modulator_3_A
UR50_UPI0000F30D48_UPI0000F30D48_related_cluster__
UR50_Q00NV8_NOTCHlike__1_Oncorhynchus_mykiss_Prote
jgi|Dappu1|234539|SNAP_00002597
UR50_Q8NFD2_Ankyrin_repeat_and_protein_kinase_doma
UR50_Q1DTQ6_hyp_protein__1_Coccidioides_immitis_Pr
UR50_Q73I82_Ankyrin_repeat_domain_protein__3_Wolba
UR50_UPI0000E48534_pred_simto_ankyrin_23/unc44__2_
UR50_A2DQZ6_Ankyrin_repeat_protein_putative__1_Tri
UR50_A2DRU3_Ankyrin_repeat_protein_putative__330_T
UR50_A2DQT2_Ankyrin_repeat_protein_putative__225_T
Deuterostome
Protostome
Notch
other ARPs
3.0

S15 A

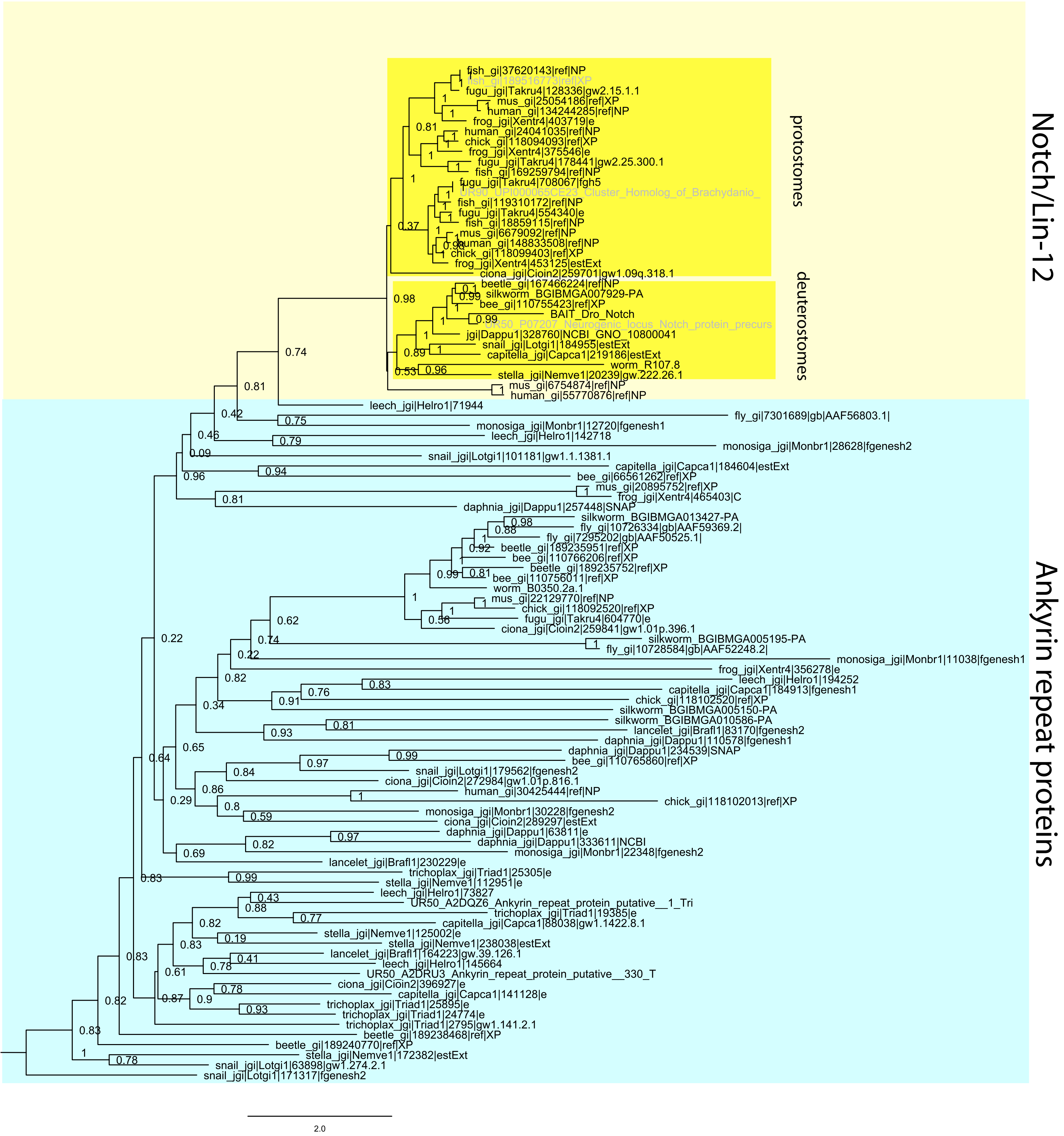
Notch/Lin-12
protostomes
deuterostomes
Ankyrin repeat proteins
fish_gi|37620143|ref|NP
fish_gi|189516773|ref|XP
fugu_jgi|Takru4|128336|gw2.15.1.1
mus_gi|25054186|ref|XP
human_gi|134244285|ref|NP
frog_jgi|Xentr4|403719|e
human_gi|24041035|ref|NP
chick_gi|118094093|ref|XP
frog_jgi|Xentr4|375546|e
fugu_jgi|Takru4|178441|gw2.25.300.1
fish_gi|169259794|ref|NP
fugu_jgi|Takru4|708067|fgh5
UR90_UPI000065CE23_Cluster_Homolog_of_Brachydanio_
fish_gi|119310172|ref|NP
fugu_jgi|Takru4|554340|e
fish_gi|18859115|ref|NP
mus_gi|6679092|ref|NP
human_gi|148833508|ref|NP
chick_gi|118099403|ref|XP
frog_jgi|Xentr4|453125|estExt
ciona_jgi|Cioin2|259701|gw1.09q.318.1
beetle_gi|167466224|ref|NP
silkworm_BGIBMGA007929-PA
bee_gi|110755423|ref|XP
BAIT_Dro_Notch
UR50_P07207_Neurogenic_locus_Notch_protein_precurs
jgi|Dappu1|328760|NCBI_GNO_10800041
snail_jgi|Lotgi1|184955|estExt
capitella_jgi|Capca1|219186|estExt
worm_R107.8
stella_jgi|Nemve1|20239|gw.222.26.1
mus_gi|6754874|ref|NP
human_gi|55770876|ref|NP
leech_jgi|Helro1|71944
fly_gi|7301689|gb|AAF56803.1|
monosiga_jgi|Monbr1|12720|fgenesh1
leech_jgi|Helro1|142718
monosiga_jgi|Monbr1|28628|fgenesh2
snail_jgi|Lotgi1|101181|gw1.1.1381.1
capitella_jgi|Capca1|184604|estExt
bee_gi|66561262|ref|XP
mus_gi|20895752|ref|XP
frog_jgi|Xentr4|465403|C
daphnia_jgi|Dappu1|257448|SNAP
silkworm_BGIBMGA013427-PA
fly_gi|10726334|gb|AAF59369.2|
fly_gi|7295202|gb|AAF50525.1|
beetle_gi|189235951|ref|XP
bee_gi|110766206|ref|XP
beetle_gi|189235752|ref|XP
bee_gi|110756011|ref|XP
worm_B0350.2a.1
mus_gi|22129770|ref|NP
chick_gi|118092520|ref|XP
fugu_jgi|Takru4|604770|e
ciona_jgi|Cioin2|259841|gw1.01p.396.1
silkworm_BGIBMGA005195-PA
fly_gi|10728584|gb|AAF52248.2|
monosiga_jgi|Monbr1|11038|fgenesh1
frog_jgi|Xentr4|356278|e
leech_jgi|Helro1|194252
capitella_jgi|Capca1|184913|fgenesh1
chick_gi|118102520|ref|XP
silkworm_BGIBMGA005150-PA
silkworm_BGIBMGA010586-PA
lancelet_jgi|Brafl1|83170|fgenesh2
daphnia_jgi|Dappu1|110578|fgenesh1
daphnia_jgi|Dappu1|234539|SNAP
bee_gi|110765860|ref|XP
snail_jgi|Lotgi1|179562|fgenesh2
ciona_jgi|Cioin2|272984|gw1.01p.816.1
human_gi|30425444|ref|NP
chick_gi|118102013|ref|XP
monosiga_jgi|Monbr1|30228|fgenesh2
ciona_jgi|Cioin2|289297|estExt
daphnia_jgi|Dappu1|63811|e
daphnia_jgi|Dappu1|333611|NCBI
monosiga_jgi|Monbr1|22348|fgenesh2
lancelet_jgi|Brafl1|230229|e
trichoplax_jgi|Triad1|25305|e
stella_jgi|Nemve1|112951|e
leech_jgi|Helro1|73827
UR50_A2DQZ6_Ankyrin_repeat_protein_putative__1_Tri
trichoplax_jgi|Triad1|19385|e
capitella_jgi|Capca1|88038|gw1.1422.8.1
stella_jgi|Nemve1|125002|e
stella_jgi|Nemve1|238038|estExt
lancelet_jgi|Brafl1|164223|gw.39.126.1
leech_jgi|Helro1|145664
UR50_A2DRU3_Ankyrin_repeat_protein_putative__330_T
ciona_jgi|Cioin2|396927|e
capitella_jgi|Capca1|141128|e
trichoplax_jgi|Triad1|25895|e
trichoplax_jgi|Triad1|24774|e
trichoplax_jgi|Triad1|2795|gw1.141.2.1
beetle_gi|189238468|ref|XP
beetle_gi|189240770|ref|XP
stella_jgi|Nemve1|172382|estExt
snail_jgi|Lotgi1|63898|gw1.274.2.1
snail_jgi|Lotgi1|171317|fgenesh2
2.0

S15 B

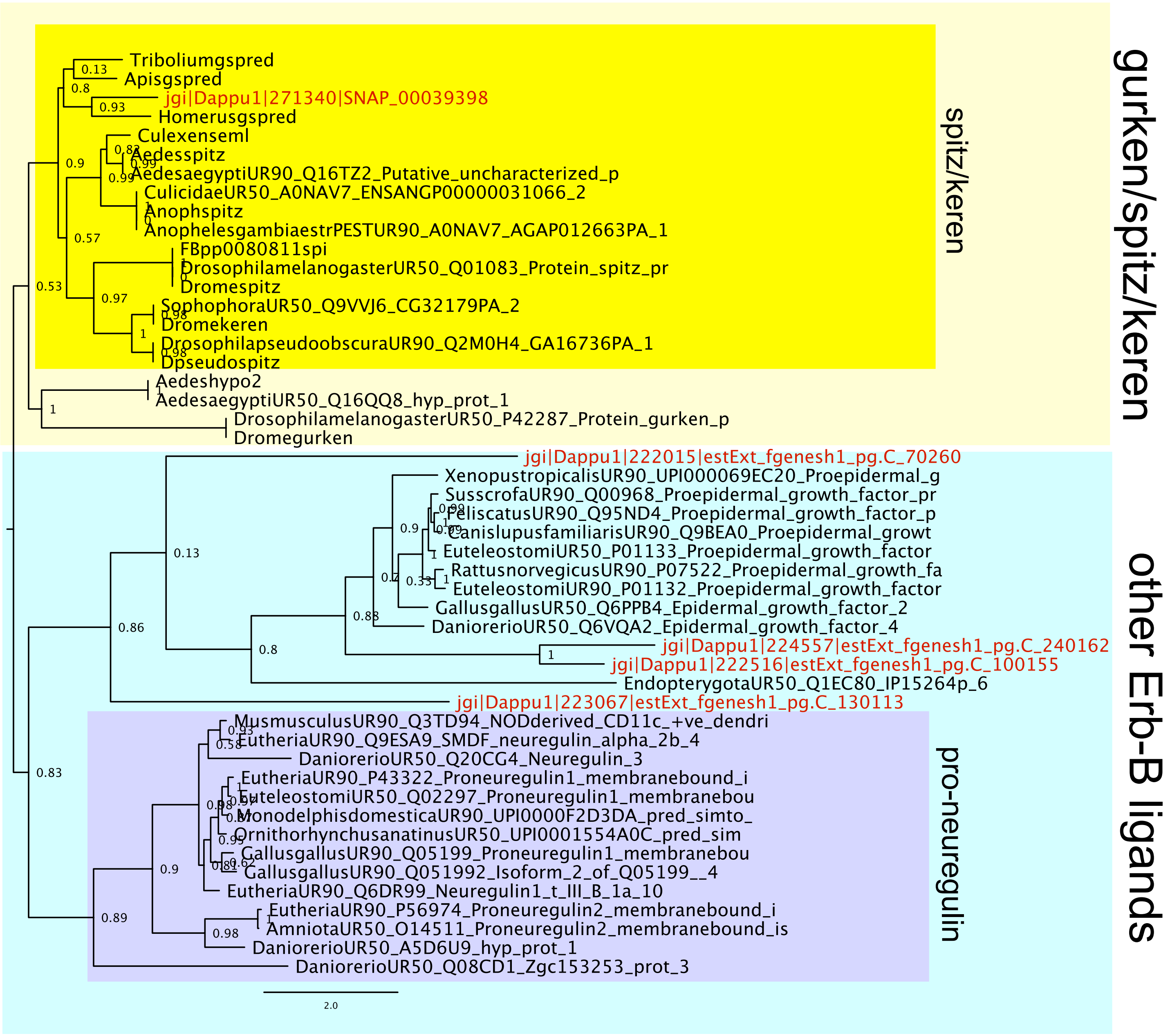

Triboliumgspred
Apisgspred
jgi|Dappu1|271340|SNAP_00039398
Homerusgspred
Culexenseml
Aedesspitz
AedesaegyptiUR90_Q16TZ2_Putative_uncharacterized_p
CulicidaeUR50_A0NAV7_ENSANGP00000031066_2
Anophspitz
AnophelesgambiaestrPESTUR90_A0NAV7_AGAP012663PA_1
FBpp0080811spi
DrosophilamelanogasterUR50_Q01083_Protein_spitz_pr
Dromespitz
SophophoraUR50_Q9VVJ6_CG32179PA_2
Dromekeren
DrosophilapseudoobscuraUR90_Q2M0H4_GA16736PA_1
Dpseudospitz
Aedeshypo2
AedesaegyptiUR50_Q16QQ8_hyp_prot_1
DrosophilamelanogasterUR50_P42287_Protein_gurken_p
Dromegurken
jgi|Dappu1|222015|estExt_fgenesh1_pg.C_70260
XenopustropicalisUR90_UPI000069EC20_Proepidermal_g
SusscrofaUR90_Q00968_Proepidermal_growth_factor_pr
FeliscatusUR90_Q95ND4_Proepidermal_growth_factor_p
CanislupusfamiliarisUR90_Q9BEA0_Proepidermal_growt
EuteleostomiUR50_P01133_Proepidermal_growth_factor
RattusnorvegicusUR90_P07522_Proepidermal_growth_fa
EuteleostomiUR90_P01132_Proepidermal_growth_factor
GallusgallusUR50_Q6PPB4_Epidermal_growth_factor_2
DaniorerioUR50_Q6VQA2_Epidermal_growth_factor_4
jgi|Dappu1|224557|estExt_fgenesh1_pg.C_240162
jgi|Dappu1|222516|estExt_fgenesh1_pg.C_100155
EndopterygotaUR50_Q1EC80_IP15264p_6
jgi|Dappu1|223067|estExt_fgenesh1_pg.C_130113
MusmusculusUR90_Q3TD94_NODderived_CD11c_+ve_dendri
EutheriaUR90_Q9ESA9_SMDF_neuregulin_alpha_2b_4
DaniorerioUR50_Q20CG4_Neuregulin_3
EutheriaUR90_P43322_Proneuregulin1_membranebound_i
EuteleostomiUR50_Q02297_Proneuregulin1_membranebou
MonodelphisdomesticaUR90_UPI0000F2D3DA_pred_simto_
OrnithorhynchusanatinusUR50_UPI0001554A0C_pred_sim
GallusgallusUR90_Q05199_Proneuregulin1_membranebou
GallusgallusUR90_Q051992_Isoform_2_of_Q05199__4
EutheriaUR90_Q6DR99_Neuregulin1_t_III_B_1a_10
EutheriaUR90_P56974_Proneuregulin2_membranebound_i
AmniotaUR50_O14511_Proneuregulin2_membranebound_is
DaniorerioUR50_A5D6U9_hyp_prot_1
DaniorerioUR50_Q08CD1_Zgc153253_prot_3
2.0
spitz/keren
gurken/spitz/keren
pro-neuregulin
other Erb-B ligands

S16 A

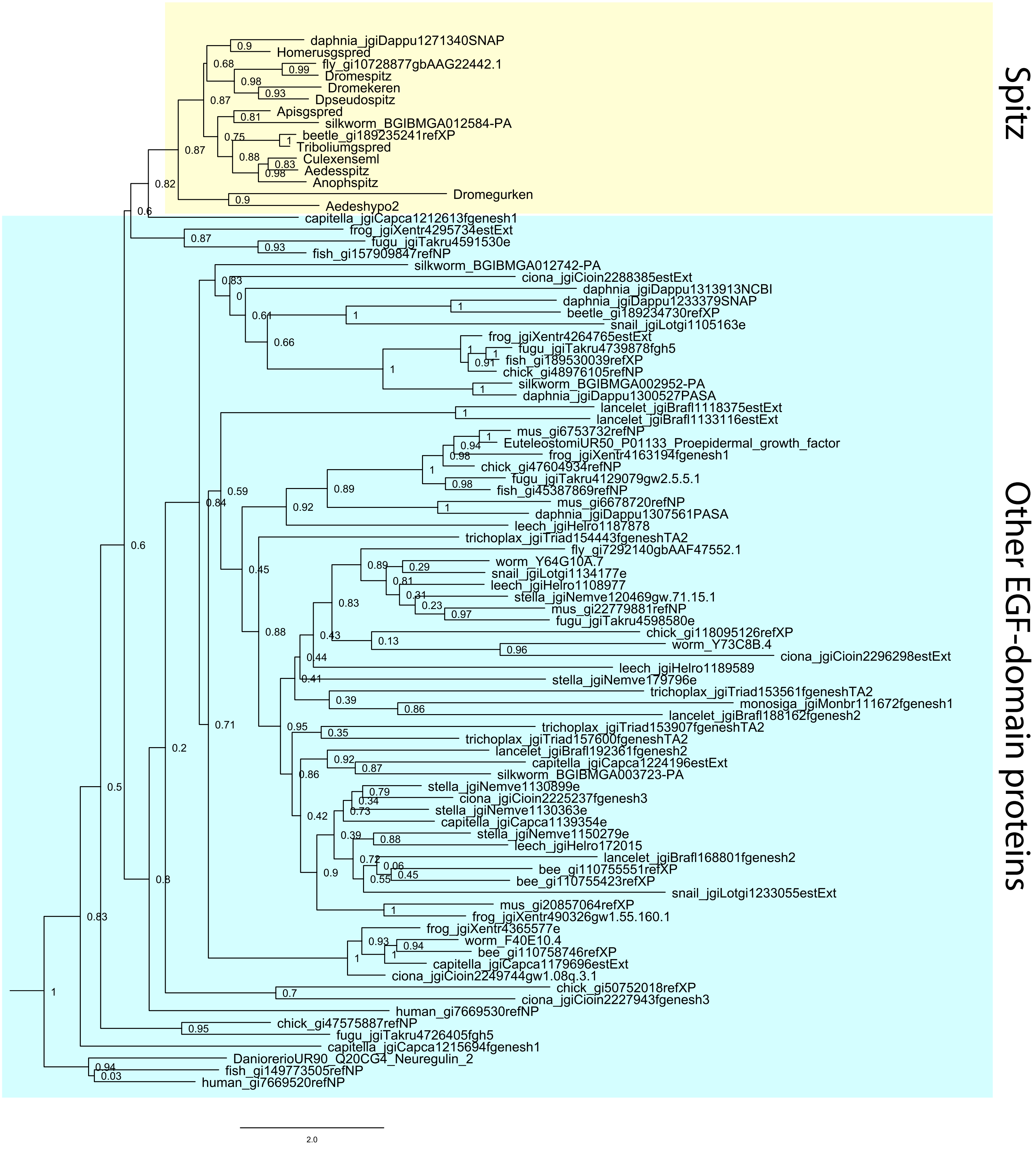
Spitz
Other EGF-domain proteins
daphnia_jgiDappu1271340SNAP
Homerusgspred
fly_gi10728877gbAAG22442.1
Dromespitz
Dromekeren
Dpseudospitz
Apisgspred
silkworm_BGIBMGA012584-PA
beetle_gi189235241refXP
Triboliumgspred
Culexenseml
Aedesspitz
Anophspitz
Dromegurken
Aedeshypo2
capitella_jgiCapca1212613fgenesh1
frog_jgiXentr4295734estExt
fugu_jgiTakru4591530e
fish_gi157909847refNP
silkworm_BGIBMGA012742-PA
ciona_jgiCioin2288385estExt
daphnia_jgiDappu1313913NCBI
daphnia_jgiDappu1233379SNAP
beetle_gi189234730refXP
snail_jgiLotgi1105163e
frog_jgiXentr4264765estExt
fugu_jgiTakru4739878fgh5
fish_gi189530039refXP
chick_gi48976105refNP
silkworm_BGIBMGA002952-PA
daphnia_jgiDappu1300527PASA
lancelet_jgiBrafl1118375estExt
lancelet_jgiBrafl1133116estExt
mus_gi6753732refNP
EuteleostomiUR50_P01133_Proepidermal_growth_factor
frog_jgiXentr4163194fgenesh1
chick_gi47604934refNP
fugu_jgiTakru4129079gw2.5.5.1
fish_gi45387869refNP
mus_gi6678720refNP
daphnia_jgiDappu1307561PASA
leech_jgiHelro1187878
trichoplax_jgiTriad154443fgeneshTA2
fly_gi7292140gbAAF47552.1
worm_Y64G10A.7
snail_jgiLotgi1134177e
leech_jgiHelro1108977
stella_jgiNemve120469gw.71.15.1
mus_gi22779881refNP
fugu_jgiTakru4598580e
chick_gi118095126refXP
worm_Y73C8B.4
ciona_jgiCioin2296298estExt
leech_jgiHelro1189589
stella_jgiNemve179796e
trichoplax_jgiTriad153561fgeneshTA2
monosiga_jgiMonbr111672fgenesh1
lancelet_jgiBrafl188162fgenesh2
trichoplax_jgiTriad153907fgeneshTA2
trichoplax_jgiTriad157600fgeneshTA2
lancelet_jgiBrafl192361fgenesh2
capitella_jgiCapca1224196estExt
silkworm_BGIBMGA003723-PA
stella_jgiNemve1130899e
ciona_jgiCioin2225237fgenesh3
stella_jgiNemve1130363e
capitella_jgiCapca1139354e
stella_jgiNemve1150279e
leech_jgiHelro172015
lancelet_jgiBrafl168801fgenesh2
bee_gi110755551refXP
bee_gi110755423refXP
snail_jgiLotgi1233055estExt
mus_gi20857064refXP
frog_jgiXentr490326gw1.55.160.1
frog_jgiXentr4365577e
worm_F40E10.4
bee_gi110758746refXP
capitella_jgiCapca1179696estExt
ciona_jgiCioin2249744gw1.08q.3.1
chick_gi50752018refXP
ciona_jgiCioin2227943fgenesh3
human_gi7669530refNP
chick_gi47575887refNP
fugu_jgiTakru4726405fgh5
capitella_jgiCapca1215694fgenesh1
DaniorerioUR90_Q20CG4_Neuregulin_2
fish_gi149773505refNP
human_gi7669520refNP
2.0

S16 B

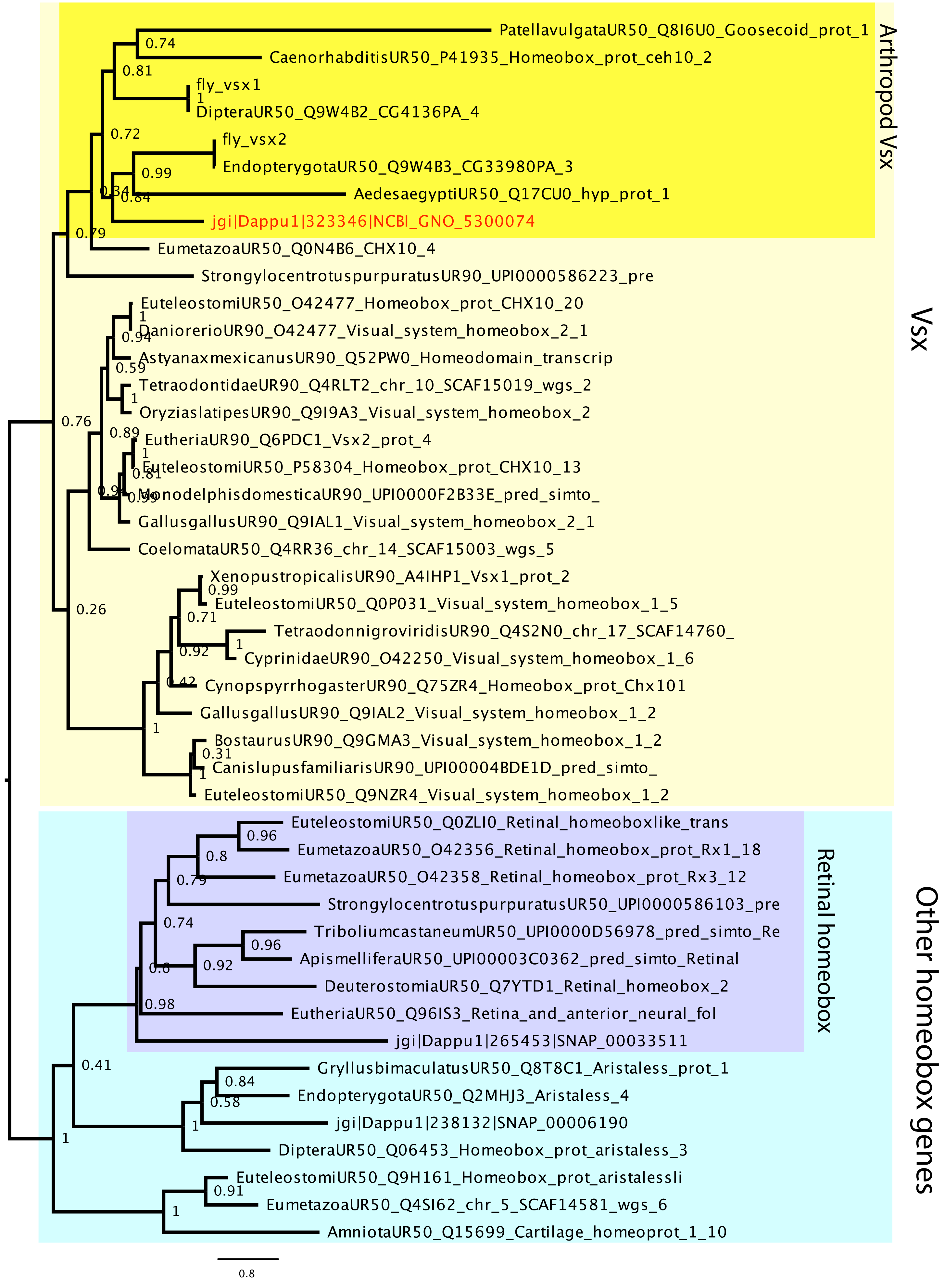

PatellavulgataUR50_Q8I6U0_Goosecoid_prot_1
CaenorhabditisUR50_P41935_Homeobox_prot_ceh10_2
fly_vsx1
DipteraUR50_Q9W4B2_CG4136PA_4
fly_vsx2
EndopterygotaUR50_Q9W4B3_CG33980PA_3
AedesaegyptiUR50_Q17CU0_hyp_prot_1
jgi|Dappu1|323346|NCBI_GNO_5300074
EumetazoaUR50_Q0N4B6_CHX10_4
StrongylocentrotuspurpuratusUR90_UPI0000586223_pre
EuteleostomiUR50_O42477_Homeobox_prot_CHX10_20
DaniorerioUR90_O42477_Visual_system_homeobox_2_1
AstyanaxmexicanusUR90_Q52PW0_Homeodomain_transcrip
TetraodontidaeUR90_Q4RLT2_chr_10_SCAF15019_wgs_2
OryziaslatipesUR90_Q9I9A3_Visual_system_homeobox_2
EutheriaUR90_Q6PDC1_Vsx2_prot_4
EuteleostomiUR50_P58304_Homeobox_prot_CHX10_13
MonodelphisdomesticaUR90_UPI0000F2B33E_pred_simto_
GallusgallusUR90_Q9IAL1_Visual_system_homeobox_2_1
CoelomataUR50_Q4RR36_chr_14_SCAF15003_wgs_5
XenopustropicalisUR90_A4IHP1_Vsx1_prot_2
EuteleostomiUR50_Q0P031_Visual_system_homeobox_1_5
TetraodonnigroviridisUR90_Q4S2N0_chr_17_SCAF14760_
CyprinidaeUR90_O42250_Visual_system_homeobox_1_6
CynopspyrrhogasterUR90_Q75ZR4_Homeobox_prot_Chx101
GallusgallusUR90_Q9IAL2_Visual_system_homeobox_1_2
BostaurusUR90_Q9GMA3_Visual_system_homeobox_1_2
CanislupusfamiliarisUR90_UPI00004BDE1D_pred_simto_
EuteleostomiUR50_Q9NZR4_Visual_system_homeobox_1_2
EuteleostomiUR50_Q0ZLI0_Retinal_homeoboxlike_trans
EumetazoaUR50_O42356_Retinal_homeobox_prot_Rx1_18
EumetazoaUR50_O42358_Retinal_homeobox_prot_Rx3_12
StrongylocentrotuspurpuratusUR50_UPI0000586103_pre
TriboliumcastaneumUR50_UPI0000D56978_pred_simto_Re
ApismelliferaUR50_UPI00003C0362_pred_simto_Retinal
DeuterostomiaUR50_Q7YTD1_Retinal_homeobox_2
EutheriaUR50_Q96IS3_Retina_and_anterior_neural_fol
jgi|Dappu1|265453|SNAP_00033511
GryllusbimaculatusUR50_Q8T8C1_Aristaless_prot_1
EndopterygotaUR50_Q2MHJ3_Aristaless_4
jgi|Dappu1|238132|SNAP_00006190
DipteraUR50_Q06453_Homeobox_prot_aristaless_3
EuteleostomiUR50_Q9H161_Homeobox_prot_aristalessli
EumetazoaUR50_Q4SI62_chr_5_SCAF14581_wgs_6
AmniotaUR50_Q15699_Cartilage_homeoprot_1_10
Arthropod Vsx
Vsx
Retinal homeobox
Other homeobox genes
0.8

S17 A

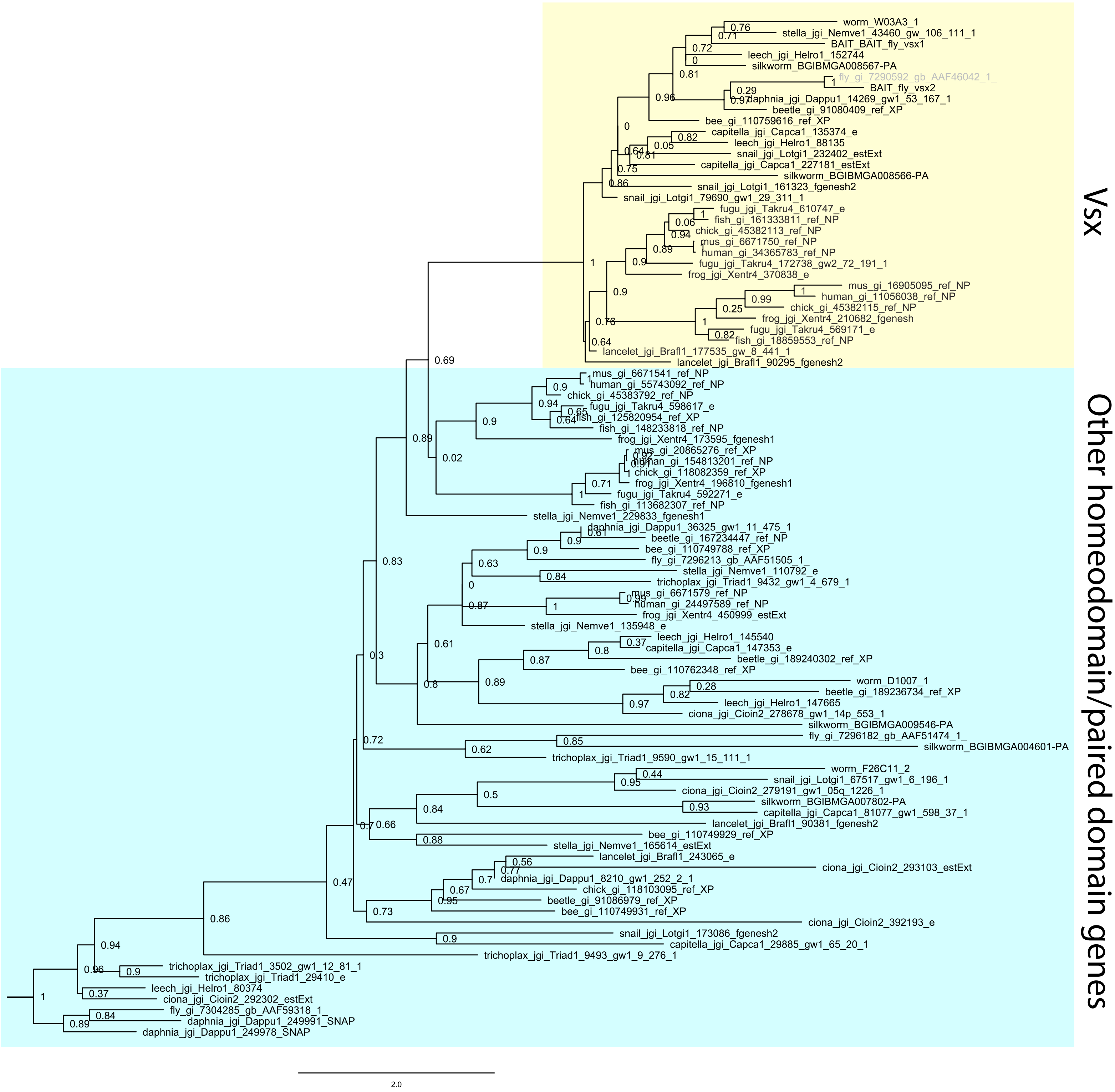
Vsx
Other homeodomain/paired domain genes
worm_W03A3_1
stella_jgi_Nemve1_43460_gw_106_111_1
BAIT_BAIT_fly_vsx1
leech_jgi_Helro1_152744
silkworm_BGIBMGA008567-PA
fly_gi_7290592_gb_AAF46042_1_
BAIT_fly_vsx2
daphnia_jgi_Dappu1_14269_gw1_53_167_1
beetle_gi_91080409_ref_XP
bee_gi_110759616_ref_XP
capitella_jgi_Capca1_135374_e
leech_jgi_Helro1_88135
snail_jgi_Lotgi1_232402_estExt
capitella_jgi_Capca1_227181_estExt
silkworm_BGIBMGA008566-PA
snail_jgi_Lotgi1_161323_fgenesh2
snail_jgi_Lotgi1_79690_gw1_29_311_1
fugu_jgi_Takru4_610747_e
fish_gi_161333811_ref_NP
chick_gi_45382113_ref_NP
mus_gi_6671750_ref_NP
human_gi_34365783_ref_NP
fugu_jgi_Takru4_172738_gw2_72_191_1
frog_jgi_Xentr4_370838_e
mus_gi_16905095_ref_NP
human_gi_11056038_ref_NP
chick_gi_45382115_ref_NP
frog_jgi_Xentr4_210682_fgenesh
fugu_jgi_Takru4_569171_e
fish_gi_18859553_ref_NP
lancelet_jgi_Brafl1_177535_gw_8_441_1
lancelet_jgi_Brafl1_90295_fgenesh2
mus_gi_6671541_ref_NP
human_gi_55743092_ref_NP
chick_gi_45383792_ref_NP
fugu_jgi_Takru4_598617_e
fish_gi_125820954_ref_XP
fish_gi_148233818_ref_NP
frog_jgi_Xentr4_173595_fgenesh1
mus_gi_20865276_ref_XP
human_gi_154813201_ref_NP
chick_gi_118082359_ref_XP
frog_jgi_Xentr4_196810_fgenesh1
fugu_jgi_Takru4_592271_e
fish_gi_113682307_ref_NP
stella_jgi_Nemve1_229833_fgenesh1
daphnia_jgi_Dappu1_36325_gw1_11_475_1
beetle_gi_167234447_ref_NP
bee_gi_110749788_ref_XP
fly_gi_7296213_gb_AAF51505_1_
stella_jgi_Nemve1_110792_e
trichoplax_jgi_Triad1_9432_gw1_4_679_1
mus_gi_6671579_ref_NP
human_gi_24497589_ref_NP
frog_jgi_Xentr4_450999_estExt
stella_jgi_Nemve1_135948_e
leech_jgi_Helro1_145540
capitella_jgi_Capca1_147353_e
beetle_gi_189240302_ref_XP
bee_gi_110762348_ref_XP
worm_D1007_1
beetle_gi_189236734_ref_XP
leech_jgi_Helro1_147665
ciona_jgi_Cioin2_278678_gw1_14p_553_1
silkworm_BGIBMGA009546-PA
fly_gi_7296182_gb_AAF51474_1_
silkworm_BGIBMGA004601-PA
trichoplax_jgi_Triad1_9590_gw1_15_111_1
worm_F26C11_2
snail_jgi_Lotgi1_67517_gw1_6_196_1
ciona_jgi_Cioin2_279191_gw1_05q_1226_1
silkworm_BGIBMGA007802-PA
capitella_jgi_Capca1_81077_gw1_598_37_1
lancelet_jgi_Brafl1_90381_fgenesh2
bee_gi_110749929_ref_XP
stella_jgi_Nemve1_165614_estExt
lancelet_jgi_Brafl1_243065_e
ciona_jgi_Cioin2_293103_estExt
daphnia_jgi_Dappu1_8210_gw1_252_2_1
chick_gi_118103095_ref_XP
beetle_gi_91086979_ref_XP
bee_gi_110749931_ref_XP
ciona_jgi_Cioin2_392193_e
snail_jgi_Lotgi1_173086_fgenesh2
capitella_jgi_Capca1_29885_gw1_65_20_1
trichoplax_jgi_Triad1_9493_gw1_9_276_1
trichoplax_jgi_Triad1_3502_gw1_12_81_1
trichoplax_jgi_Triad1_29410_e
leech_jgi_Helro1_80374
ciona_jgi_Cioin2_292302_estExt
fly_gi_7304285_gb_AAF59318_1_
daphnia_jgi_Dappu1_249991_SNAP
daphnia_jgi_Dappu1_249978_SNAP
2.0

S17 B

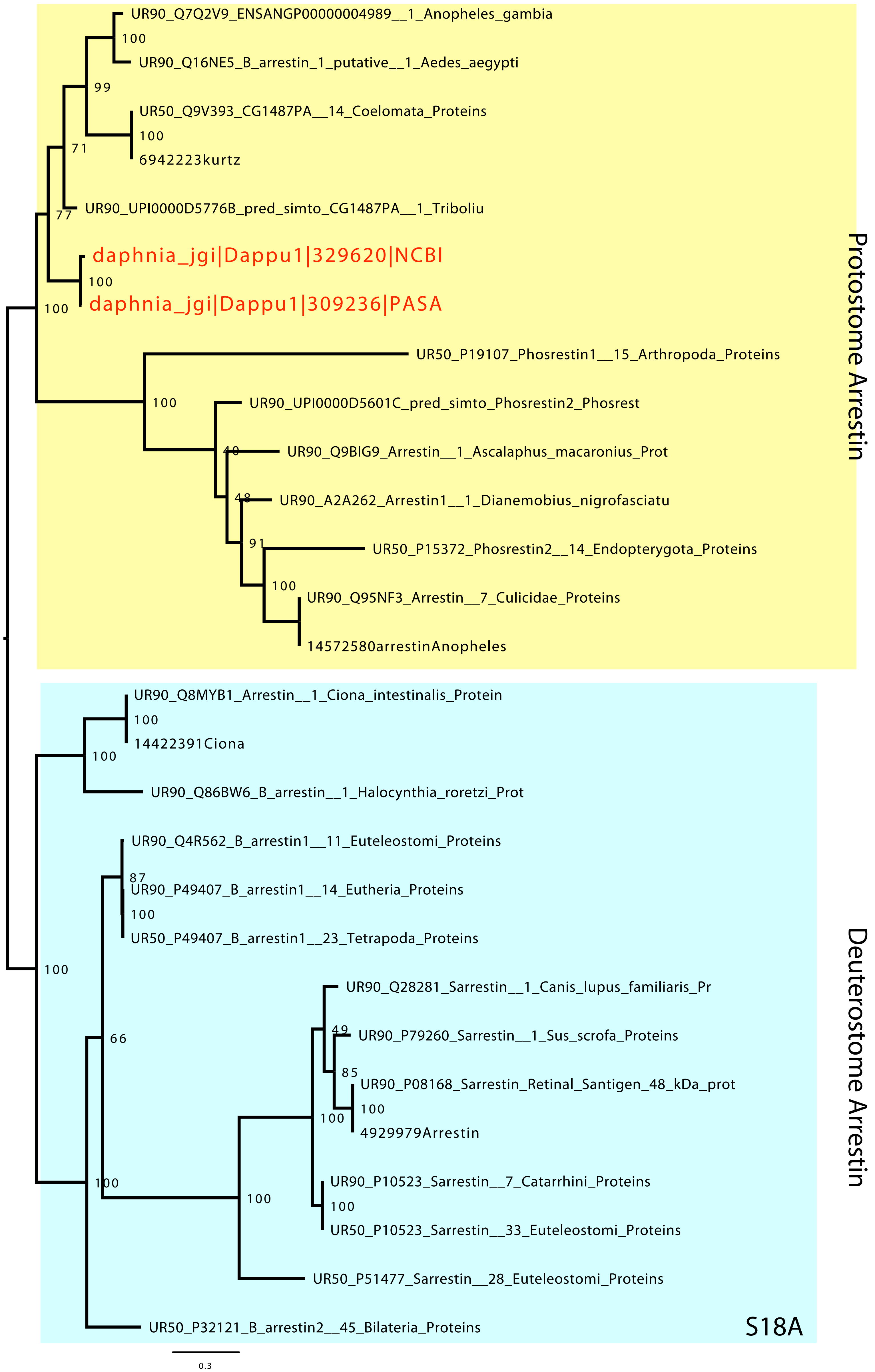
UR90_Q7Q2V9_ENSANGP00000004989__1_Anopheles_gambia
100
UR90_Q16NE5_B_arrestin_1_putative__1_Aedes_aegypti
99
UR50_Q9V393_CG1487PA__14_Coelomata_Proteins
100
71
6942223kurtz
UR90_UPI0000D5776B_pred_simto_CG1487PA__1_Triboliu
77
daphnia_jgi|Dappu1|329620|NCBI
100
daphnia_jgi|Dappu1|309236|PASA
100
UR50_P19107_Phosrestin1__15_Arthropoda_Proteins
100
UR90_UPI0000D5601C_pred_simto_Phosrestin2_Phosrest
40
UR90_Q9BIG9_Arrestin__1_Ascalaphus_macaronius_Prot
48
UR90_A2A262_Arrestin1__1_Dianemobius_nigrofasciatu
91
UR50_P15372_Phosrestin2__14_Endopterygota_Proteins
100
UR90_Q95NF3_Arrestin__7_Culicidae_Proteins
14572580arrestinAnopheles
Protostome Arrestin
UR90_Q8MYB1_Arrestin__1_Ciona_intestinalis_Protein
100
14422391Ciona
100
UR90_Q86BW6_B_arrestin__1_Halocynthia_roretzi_Prot
UR90_Q4R562_B_arrestin1__11_Euteleostomi_Proteins
87
UR90_P49407_B_arrestin1__14_Eutheria_Proteins
100
UR50_P49407_B_arrestin1__23_Tetrapoda_Proteins
100
UR90_Q28281_Sarrestin__1_Canis_lupus_familiaris_Pr
66
49
UR90_P79260_Sarrestin__1_Sus_scrofa_Proteins
85
UR90_P08168_Sarrestin_Retinal_Santigen_48_kDa_prot
100
100
4929979Arrestin
UR90_P10523_Sarrestin__7_Catarrhini_Proteins
100
100
100
UR50_P10523_Sarrestin__33_Euteleostomi_Proteins
UR50_P51477_Sarrestin__28_Euteleostomi_Proteins
UR50_P32121_B_arrestin2__45_Bilateria_Proteins
Deuterostome Arrestin
S18A
0.3

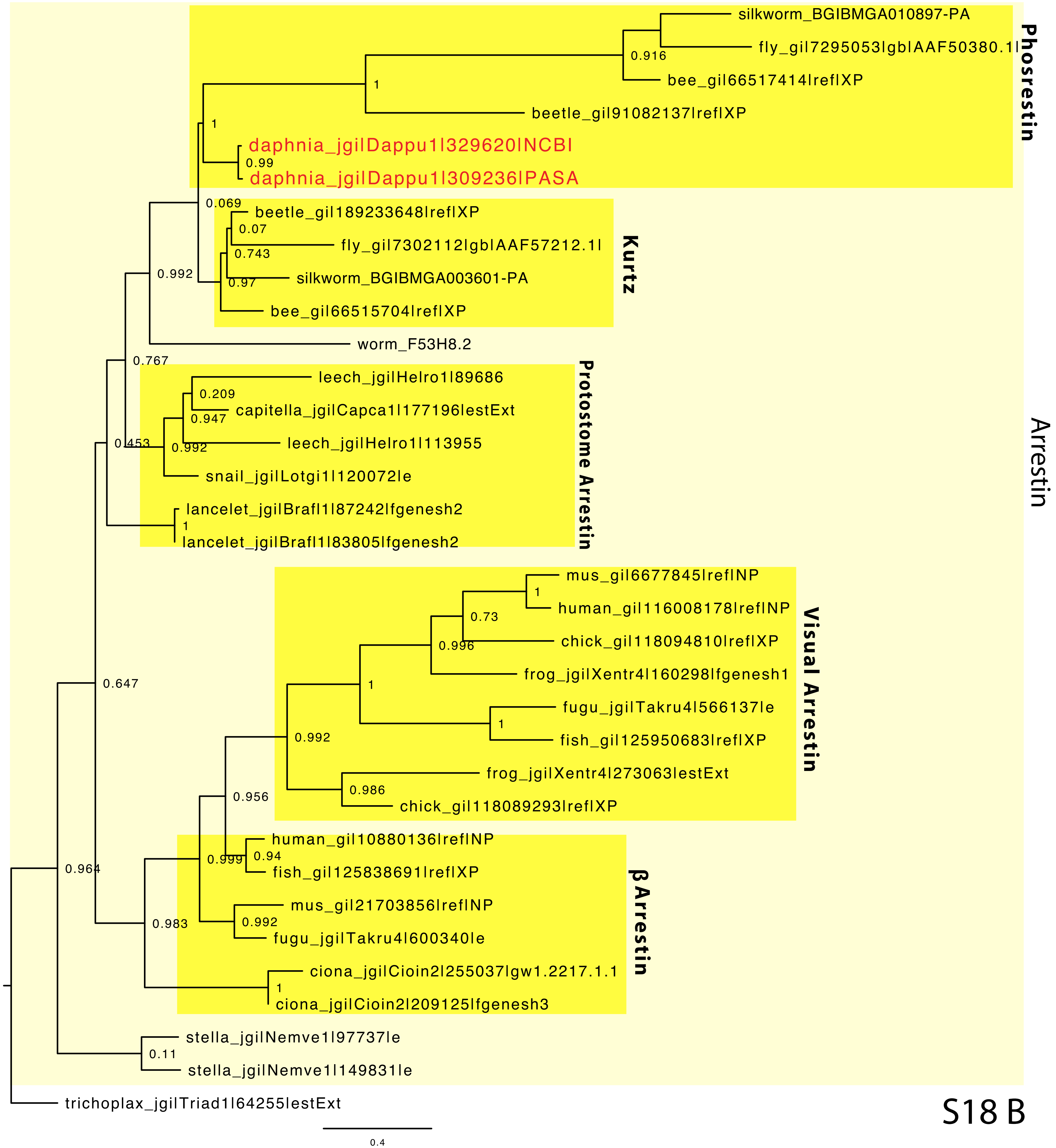

silkworm_BGIBMGA010897-PA
fly_gil7295053lgblAAF50380.1l
bee_gil66517414lreflXP
beetle_gil91082137lreflXP
daphnia_jgilDappu1l329620lNCBI
daphnia_jgilDappu1l309236lPASA
Phosrestin
beetle_gil189233648lreflXP
fly_gil7302112lgblAAF57212.1l
silkworm_BGIBMGA003601-PA
bee_gil66515704lreflXP
Kurtz
worm_F53H8.2
leech_jgilHelro1l89686
capitella_jgilCapca1l177196lestExt
leech_jgilHelro1l113955
snail_jgilLotgi1l120072le
lancelet_jgilBrafl1l87242lfgenesh2
lancelet_jgilBrafl1l83805lfgenesh2
Protostome Arrestin
mus_gil6677845lreflNP
human_gil116008178lreflNP
chick_gil118094810lreflXP
frog_jgilXentr4l160298lfgenesh1
fugu_jgilTakru4l566137le
fish_gil125950683lreflXP
frog_jgilXentr4l273063lestExt
chick_gil118089293lreflXP
Visual Arrestin
human_gil10880136lreflNP
fish_gil125838691lreflXP
mus_gil21703856lreflNP
fugu_jgilTakru4l600340le
ciona_jgilCioin2l255037lgw1.2217.1.1
ciona_jgilCioin2l209125lfgenesh3
β Arrestin
stella_jgilNemve1l97737le
stella_jgilNemve1l149831le
trichoplax_jgilTriad1l64255lestExt
Arrestin
1
0.916
1
0.99
0.069
0.07
0.743
0.97
0.992
0.767
0.209
0.947
0.453
0.992
1
1
0.73
0.996
1
1
0.992
0.986
0.956
0.999
0.94
0.992
0.647
0.964
0.983
1
0.11
0.4

S18 B

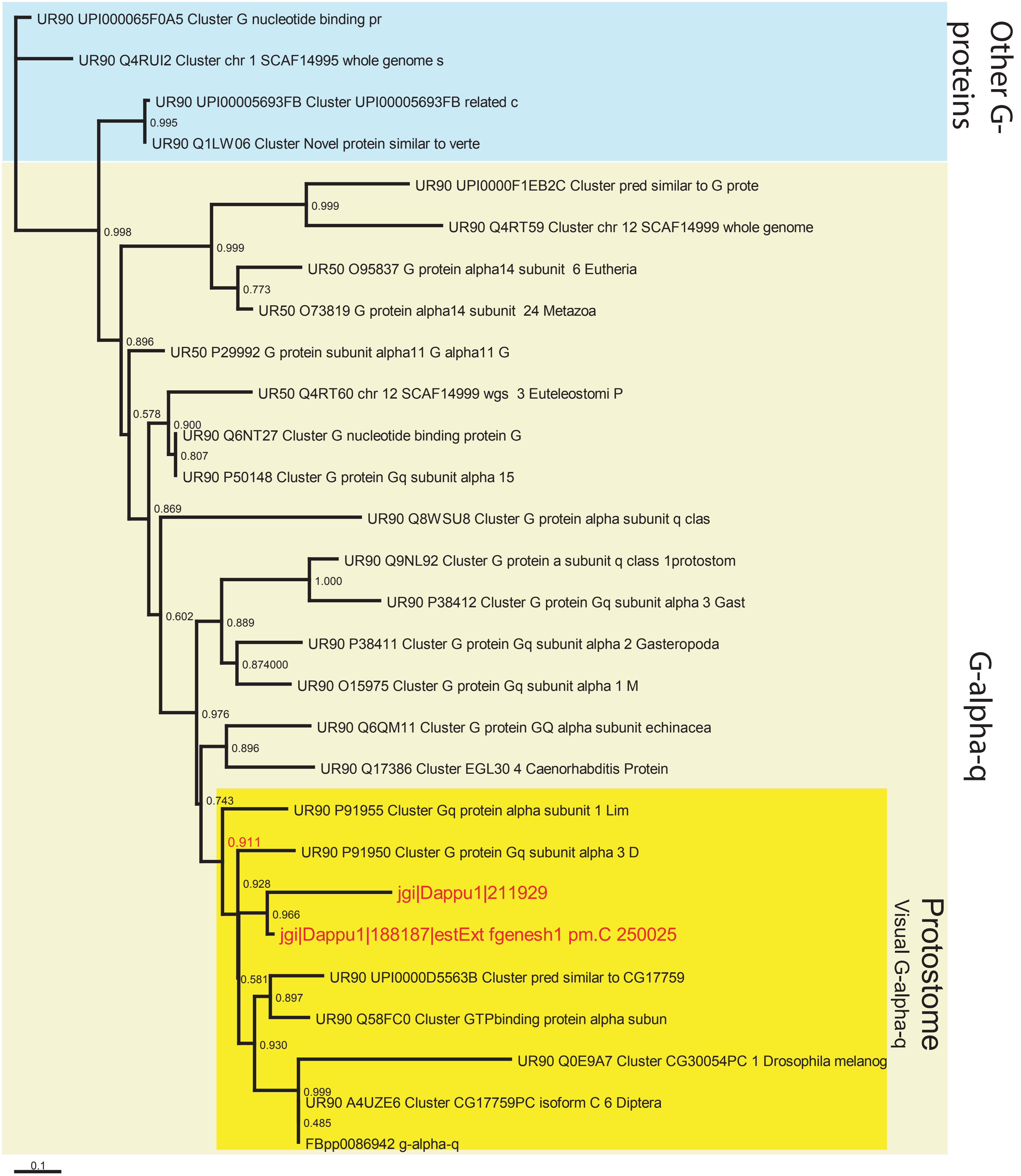
UR90 UPI000065F0A5 Cluster G nucleotide binding pr
UR90 Q4RUI2 Cluster chr 1 SCAF14995 whole genome s
UR90 UPI00005693FB Cluster UPI00005693FB related c
0.995
UR90 Q1LW06 Cluster Novel protein similar to verte
Other G-proteins
UR90 UPI0000F1EB2C Cluster pred similar to G prote
0.999
UR90 Q4RT59 Cluster chr 12 SCAF14999 whole genome
0.998
0.999
UR50 O95837 G protein alpha14 subunit 6 Eutheria
0.773
UR50 O73819 G protein alpha14 subunit 24 Metazoa
0.896
UR50 P29992 G protein subunit alpha11 G alpha11 G
UR50 Q4RT60 chr 12 SCAF14999 wgs 3 Euteleostomi P
0.578
0.900
UR90 Q6NT27 Cluster G nucleotide binding protein G
0.807
UR90 P50148 Cluster G protein Gq subunit alpha 15
0.869
UR90 Q8WSU8 Cluster G protein alpha subunit q clas
UR90 Q9NL92 Cluster G protein a subunit q class 1protostom
1.000
UR90 P38412 Cluster G protein Gq subunit alpha 3 Gast
0.602
0.889
UR90 P38411 Cluster G protein Gq subunit alpha 2 Gasteropoda
0.874000
UR90 O15975 Cluster G protein Gq subunit alpha 1 M
0.976
UR90 Q6QM11 Cluster G protein GQ alpha subunit echinacea
0.896
UR90 Q17386 Cluster EGL30 4 Caenorhabditis Protein
G-alpha-q
0.743
UR90 P91955 Cluster Gq protein alpha subunit 1 Lim
0.911
UR90 P91950 Cluster G protein Gq subunit alpha 3 D
0.928
jgi|Dappu1|211929
0.966
jgi|Dappu1|188187|estExt fgenesh1 pm.C 250025
0.581
UR90 UPI0000D5563B Cluster pred similar to CG17759
0.897
UR90 Q58FC0 Cluster GTPbinding protein alpha subun
0.930
UR90 Q0E9A7 Cluster CG30054PC 1 Drosophila melanog
0.999
UR90 A4UZE6 Cluster CG17759PC isoform C 6 Diptera
0.485
FBpp0086942 g-alpha-q
Protostome
Visual G-alpha-q
0.1

S19 A

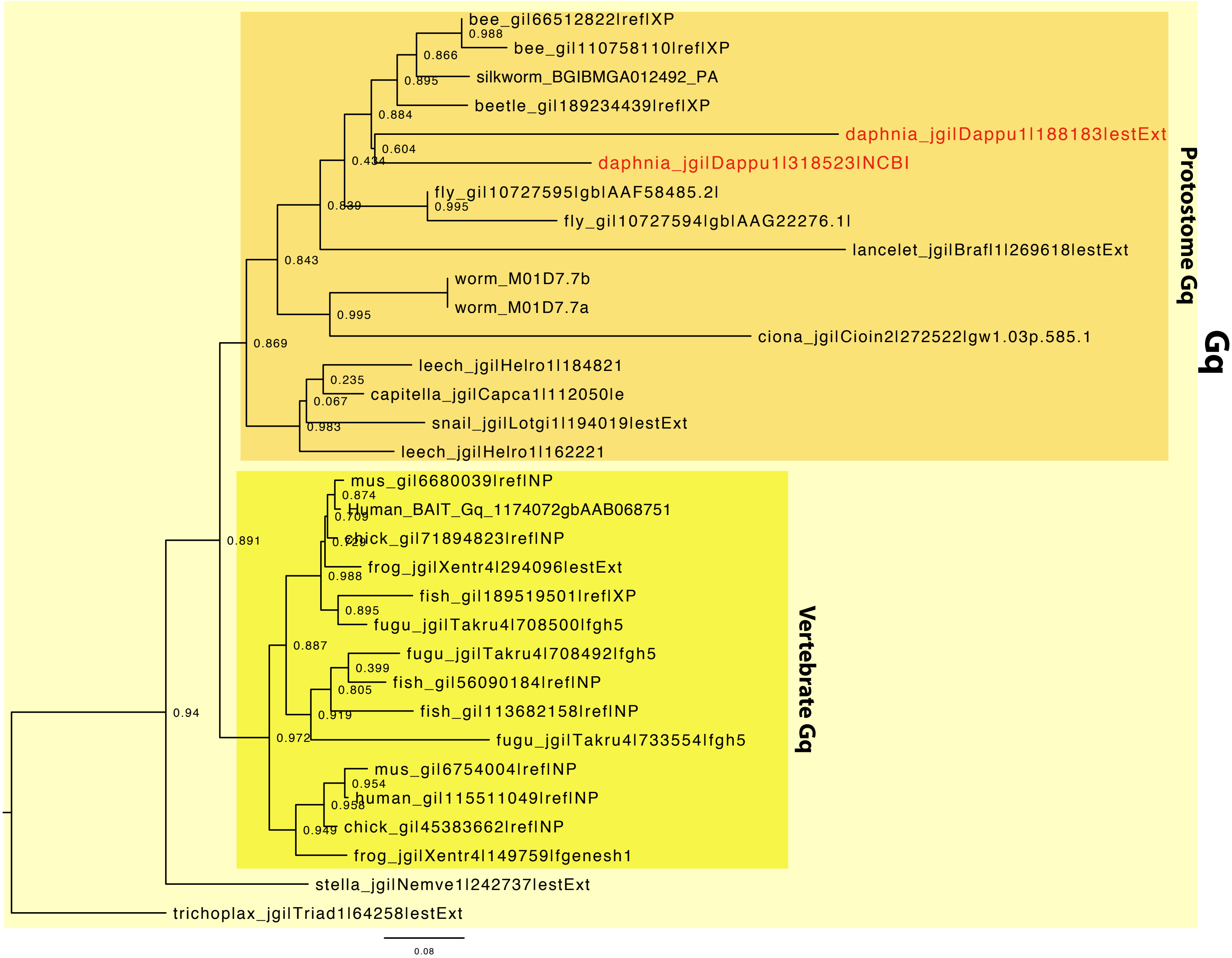

bee_gi|66512822|ref|XP
0.988
bee_gi|110758110|ref|XP
0.866
silkworm_BGIBMGA012492_PA
0.895
beetle_gi|189234439|ref|XP
0.884
daphnia_jgi|Dappu1|188183|estExt
0.604
0.434
daphnia_jgi|Dappu1|318523|NCBI
fly_gi|10727595|gb|AAF58485.2|
0.995
0.839
fly_gi|10727594|gb|AAG22276.1|
lancelet_jgi|Brafl1|269618|estExt
0.843
worm_M01D7.7b
worm_M01D7.7a
0.995
ciona_jgi|Cioin2|272522|gw1.03p.585.1
0.869
leech_jgi|Helro1|184821
0.235
capitella_jgi|Capca1|112050|e
0.067
snail_jgi|Lotgi1|194019|estExt
0.983
leech_jgi|Helro1|162221
Protostome Gq
Gq
mus_gi|6680039|ref|NP
0.874
Human_BAIT_Gq_1174072gbAAB068751
0.709
chick_gi|71894823|ref|NP
0.891
0.729
frog_jgi|Xentr4|294096|estExt
0.988
fish_gi|189519501|ref|XP
0.895
fugu_jgi|Takru4|708500|fgh5
0.887
fugu_jgi|Takru4|708492|fgh5
0.399
fish_gi|56090184|ref|NP
0.805
fish_gi|113682158|ref|NP
0.919
0.94
fugu_jgi|Takru4|733554|fgh5
0.972
mus_gi|6754004|ref|NP
0.954
human_gi|115511049|ref|NP
0.958
chick_gi|45383662|ref|NP
0.949
frog_jgi|Xentr4|149759|fgenesh1
Vertebrate Gq
stella_jgi|Nemve1|242737|estExt
trichoplax_jgi|Triad1|64258|estExt
0.08

S19 B

S20: Inferred duplication and losses events for rhabdomeric opsins (details of phylogenetic analyses are presented in Colbourne J et al: Genome Biology of the Model Crustacean Daphnia pulex, submitted).

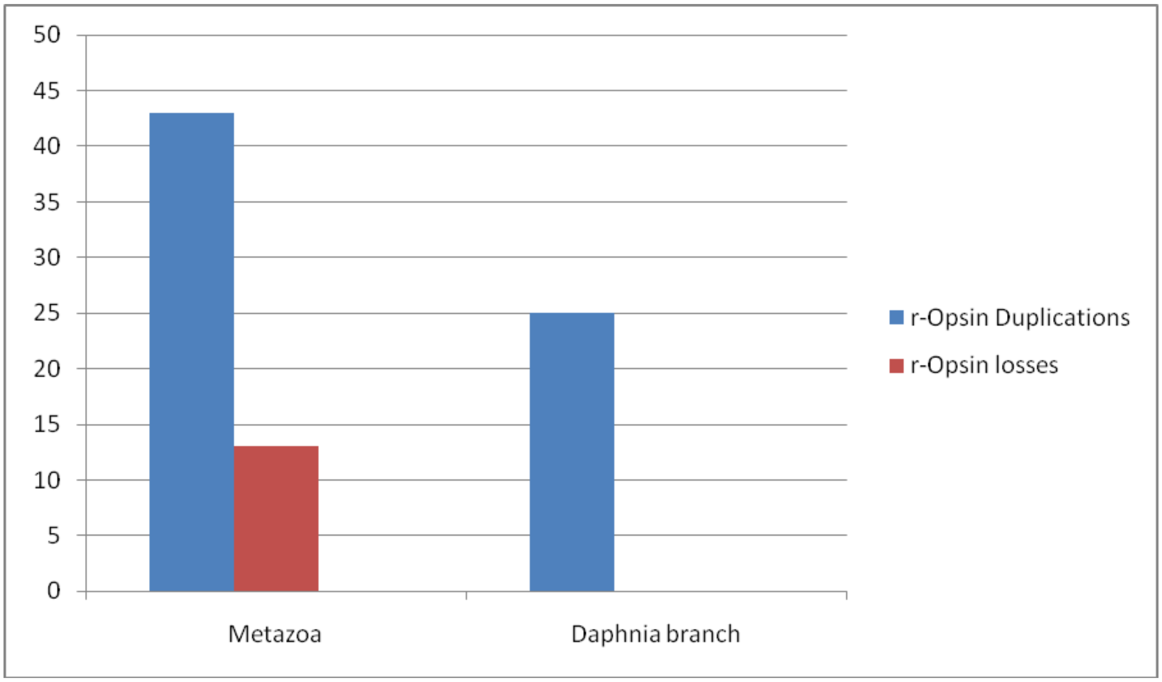

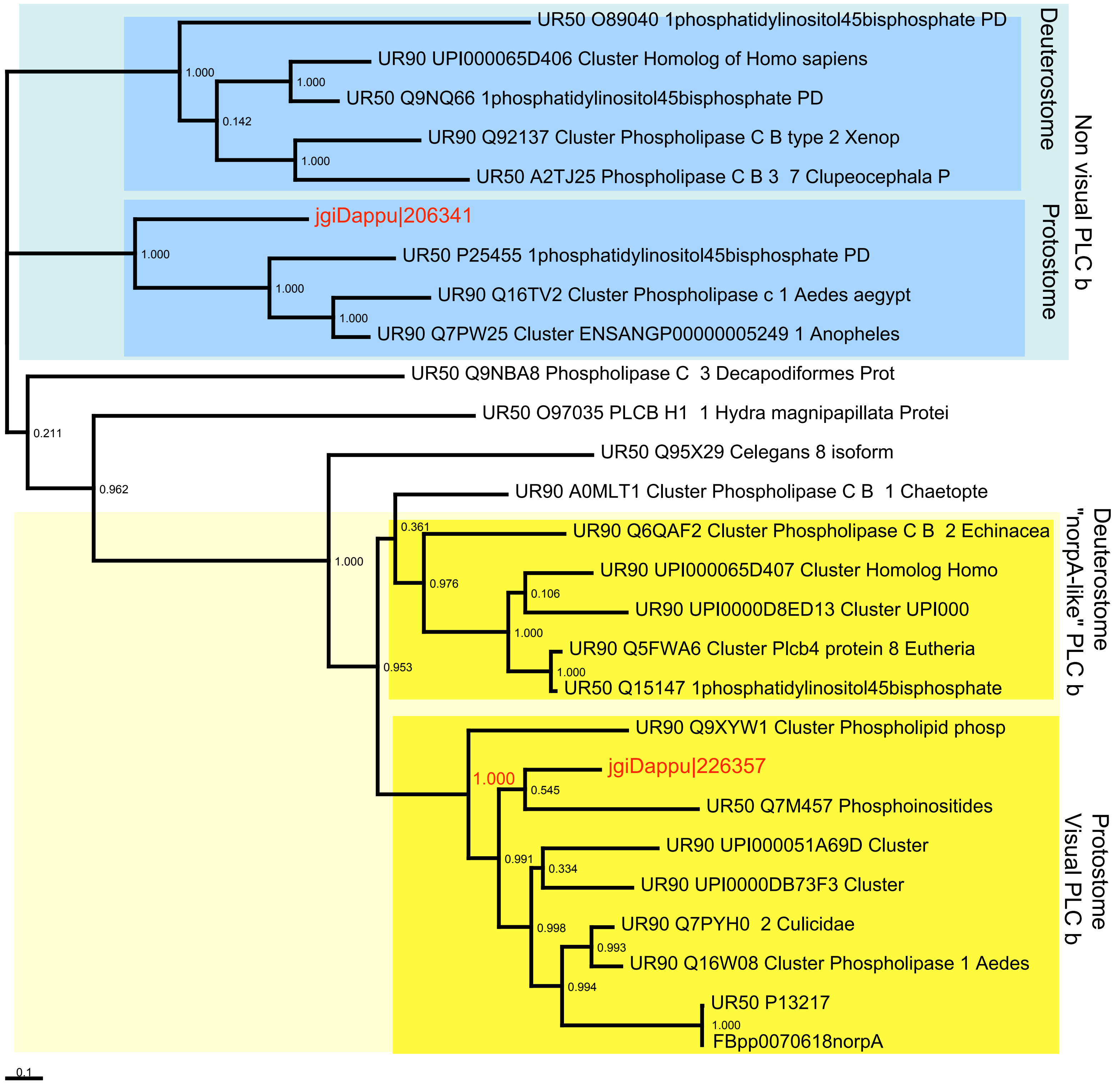

UR50 O89040 1phosphatidylinositol45bisphosphate PD
1.000
UR90 UPI000065D406 Cluster Homolog of Homo sapiens
1.000
UR50 Q9NQ66 1phosphatidylinositol45bisphosphate PD
0.142
UR90 Q92137 Cluster Phospholipase C B type 2 Xenop
1.000
UR50 A2TJ25 Phospholipase C B 3 7 Clupeocephala P
Deuterostome
jgiDappu|206341
1.000
UR50 P25455 1phosphatidylinositol45bisphosphate PD
1.000
UR90 Q16TV2 Cluster Phospholipase c 1 Aedes aegypt
1.000
UR90 Q7PW25 Cluster ENSANGP00000005249 1 Anopheles
Protostome
Non visual PLC b
UR50 Q9NBA8 Phospholipase C 3 Decapodiformes Prot
0.211
UR50 O97035 PLCB H1 1 Hydra magnipapillata Protei
0.962
UR50 Q95X29 Celegans 8 isoform
1.000
UR90 A0MLT1 Cluster Phospholipase C B 1 Chaetopte
0.361
UR90 Q6QAF2 Cluster Phospholipase C B 2 Echinacea
0.976
UR90 UPI000065D407 Cluster Homolog Homo
0.106
UR90 UPI0000D8ED13 Cluster UPI000
1.000
UR90 Q5FWA6 Cluster Plcb4 protein 8 Eutheria
1.000
UR50 Q15147 1phosphatidylinositol45bisphosphate
0.953
Deuterostome
"norpA-like" PLC b
UR90 Q9XYW1 Cluster Phospholipid phosp
1.000
jgiDappu|226357
0.545
UR50 Q7M457 Phosphoinositides
UR90 UPI000051A69D Cluster
0.991
0.334
UR90 UPI0000DB73F3 Cluster
0.998
UR90 Q7PYH0 2 Culicidae
0.993
UR90 Q16W08 Cluster Phospholipase 1 Aedes
0.994
UR50 P13217
1.000
FBpp0070618norpA
Protostome
Visual PLC b
0.1

S21 A

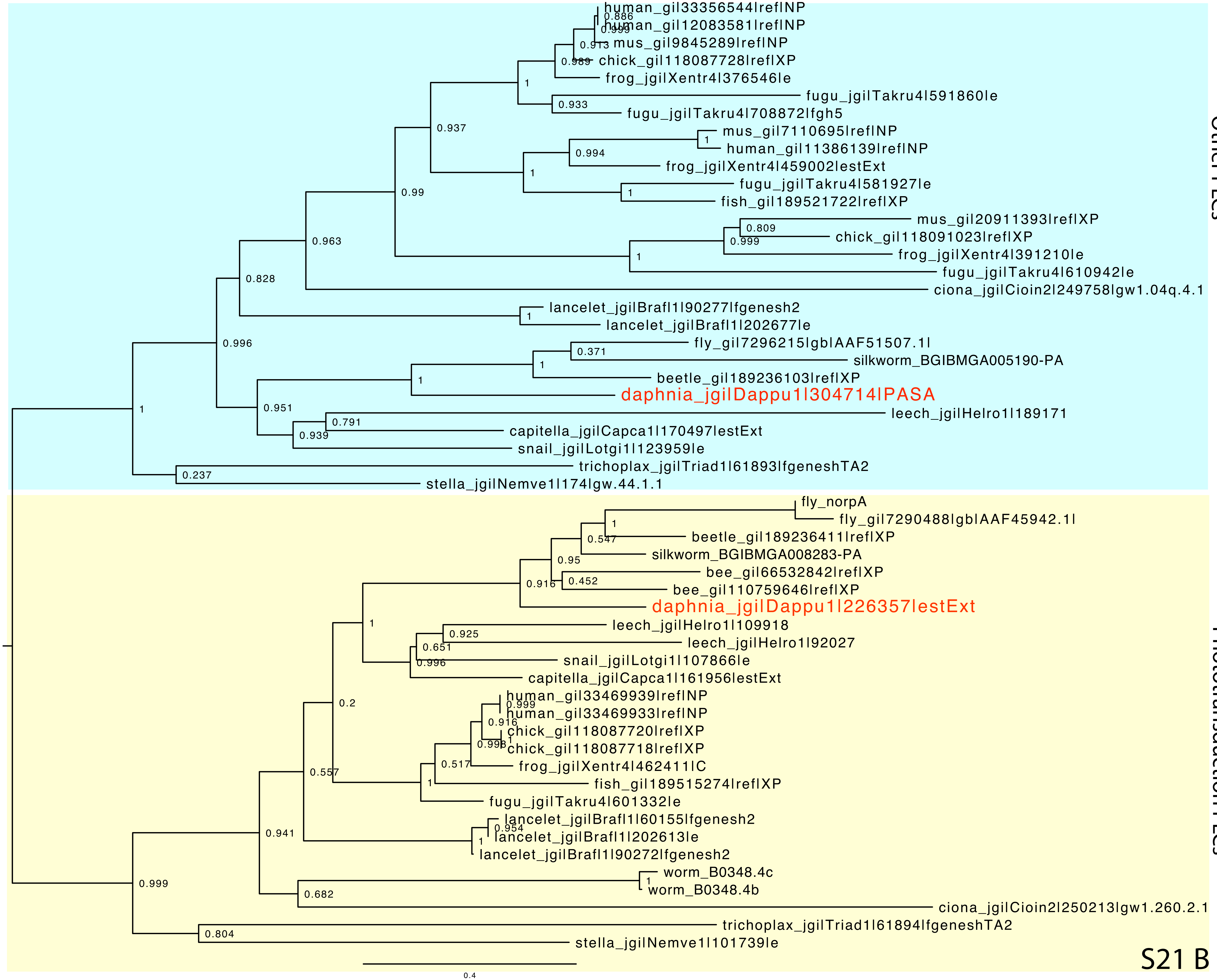
Other PLCs
human_gi|33356544|ref|NP
human_gi|12083581|ref|NP
mus_gi|9845289|ref|NP
chick_gi|118087728|ref|XP
frog_jgi|Xentr4|376546|e
fugu_jgi|Takru4|591860|e
fugu_jgi|Takru4|708872|fgh5
mus_gi|7110695|ref|NP
human_gi|11386139|ref|NP
frog_jgi|Xentr4|459002|estExt
fugu_jgi|Takru4|581927|e
fish_gi|189521722|ref|XP
mus_gi|20911393|ref|XP
chick_gi|118091023|ref|XP
frog_jgi|Xentr4|391210|e
fugu_jgi|Takru4|610942|e
ciona_jgi|Cioin2|249758|gw1.04q.4.1
lancelet_jgi|Brafl1|90277|fgenesh2
lancelet_jgi|Brafl1|202677|e
fly_gi|7296215|gb|AAF51507.1|
silkworm_BGIBMGA005190-PA
beetle_gi|189236103|ref|XP
daphnia_jgi|Dappu1|304714|PASA
leech_jgi|Helro1|189171
capitella_jgi|Capca1|170497|estExt
snail_jgi|Lotgi1|123959|e
trichoplax_jgi|Triad1|61893|fgeneshTA2
stella_jgi|Nemve1|174|gw.44.1.1
Phototransduction PLCs
fly_norpA
fly_gi|7290488|gb|AAF45942.1|
beetle_gi|189236411|ref|XP
silkworm_BGIBMGA008283-PA
bee_gi|66532842|ref|XP
bee_gi|110759646|ref|XP
daphnia_jgi|Dappu1|226357|estExt
leech_jgi|Helro1|109918
leech_jgi|Helro1|92027
snail_jgi|Lotgi1|107866|e
capitella_jgi|Capca1|161956|estExt
human_gi|33469939|ref|NP
human_gi|33469933|ref|NP
chick_gi|118087720|ref|XP
chick_gi|118087718|ref|XP
frog_jgi|Xentr4|462411|C
fish_gi|189515274|ref|XP
fugu_jgi|Takru4|601332|e
lancelet_jgi|Brafl1|60155|fgenesh2
lancelet_jgi|Brafl1|202613|e
lancelet_jgi|Brafl1|90272|fgenesh2
worm_B0348.4c
worm_B0348.4b
ciona_jgi|Cioin2|250213|gw1.260.2.1
trichoplax_jgi|Triad1|61894|fgeneshTA2
stella_jgi|Nemve1|101739|e
0.4
S21 B

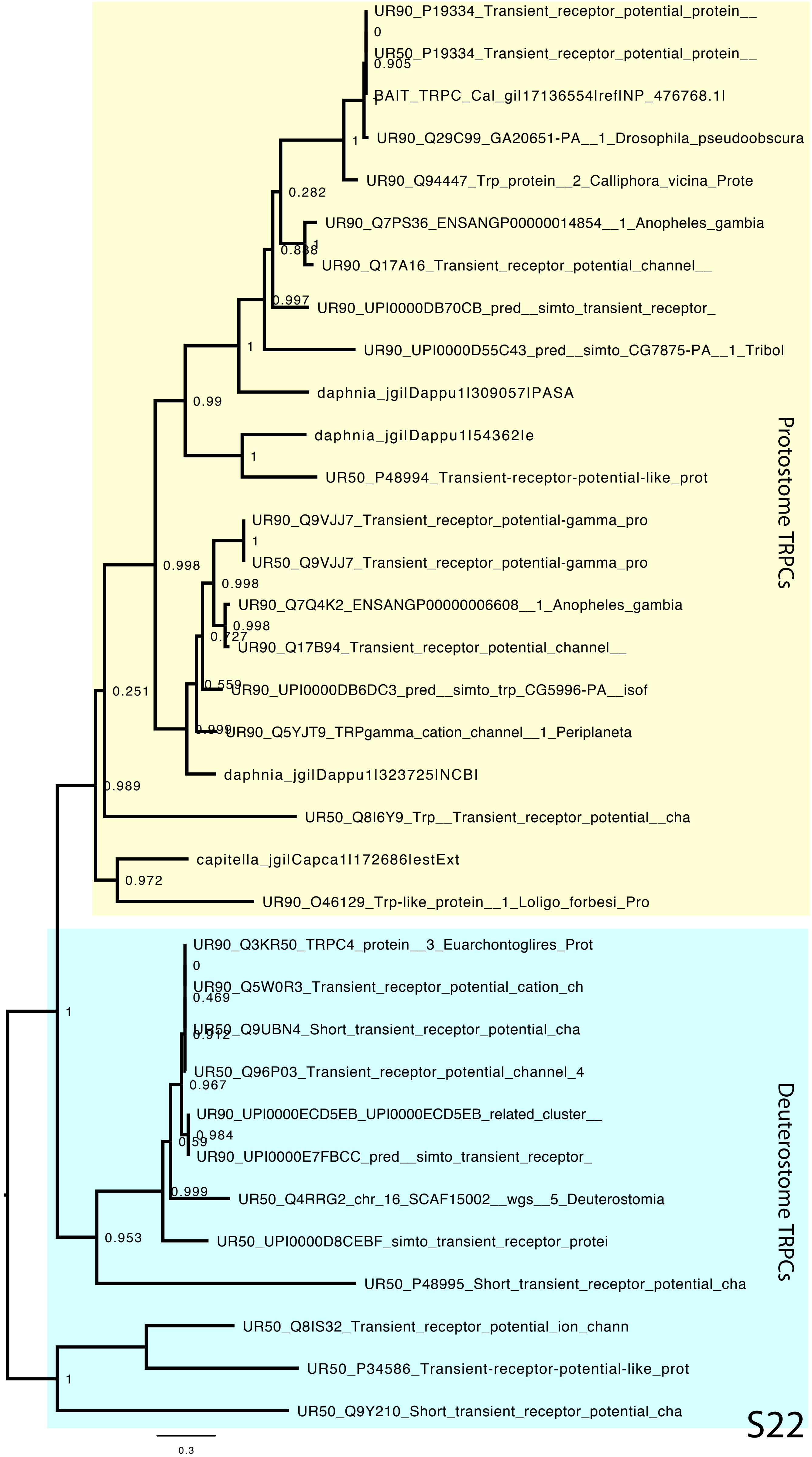
UR90_P19334_Transient_receptor_potential_protein__
0
UR50_P19334_Transient_receptor_potential_protein__
0.905
BAIT_TRPC_Cal_gi|17136554|ref|NP_476768.1|
1
UR90_Q29C99_GA20651-PA__1_Drosophila_pseudoobscura
1
UR90_Q94447_Trp_protein__2_Calliphora_vicina_Prote
0.282
UR90_Q7PS36_ENSANGP00000014854__1_Anopheles_gambia
1
0.888
UR90_Q17A16_Transient_receptor_potential_channel__
0.997
UR90_UPI0000DB70CB_pred__simto_transient_receptor_
1
UR90_UPI0000D55C43_pred__simto_CG7875-PA__1_Tribol
daphnia_jgi|Dappu1|309057|PASA
0.99
daphnia_jgi|Dappu1|54362|e
1
UR50_P48994_Transient-receptor-potential-like_prot
UR90_Q9VJJ7_Transient_receptor_potential-gamma_pro
1
UR50_Q9VJJ7_Transient_receptor_potential-gamma_pro
0.998
0.998
UR90_Q7Q4K2_ENSANGP00000006608__1_Anopheles_gambia
0.998
0.727
UR90_Q17B94_Transient_receptor_potential_channel__
0.251
0.559
UR90_UPI0000DB6DC3_pred__simto_trp_CG5996-PA__isof
0.999
UR90_Q5YJT9_TRPgamma_cation_channel__1_Periplaneta
daphnia_jgi|Dappu1|323725|NCBI
0.989
UR50_Q8I6Y9_Trp__Transient_receptor_potential__cha
capitella_jgi|Capca1|172686|estExt
0.972
UR90_O46129_Trp-like_protein__1_Loligo_forbesi_Pro
Protostome TRPCs
UR90_Q3KR50_TRPC4_protein__3_Euarchontoglires_Prot
0
UR90_Q5W0R3_Transient_receptor_potential_cation_ch
0.469
1
UR50_Q9UBN4_Short_transient_receptor_potential_cha
0.912
UR50_Q96P03_Transient_receptor_potential_channel_4
0.967
UR90_UPI0000ECD5EB_UPI0000ECD5EB_related_cluster__
0.984
0.59
UR90_UPI0000E7FBCC_pred__simto_transient_receptor_
0.999
UR50_Q4RRG2_chr_16_SCAF15002__wgs__5_Deuterostomia
0.953
UR50_UPI0000D8CEBF_simto_transient_receptor_protei
UR50_P48995_Short_transient_receptor_potential_cha
UR50_Q8IS32_Transient_receptor_potential_ion_chann
1
UR50_P34586_Transient-receptor-potential-like_prot
UR50_Q9Y210_Short_transient_receptor_potential_cha
Deuterostome TRPCs
0.3

S22 A

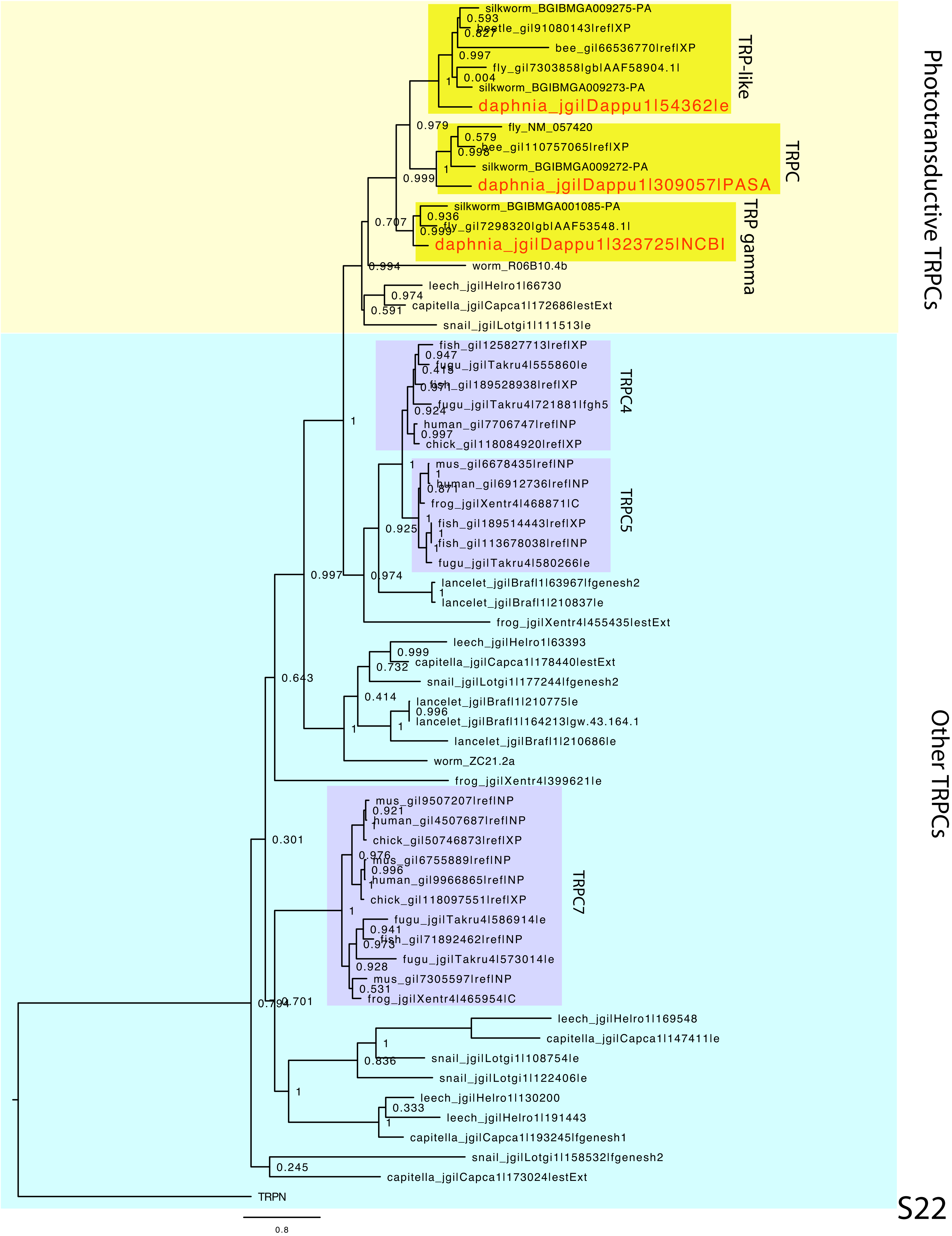

Phototransductive TRPCs
Other TRPCs
TRP-like
TRPC
TRP gamma
TRPC4
TRPC5
TRPC7
silkworm_BGIBMGA009275-PA
beetle_gil91080143lreflXP
bee_gil66536770lreflXP
fly_gil7303858lgblAAF58904.1l
silkworm_BGIBMGA009273-PA
daphnia_jgilDappu1l54362le
fly_NM_057420
bee_gil110757065lreflXP
silkworm_BGIBMGA009272-PA
daphnia_jgilDappu1l309057lPASA
silkworm_BGIBMGA001085-PA
fly_gil7298320lgblAAF53548.1l
daphnia_jgilDappu1l323725lNCBI
worm_R06B10.4b
leech_jgilHelro1l66730
capitella_jgilCapca1l172686lestExt
snail_jgilLotgi1l111513le
fish_gil125827713lreflXP
fugu_jgilTakru4l555860le
fish_gil189528938lreflXP
fugu_jgilTakru4l721881lfgh5
human_gil7706747lreflNP
chick_gil118084920lreflXP
mus_gil6678435lreflNP
human_gil6912736lreflNP
frog_jgilXentr4l468871lC
fish_gil189514443lreflXP
fish_gil113678038lreflNP
fugu_jgilTakru4l580266le
lancelet_jgilBrafl1l63967lfgenesh2
lancelet_jgilBrafl1l210837le
frog_jgilXentr4l455435lestExt
leech_jgilHelro1l63393
capitella_jgilCapca1l178440lestExt
snail_jgilLotgi1l177244lfgenesh2
lancelet_jgilBrafl1l210775le
lancelet_jgilBrafl1l164213lgw.43.164.1
lancelet_jgilBrafl1l210686le
worm_ZC21.2a
frog_jgilXentr4l399621le
mus_gil9507207lreflNP
human_gil4507687lreflNP
chick_gil50746873lreflXP
mus_gil6755889lreflNP
human_gil9966865lreflNP
chick_gil118097551lreflXP
fugu_jgilTakru4l586914le
fish_gil71892462lreflNP
fugu_jgilTakru4l573014le
mus_gil7305597lreflNP
frog_jgilXentr4l465954lC
leech_jgilHelro1l169548
capitella_jgilCapca1l147411le
snail_jgilLotgi1l108754le
snail_jgilLotgi1l122406le
leech_jgilHelro1l130200
leech_jgilHelro1l191443
capitella_jgilCapca1l193245lfgenesh1
snail_jgilLotgi1l158532lfgenesh2
capitella_jgilCapca1l173024lestExt
TRPN
0.8

S22 B

References

1. Royet J, Finkelstein R: **Establishing primordia in the Drosophila eye-antennal imaginal disc: the roles of decapentaplegic, wingless and hedgehog**. *Development* 1997, **124**(23):4793-4800.
2. Matus DQ, Thomsen GH, Martindale MQ: **Dorso/ventral genes are asymmetrically expressed and involved in germ-layer demarcation during Cnidarian gastrulation**. *Current Biology* 2006, **16**(5):499-505.
3. Technau U, Rudd S, Maxwell P, Gordon PMK, Saina M, Grasso LC, Hayward DC, Sensen CW, Saint R, Holstein TW *et al*: **Maintenance of ancestral complexity and non-metazoan genes in two basal cnidarians**. *Trends in Genetics* 2005, **21**(12):633-639.
4. Shimodaira H, Hasegawa M: **CONSEL: for assessing the confidence of phylogenetic tree selection**. *Bioinformatics* 2001, **17**(12):1246-1247.
5. Brower DL: **Engrailed gene expression in Drosophila imaginal discs**. *The EMBO journal* 1986, **5**(10):2649-2656.
6. Amin A: **Genetic Cross-Talk During Head Development in *Drosophila***. *Journal of biomedicine & biotechnology* 2004, **2004**(1):16-23.
7. Force A, Lynch M, Pickett FB, Amores A, Yan YL, Postlethwait J: **Preservation of duplicate genes by complementary, degenerative mutations**. *Genetics* 1999, **151**(4):1531-1545.
8. Matus DQ, Magie CR, Pang K, Martindale MQ, Thomsen GH: **The Hedgehog gene family of the cnidarian, Nematostella vectensis, and implications for understanding metazoan Hedgehog pathway evolution**. *Dev Biol* 2008, **313**(2):501-518.
9. Duman-Scheel M, Pirkl N, Patel NH: **Analysis of the expression pattern of Mysidium columbiae wingless provides evidence for conserved mesodermal and retinal patterning processes among insects and crustaceans**. *Dev Genes Evol* 2002, **212**(3):114-123.
10. Hurley I, Fowler K, Pomiankowski A, Smith H: **Conservation of the expression of Dll, en, and wg in the eye-antennal imaginal disc of stalk-eyed flies**. *Evol Dev* 2001, **3**(6):408-414.
11. Friedrich M, Benzer S: **Divergent *decapentaplegic* expression patterns in compound eye development and the evolution of insect metamorphosis**. *J Exp Zool* 2000, **288**(1):39-55.
12. Liu ZY, Yang XY, Dong Y, Friedrich M: **Tracking down the "head blob": Comparative analysis of wingless expression in the developing insect procephalon reveals progressive reduction of embryonic visual system patterning in higher insects**. 2006, **35**(4):341-356.
13. Schubert M, Holland LZ, Holland ND, Jacobs DK: **A phylogenetic tree of the Wnt genes based on all available full-length sequences, including five from the cephalochordate amphioxus**. *Mol Biol Evol* 2000, **17**(12):1896-1903.
14. Prud'homme B, Lartillot N, Balavoine G, Adoutte A, Vervoort M: **Phylogenetic analysis of the Wnt gene family: Insights from lophotrochozoan members**. *Current Biology* 2002, **12**(16):1395-1400.

15. Kusserow A, Pang K, Sturm C, Hrouda M, Lentfer J, Schmidt HA, Technau U, von Haeseler A, Hobmayer B, Martindale MQ *et al*: **Unexpected complexity of the Wnt gene family in a sea anemone**. *Nature* 2005, **433**(7022):156-160.
16. Chang T, Mazotta J, Dumstrei K, Dumitrescu A, Hartenstein V: **Dpp and Hh signaling in the Drosophila embryonic eye field**. *Development* 2001, **128**(23):4691-4704.
17. Anderson J, Salzer CL, Kumar JP: **Regulation of the retinal determination gene dachshund in the embryonic head and developing eye of Drosophila**. *Dev Biol* 2006, **297**(2):536-549.
18. Brown NL, Patel S, Brzezinski J, Glaser T: **Math5 is required for retinal ganglion cell and optic nerve formation**. *Development* 2001, **128**(13):2497-2508.
19. Panfilio KA, Liu PZ, Akam M, Kaufman TC: **Oncopeltus fasciatus zen is essential for serosal tissue function in katatrepsis**. *Dev Biol* 2006, **292**(1):226-243.
20. Tavsanli BC, Ostrin EJ, Burgess HK, Middlebrooks BW, Pham TA, Mardon G: **Structure-function analysis of the Drosophila retinal determination protein Dachshund**. *Dev Biol* 2004, **272**(1):231-247.
21. Davis GK, Patel NH: **The origin and evolution of segmentation**. *Trends Cell Biol* 1999, **9**(12):M68-M72.
22. Silver SJ, Rebay L: **Signaling circuitries in development: insights from the retinal determination gene network**. *Development* 2005, **132**(1):3-13.
23. Kumar JP: **The molecular circuitry governing retinal determination**. *Biochimica et biophysica acta* 2009, **1789**(4):306-314.
24. Mardon G, Solomon NM, Rubin GM: **Dachshund Encodes a Nuclear-Protein Required for Normal Eye and Leg Development in Drosophila**. *Development* 1994, **120**(12):3473-3486.
25. Angelini DR, Kaufman TC: **Functional analyses in the hemipteran Oncopeltus fasciatus reveal conserved and derived aspects of appendage patterning in insects**. *Dev Biol* 2004, **271**(2):306-321.
26. Inoue Y, Miyawaki K, Terasawa T, Matsushima K, Shinmyo Y, Niwa N, Mito T, Ohuchi H, Noji S: **Expression patterns of dachshund during head development of Gryllus bimaculatus (cricket)**. *Gene Expr Patterns* 2004, **4**(6):725-731.
27. Friedrich M: **Ancient mechanisms of visual sense organ development based on comparison of the gene networks controlling larval eye, ocellus, and compound eye specification in *Drosophila***. *Arthropod Structure and Development* 2006, **35**(4):357-378.
28. Mazet F, Hutt JA, Milloz J, Millard J, Graham A, Shimeld SM: **Molecular evidence from Ciona intestinalis for the evolutionary origin of vertebrate sensory placodes**. *Dev Biol* 2005, **282**(2):494-508.
29. Dominguez M, Ferres-Marco D, Gutierrez-Avino FJ, Speicher SA, Beneyto M: **Growth and specification of the eye are controlled independently by Eyegone and Eyeless in Drosophila melanogaster**. *Nat Genet* 2004, **36**(1):31-39.
30. Grinchuk O, Kozmik Z, Wu X, Tomarev S: **The Optimedin gene is a downstream target of Pax6**. *J Biol Chem* 2005, **280**(42):35228-35237.

31. Kumar JP, Moses K: **EGF receptor and Notch signaling act upstream of Eyeless/Pax6 to control eye specification**. *Cell* 2001, **104**(5):687-697.
32. Quiring R, Walldorf U, Kloter U, Gehring WJ: **Homology of the *eyeless* gene of *Drosophila* to the Small eye gene in mice and Aniridia in humans**. *Science* 1994, **265**(5173):785-789.
33. Aruga J, Odaka YS, Kamiya A, Furuya H: **Dicyema Pax6 and Zic: tool-kit genes in a highly simplified bilaterian**. *Bmc Evolutionary Biology* 2007, **7**:-.
34. Hoshiyama D, Iwabe N, Miyata T: **Evolution of the gene families forming the Pax/Six regulatory network: Isolation of genes from primitive animals and molecular phylogenetic analyses**. *Febs Lett* 2007, **581**(8):1639-1643.
35. Prpic N: **Duplicated Pax-6 genes in Glomeris marginata (Myriapoda:Diplopoda), an arthropod with simple lateral eyes**. *Zoology* 2005, **108**:47-53.
36. Conley KW, Blackburn DC, Plachetzki D, Kempler K, Currie J, Battelle BA, Brown L: **Pax6 and Atonal expression during embryonic eye formation in the American Horseshoe crab, Limulus polyphemus**. *Dev Biol* 2006, **295**(1):417-417.
37. Pineda D, Gonzalez J, Callaerts P, Ikeo K, Gehring WJ, Salo E: **Searching for the prototypic eye genetic network: Sine oculis is essential for eye regeneration in planarians**. *Proc Natl Acad Sci U S A* 2000, **97**(9):4525-4529.
38. Hsiao FC, Williams A, Davies EL, Rebay I: **Eyes absent mediates cross-talk between retinal determination genes and the receptor tyrosine kinase signaling pathway**. *Dev Cell* 2001, **1**(1):51-61.
39. Amin A, Finkelstein R: **Epidermal growth factor receptor signaling activates orthodenticle expression during Drosophila head development**. *DNA and cell biology* 2000, **19**(11):631-638.
40. Stein RA, Staros JV: **Insights into the evolution of the ErbB receptor family and their ligands from sequence analysis**. *Bmc Evolutionary Biology* 2006, **6**:-.
41. Liu Z: **The photoreceptor differentiation factor *glass* in the flour beetle *Tribolium castaneum*: cloning, function and evolution**. Detroit, MI: Wayne State; 2005.
42. Liu Z, Friedrich M: **The Tribolium homologue of glass and the evolution of insect larval eyes**. *Dev Biol* 2004, **269**(1):36-54.
43. Moses K, Rubin GM: **Glass Encodes a Site-Specific DNA-Binding Protein That Is Regulated in Response to Positional Signals in the Developing Drosophila Eye**. *Genes & Development* 1991, **5**(4):583-593.
44. Ellis MC, O'Neill EM, Rubin GM: **Expression of Drosophila glass protein and evidence for negative regulation of its activity in non-neuronal cells by another DNA-binding protein**. *Development* 1993, **119**(3):855-865.
45. Goriely A, Mollereau B, Coffinier C, Desplan C: **Munster, a novel Paired-class homeobox gene specifically expressed in the Drosophila larval eye**. *Mechanisms of Development* 1999, **88**(1):107-110.
46. Srivastava M, Begovic E, Chapman J, Putnam NH, Hellsten U, Kawashima T, Kuo A, Mitros T, Salamov A, Carpenter ML *et al*: **The Trichoplax genome and the nature of placozoans**. *Nature* 2008, **454**(7207):955-U919.

47. King N, Westbrook MJ, Young SL, Kuo A, Abedin M, Chapman J, Fairclough S, Hellsten U, Isogai Y, Letunic I *et al*: **The genome of the choanoflagellate Monosiga brevicollis and the origin of metazoans**. *Nature* 2008, **451**(7180):783-788.
48. Onuma Y, Takahashi S, Asashima M, Kurata S, Gehring WJ: **Conservation of *Pax-6* function and upstream activation by *Notch* signaling in eye development of frogs and flies**. *Proceedings of the National Academy of Sciences USA* 2002, **99**(4):2020-2025.
49. Erclik T, Hartenstein V, Lipshitz HD, McInnes RR: **Conserved role of the Vsx genes supports a monophyletic origin for bilaterian visual systems**. *Current Biology* 2008, **18**(17):1278-1287.
50. Chow RL, Volgyi B, Szilard RK, Ng D, McKerlie C, Bloomfield SA, Birch DG, McInnes RR: **Control of late off-center cone bipolar cell differentiation and visual signaling by the homeobox gene Vsx1**. *P Natl Acad Sci USA* 2004, **101**(6):1754-1759.
51. Hardie RC, Raghu P: **Visual transduction in Drosophila**. *Nature* 2001, **413**(6852):186-193.
52. Matsumoto H, Kurien BT, Takagi Y, Kahn ES, Kinumi T, Kamori N, Yamada T, Hayashi F, Isono K, Pak WL *et al*: **Phosrestin-I Undergoes the Earliest Light-Induced Phosphorylation by a Calcium/Calmodulin-Dependent Protein-Kinase in Drosophila Photoreceptors**. *Neuron* 1994, **12**(5):997-1010.
53. Lefkowitz RJ, Shenoy SK: **Transduction of receptor signals by beta-arrestins**. *Science* 2005, **308**(5721):512-517.
54. Tsuda M, Kusakabe T, Iwamoto H, Horie T, Nakashima Y, Nakagawa M, Okunou K: **Origin of the vertebrate visual cycle: II. Visual cycle proteins are localized in whole brain including photoreceptor cells of a primitive chordate**. *Vision research* 2003, **43**(28):3045-3053.
55. Nakagawa M, Orii H, Yoshida N, Jojima E, Horie T, Yoshida R, Haga T, Tsuda M: **Ascidian arrestin (Ci-arr), the origin of the visual and nonvisual arrestins of vertebrate**. *European Journal of Biochemistry* 2002, **269**(21):5112-5118.
56. Suga H, Koyanagi M, Hoshiyama D, Ono K, Iwabe N, Kuma K, Miyata T: **Extensive gene duplication in the early evolution of animals before the parazoan-eumetazoan split demonstrated by G proteins and protein tyrosine kinases from sponge and hydra**. *Journal of Molecular Evolution* 1999, **48**(6):646-653.
57. Plachetzki DC, Degnan BM, Oakley TH: **The Origins of Novel Protein Interactions during Animal Opsin Evolution**. *PLoS ONE* 2007, **2**(10):e1054.
58. Venkatachalam K, Montell C: **TRP channels**. *Annual Review of Biochemistry* 2007, **76**:387-417.
59. Hartwick ATE, Bramley JR, Yu J, Stevens KT, Allen CN, Baldridge WH, Sollars PJ, Pickard GE: **Light-evoked calcium responses of isolated melanopsin-expressing retinal ganglion cells**. *J Neurosci* 2007, **27**(49):13468-13480.
60. Gillo B, Chorna I, Cohen H, Cook B, Manistersky I, Chorev M, Arnon A, Pollock JA, Selinger Z, Minke B: **Coexpression of Drosophila TRP and TRP-like proteins in Xenopus oocytes reconstitutes capacitative Ca2+ entry**. *P Natl Acad Sci USA* 1996, **93**(24):14146-14151.

61. Meyer NE, Joel-Almagor T, Frechter S, Minke B, Huber A: **Subcellular translocation of the eGFP-tagged TRPL channel in Drosophila photoreceptors requires activation of the phototransduction cascade**. *Journal of Cell Science* 2006, **119**(12):2592-2603.
