# Supplementary material for: Gene duplication and the origins of morphological complexity in pancrustacean eyes, a genomic approach
Source: BMC Evol Biol. 2010 Apr 30;10:123. doi: 10.1186/1471-2148-10-123 (PMC2888819; doi:10.1186/1471-2148-10-123)
Supplement: Additional file 2 — Pairwise correlation values of between duplication and loss of the 22 gene families examined. [file 1471-2148-10-123-S2.PDF]

Co-duplication correlation values (* denotes significance)

	dpp	en	hh	wnt1	zen	dac	eya	eygtoe	pax6	six	egfr	glass	kr	notch	spam	spitz	vsx	arr	gq	opsin	plc
**en**	0.42																				
**hh**	**0.54***	0.29																			
**wnt1**	0.00	0.00	0.00																		
**zen**	-0.08	-0.09	-0.10	0.00																	
**dac**	0.26	0.25	0.46	0.00	-0.06																
**eya**	0.21	0.03	**0.54***	0.00	-0.07	0.39															
**eygtoe**	0.33	0.29	-0.10	0.00	-0.03	-0.06	-0.07														
**pax6**	0.00	0.38	-0.06	0.00	-0.07	0.13	-0.16	-0.07													
**six**	0.05	-0.19	0.39	0.00	-0.06	0.15	0.35	-0.06	0.13												
**egfr**	-0.01	0.20	0.16	0.00	-0.08	0.34	0.30	-0.08	0.45	0.31											
**glass**	0.04	0.01	-0.02	0.00	-0.06	-0.12	0.11	-0.06	0.11	0.16	-0.16										
**kr**	-0.08	-0.09	-0.10	0.00	-0.03	-0.06	-0.07	-0.03	0.42	-0.06	0.36	-0.06									
**notch**	0.35	**0.63***	0.32	0.00	-0.05	**0.54***	0.22	-0.05	0.44	-0.11	0.44	-0.11	-0.05								
**spam**	-0.08	-0.09	-0.10	0.00	1.00	-0.06	-0.07	-0.03	-0.07	-0.06	-0.08	-0.06	-0.03	-0.05							
**spitz**	-0.12	-0.13	-0.14	0.00	**0.72***	-0.09	-0.10	-0.04	-0.10	-0.09	-0.11	-0.09	-0.04	-0.07	**0.72***						
**vsx**	0.20	0.19	-0.02	0.00	-0.07	0.10	0.10	-0.07	0.07	-0.14	0.07	0.11	-0.07	0.19	-0.07	-0.10					
**arr**	0.18	-0.13	0.12	0.00	-0.04	-0.09	0.24	-0.04	-0.10	0.31	-0.11	0.30	-0.04	-0.07	-0.04	0.46	-0.10				
**gq**	0.26	0.25	0.22	0.00	-0.06	0.44	0.14	-0.06	**0.62***	0.18	**0.57***	-0.13	0.48	**0.54***	-0.06	-0.09	0.11	-0.09			
**opsin**	0.10	0.43	0.19	0.00	0.24	0.36	0.02	-0.13	0.35	0.10	**0.55***	-0.27	0.28	0.32	0.24	0.09	0.00	-0.19	**0.50***		
**plc**	0.24	0.20	0.15	0.00	0.24	0.00	-0.02	-0.10	0.08	-0.02	0.24	-0.22	-0.10	0.29	0.24	0.10	-0.04	-0.15	0.19	0.24	
**trp**	-0.15	0.36	0.03	0.00	-0.05	-0.11	-0.12	-0.05	0.47	0.23	0.40	-0.11	-0.05	0.28	-0.05	-0.07	-0.12	-0.07	0.20	0.34	0.23

Co-loss correlation values (* denotes significance)

	dpp	en	hh	wnt1	zen	dac	eya	eygtoe	pax6	six	efgr	glass	kr	notch	spam	spitz	vsx	arr	gq	opsin	plc
**en**	-0.20																				
**hh**	-0.07	0.35																			
**wnt1**	-0.07	0.18	0.26																		
**zen**	0.00	0.00	0.00	0.00																	
**dac**	**0.62***	-0.14	-0.22	-0.06	0.00																
**eya**	-0.30	0.39	0.45	0.22	0.00	-0.27															
**eygtoe**	**0.60***	0.03	-0.15	-0.04	0.00	**0.69***	-0.18														
**pax6**	0.39	-0.07	-0.33	-0.09	0.00	**0.68***	-0.27	**0.50***													
**six**	-0.26	0.24	0.18	0.24	0.00	-0.23	**0.63***	-0.16	-0.08												
**egfr**	0.09	0.13	0.28	-0.11	0.00	0.16	0.00	0.07	0.06	-0.02											
**glass**	-0.27	0.00	-0.15	-0.11	0.00	-0.04	0.00	-0.16	0.21	0.32	-0.17										
**kr**	0.00	0.00	0.00	0.00	0.00	0.00	0.00	0.00	0.00	0.00	0.00	0.00									
**notch**	-0.12	0.24	0.02	-0.12	0.00	-0.07	0.22	0.07	0.02	0.34	0.31	0.28	0.00								
**spam**	0.00	0.00	0.00	0.00	0.00	0.00	0.00	0.00	0.00	0.00	0.00	0.00	0.00	0.00							
**spitz**	0.00	0.00	0.00	0.00	0.00	0.00	0.00	0.00	0.00	0.00	0.00	0.00	0.00	0.00	0.00						
**vsx**	-0.18	0.04	0.37	0.38	0.00	-0.16	0.13	-0.11	-0.24	0.02	-0.09	-0.13	0.00	-0.16	0.00	0.00					
**arr**	0.11	0.03	-0.22	-0.06	0.00	0.16	0.10	0.29	0.22	0.32	0.10	0.34	0.00	0.48	0.00	0.00	-0.16				
**gq**	**0.60***	0.02	-0.15	-0.04	0.00	**0.69***	-0.18	**1.00***	**0.50***	-0.16	0.07	-0.16	0.00	0.08	0.00	0.00	-0.11	0.30			
**opsin**	0.13	0.11	-0.02	0.18	0.00	0.43	0.01	0.47	0.42	-0.13	0.06	0.13	0.00	0.08	0.00	0.00	0.21	0.19	0.47		
**plc**	-0.26	0.16	0.28	0.23	0.00	-0.05	0.40	-0.16	0.22	0.23	-0.07	0.09	0.00	0.19	0.00	0.00	0.06	0.01	-0.16	0.17	
**trp**	0.18	0.18	0.17	0.39	0.00	0.24	0.02	0.18	0.17	0.11	0.06	0.20	0.00	0.19	0.00	0.00	0.17	0.24	0.19	0.35	0.22

Net gain / loss correlation values (* denotes significance)

	dpp	en	hh	wnt1	zen	dac	eya	eygtoe	pax6	six	egfr	glass	kr	notch	spam	spitz	vsx	arr	gq	opsin	plc
**en**	0.14																				
**hh**	0.09	**0.50***																			
**wnt1**	0.02	0.22	0.02																		
**zen**	-0.33	0.07	-0.02	0.03																	
**dac**	**0.55***	0.05	0.22	0.00	-0.36																
**eya**	0.02	0.48	**0.61***	0.24	0.08	0.08															
**eygtoe**	**0.55***	0.07	-0.17	-0.02	0.02	0.41	-0.04														
**pax6**	0.41	0.06	-0.01	-0.03	-0.28	**0.56***	-0.07	0.38													
**six**	0.13	0.21	0.47	-0.07	0.07	0.08	**0.56***	-0.04	0.01												
**egfr**	0.00	0.23	0.21	-0.04	0.04	0.24	0.20	0.10	0.22	0.14											
**glass**	0.03	0.21	0.22	-0.07	0.07	0.12	-0.11	-0.04	0.11	0.14	-0.14										
**kr**	-0.02	0.07	-0.02	0.03	-0.03	0.00	0.08	0.02	0.03	-0.24	0.26	-0.27									
**notch**	0.02	**0.55***	0.35	-0.09	0.09	0.19	0.44	0.13	0.08	0.35	0.20	0.31	-0.23								
**spam**	-0.33	0.07	-0.02	0.03	1.00	-0.36	0.08	0.02	-0.28	0.07	0.04	0.07	-0.03	0.09							
**spitz**	-0.47	0.10	-0.03	0.04	**0.72***	-0.51	0.11	-0.38	-0.40	0.10	-0.10	0.10	-0.04	-0.10	**0.72***						
**vsx**	0.19	0.30	0.17	0.30	0.01	-0.02	0.15	-0.01	-0.02	-0.09	0.04	-0.04	0.01	0.05	0.01	0.01					
**arr**	0.17	0.22	0.17	-0.03	0.03	0.00	0.35	-0.02	0.13	**0.52***	-0.04	0.41	0.03	0.39	0.03	0.04	-0.01				
**gq**	**0.49***	0.12	0.12	0.03	-0.03	**0.58***	0.17	0.48	**0.54***	0.04	0.28	-0.22	0.40	0.04	-0.03	-0.32	0.13	0.03			
**opsin**	0.27	0.18	0.14	0.20	0.23	0.34	0.19	0.32	0.35	0.03	0.28	-0.12	0.27	0.05	0.23	-0.04	0.19	-0.04	**0.60***		
**plc**	0.12	0.22	0.06	0.26	0.25	-0.12	0.17	0.01	0.25	0.13	-0.06	0.12	-0.26	0.23	0.25	0.17	0.18	0.17	0.11	0.38	
**trp**	0.17	0.36	0.24	0.37	0.05	0.15	0.12	0.01	0.24	0.10	0.25	0.11	0.05	0.27	0.05	-0.20	0.23	0.11	0.08	0.36	0.32
