# Supplementary material for: Gene duplication and the origins of morphological complexity in pancrustacean eyes, a genomic approach
Source: BMC Evol Biol. 2010 Apr 30;10:123. doi: 10.1186/1471-2148-10-123 (PMC2888819; doi:10.1186/1471-2148-10-123)
Supplement: Additional file 3 — PCR conditions for the amplification of Pax6 homologs from Euphilomedes carcharodonta and Daphnia pulex. [file 1471-2148-10-123-S3.PDF]

Title: Methods for the characterization of *Daphnia pulex* and *Euphilomedes carcharodonta* Pax6 homologs.

Table S1 - Primers used for characterization of arthropod *Pax-6* genes

#	Name	Sequence
1	Pax6 Forward	GNGGNGTNTTYGTNAAYGG
2	Pax6Nested Forward	TNGGNMGNTAYTAYGARACNGG
3	Reverse	GCRAANACRTCNGGRTARTG
4	Pax6-DF1	CCTTGTGATATTCTCGAATWCTNCAAGT
5	Pax6-DR2	CCTTGCTAAGATTTTCGACACGCATCCGT
6	EuphPaxF2	GGGAATGYCCAAGCATATTCGCATGGG
8	Int6R	CGCTCTTCTATTACTAAACCAA
9	Dphn_eyF	CAGCGTTTCATCCATCAACCGTGTGTTA
10	Dphn_eyenestF	CTCGACTGCGGCTCAAGCGCAAACT
11	5RtoyR	GCTAAGATTTTCGACACGCATCCGTTTG
12	5Rtoy-nestR	TCGACACGCATCCGTTTGAGACTTGAAG
13	Dphn_toyR	GGCCAATCTTTTCGGCGAGTCGTTCA
14	Dphn_toynestR	GCAGCACGCGATTGATCGAAGAAA
15	Dphn_eyR	GAGGTGCGGTTGCGCTGGAGTTT
16	pul ey98R	GGGCTCGAGACGGAATAGTGGCGTGCTGGTGC
17	Pax6 C forward	AARCGGGARTGYCCCTCGATATTYGCRTGG
18	Pax6 Z reverse	CCATTTWGCTCGWCGRTTYGARAACCAMACCTG

Table S2– Primers and cycling parameters used to characterize arthropod *Pax-6* genes

Gene	GenBank GI	Region	Primers (Table 1)	Cycling
Euphilomedes toy	78370176	Degenerate	4,5	[94/30 + 45/30 + 72/1 min] x 45
		5' RACE	11,12	Touchdown60, Nest[1]
		3' RACE	6	[94/30 + 55/30 + 72/60] x 40 + 72/10
Daphnia toy	78370177	Degenerate	17,18[2]	[94/45 + 50/45 + 72/2] x 40 + 72/10
		5' RACE	13,14	Touchdown60, Nest[1]
		3' RACE	N/A	
Daphnia ey	78370179	Degenerate	17,18[2]	[94/45 + 50/45 + 72/2] x 40 + 72/10
		5' RACE	15,16	Touchdown60, Nest[1]
		3' RACE	9,10	Touchdown60, Nest[1]

[1] Parameters recommended by Gene Racer kit, Invitrogen

[2] Gel purified
